# Structure–Stability Correlations on Quaternary Ammonium Cations as Model Monomers for Anion-Exchange Membranes and Ionomers

**DOI:** 10.1021/acsaem.5c01293

**Published:** 2025-06-28

**Authors:** Samuel D. T. Power, Corinne Wills, Casey M. Dixon, Paul G. Waddell, Julian G. Knight, Mohamed Mamlouk, Simon Doherty

**Affiliations:** † Newcastle University Centre for Catalysis (NUCAT), School of Chemistry, 5994 Newcastle University, Bedson Building, Newcastle upon Tyne NE1 7RU, U.K.; ‡ School of Engineering, Merz Court, 5994 Newcastle University, Newcastle upon Tyne NE17RU, U.K.

**Keywords:** head groups, alkaline stability, anion exchange membranes, structure−stability correlations, computational studies, hydration level

## Abstract

Understanding the alkaline stability of quaternary ammonium (QA) cations tethered to polymer backbones in anion-exchange membranes (AEMs) is crucial to advance the long-term performance of anion-exchange polyelectrolyte-based fuel cells and electrolyzers. A library of model QA cations with *N*-phenyl and *N*-benzyl tethers has been synthesized, and comparative alkaline degradation studies revealed that the former are much less stable toward hydroxide attack than their benzylic counterparts. Density functional theory (DFT) studies support the relative stability of the QA cations and demonstrate the critical effect of hydroxide solvation on alkaline stability as well as the degradation pathway. The 3-benzyl-3,6-diazaspiro[5.5]­undecane-6-ium (*N*-benzyl-ASU, **8**) cation was found to be the most stable QA group, with a half-life of 2,595 h at 80 °C and 14,363 h at 60 °C in 3 M NaOD at a hydration number of 4.8, despite its *N*-phenyl-ASU counterpart (**5**) having a higher energy lowest unoccupied molecular orbital (LUMO); this suggests that the LUMO energy alone may not be an accurate indicator of alkaline stability. This study highlights the importance of considering the method of tethering the QA group to the polymer backbone and controlling the level of hydroxide hydration when developing QA cations for use in AEM-based devices. The structure–stability correlations arising from this work will inform the design of heteroatom donor-containing QA-based head groups with improved stability profiles

## Graphical abstract

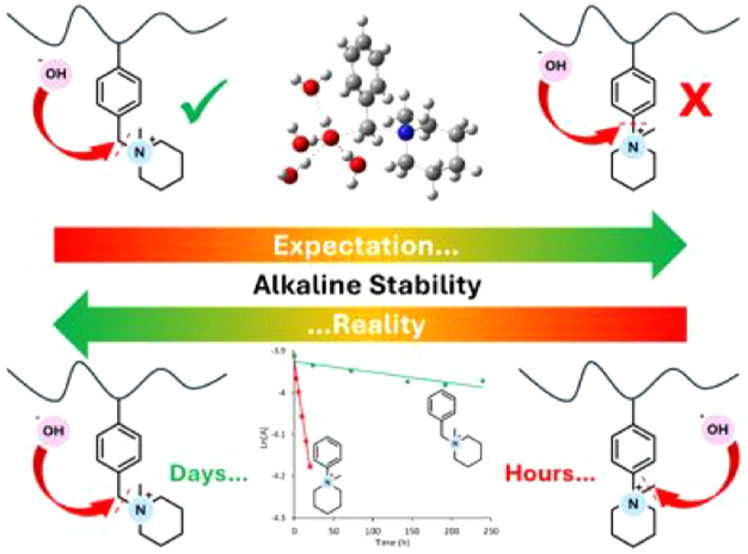

## 1 Introduction

There is a current worldwide interest in the development of sustainable and environmentally friendly energy technologies to reduce carbon emissions, slow climate change, and meet the global demand for energy.^1^ Green hydrogen is likely to play an important part in this transition, as it has a high energy density (120 MJ/kg) and can be produced sustainably using water electrolyzers (WEs) powered by a renewable source of energy (e.g., wind or solar).^2–5^ Green hydrogen is then stored and used in a fuel cell (FC) or in a gas turbine, when required, to produce clean and sustainable electricity, or combusted to provide heat, e.g., for industrial applications.^6^

FCs and WEs can operate under either acidic or alkaline conditions. Devices that operate under acidic conditions use a proton exchange membrane (PEM) and produce high current densities and efficiencies.^7^ However, the need for expensive platinum-group metal (PGM) catalysts and their oxides to catalyze the oxygen reduction or evolution reaction, as well as fluorinated polymer-based PEMs to survive the harsh acidic and oxidative conditions, has hindered their commercial implementation and raised environmental concerns.^8,9^ Alternatively, anion-exchange membrane (AEM)-based WEs (AEMWEs) are seen as a viable way forward as they use non-noble metal electrocatalysts and low cost stainless steel metal bipolar plates, which reduces the overall cost quite significantly.^6,10,11^ The AEM is typically a solid polymer electrolyte that consists of a cationic head group tethered to a polymer backbone. The organic cations are a key component of AEM-based devices, as they are the counterions that facilitate the movement of hydroxide ions, i.e., conductivity. However, even though the cations are covalently bound to the polymer backbone, they are susceptible to attack by strong nucleophiles and bases. As such, their stability in the alkaline environment of an AEMWE will determine the lifetime of the device, which must operate at high temperatures and high pH for tens of thousands of hours. Thus, an effective AEM must remain intact under the high-temperature and high-pH operating conditions of an AEM device, i.e., have good chemical and mechanical stability while maintaining high hydroxide conductivity with a stable profile, as well as be resistant to excessive swelling and have low permittivity.^12,13^

A wide range of organic cations has been explored as potential head groups for use in AEMs,^14,15^ and common examples include quaternary ammonium,^16–18^ cyclic ammonium,^19–21^ phosphonium,^22,23^ sulfonium,^24^ imidazolium,^25–27^ benzimidazolium,^28,29^ pyridinium,^30,31^ and guanidinium-based cations.^32^ The alkaline stability of these cations has been thoroughly investigated,^12,13,33–37^ and a number of degradation pathways have been identified, including nucleophilic dealkylative substitution (S_N_2), Hofmann elimination (E2), ylide formation, Sommelet–Hauser rearrangement, and Stevens rearrangement.^38,39^ It is possible to design cationic head groups with structural features to reduce the likelihood of base-induced degradation. For example, substituting the beta-hydrogens with an alkyl group removes the possibility of E2 degradation,^40^ the inclusion of electron-donating groups helps reduce susceptibility to hydroxide attack by stabilizing the cationic center,^41,42^ and delocalization of the positive charge renders the cation less susceptible to nucleophilic attack.^43^ However, building in stability is often synonymous with more complexity, which necessarily involves increasingly intricate and challenging synthesis as well as rising costs, which will ultimately limit scalability for manufacture and commercialization. The most common head groups are quaternary ammonium (QA) cations due to their relatively straightforward, modular, and adaptable synthesis; however, their low alkaline stability has limited their commercial application.

The stability of QA cations is greatly influenced by the nature of the four alkyl groups bonded to the quaternary *N*-center, at least one of which is used as a “tether” to attach the head group to the polymer backbone. While selected QAs have demonstrated appropriate alkaline stability as a stand-alone head group, for example, the spirocyclic 6-azoniaspiro­[5,5]­undecane (ASU) motif,^17^ it is synthetically challenging to modify this structure to introduce a polymerizable group that would enable direct polymerization to access anion-exchange polyelectrolytes (AEPs) while maintaining comparable stability. To this end, there are reports of ASU-based poly­(arylene piperidinium) AEMs that have very high alkaline stability and good conductivity profiles, although their synthesis involves postpolymer modification of the corresponding poly­(arylene alkylene piperidinium).^44^ However, it may be possible to prepare this class of AEM directly through the acid-catalyzed polymerization of 2,2,2-trifluoro-1-(4-piperidine)­ethenone-derived ASU-based head groups with a phenylene-based comonomer, as an alternative to postpolymer modification. Moreover, even though degradation studies on model head groups have successfully identified candidates with promising stability profiles, incorporation of the cation into a polymer has been shown to increase the rate of degradation. This has been attributed to several factors, including the introduction of new degradation pathways by tethering the head group to the polymer backbone,^45^ backbone rigidity-induced distortion of the ring conformation facilitating ring-opening Hofmann elimination,^46,47^ and polymer motif-specific degradation, e.g., poly­(arylene ether), polysulfone, and para-methine-substituted polystyrene.^13,48,49^ This highlights the importance of designing the head group, the polymer backbone, and the tether connecting the two to be chemically inert. To this end, one of the most common approaches to attach a head group to a polymer backbone has been through the methylene carbon of a benzyl ring, as the corresponding monomers are either readily accessible or the backbone can be modified using well-developed protocols such as chloromethylation. However, the use of this tether can be detrimental to the AEM, as it is susceptible to irreversible degradation via S_N_2 substitution by hydroxide at the benzylic CH_2_ as well as 1,6-Hofmann-type elimination in poly­(vinylbenzylammonium)-based architectures.^16,33,35,48,49^

Designing a QA head group with a polymer tether that does not incorporate a benzyl group may result in increased alkaline stability. To this end, we reasoned that the replacement of the benzyl group with a phenyl group would remove the nucleophilic debenzylation degradation pathway and thereby improve head group alkaline stability. Thus, we have prepared a series of aryl amine-based QA model head groups and compared their stability against their benzylic counterparts to establish whether direct attachment of the cation to an aryl ring improves stability. Moreover, the synthesis of aryl amine-based head groups can be achieved using the Buchwald–Hartwig (BH) amination, which is a universally reliable, highly versatile, and well-developed catalytic C–N bond-forming protocol that has been widely adopted by the synthesis community.^50,51^ In addition, the BH amination would lend itself to the construction of a library of potential QA model head groups via automated high-throughput technology to develop structure–stability relationships and identify cations that are resistant to degradation and potentially suitable for incorporation into AEPs. In addition, once a model substrate with a promising stability profile has been identified, it would be straightforward to apply the same protocol to prepare the corresponding styrene-based monomer for the rational and systematic synthesis of copolymers with different compositions and loadings; this would be a distinct advantage over the more common approach of postpolymerization modification, which has several inherent disadvantages including challenges in characterization and quantifying the loading at each stage of modification, as well as the difficulty of diversifying the architecture and challenges around scale due to the large volumes of solvent required and the low concentration of polymer used. Although the bottom-up approach to developing AEPs involving the synthesis of head groups as monomers offers several potential advantages over postpolymer modification, it would need to be cost-effective for commercialization, i.e., require low catalyst loadings or the use of an inexpensive copper-based catalyst for the Buchwald–Hartwig amination,^51^ and involve operationally minimal and straightforward processing and purification procedures.

Herein, we report the synthesis of nine phenyl- and benzylammonium-based model head groups and a comparison of their stability, which revealed that direct attachment of the ammonium cation to a phenyl ring results in a dramatic decrease in alkaline stability compared with their benzylic counterparts. Similarly, the stability profiles of spirocyclic and *N,N*-dimethyl-based piperazinium model head groups is markedly dependent on the tertiary amine, as those with a *N*-phenyl group are also much less stable than their *N*-benzyl counterparts. DFT calculations at different levels of hydration support the relative stability of these QA cations and demonstrate the dramatic influence of hydroxide solvation on their alkaline stability, as well as the major degradation pathway. From this work, a design description has been established that will guide the future development of stable QA head groups and their attachment to a polymer.

## 2 Experimental Section

### 2.1 Alkaline Degradation Studies on Model Head Groups 1–10

Adopting a recent literature protocol,^52^ a typical experimental procedure for alkaline stability studies is described below. A solution of 3 M NaOD/D_2_O/CD_3_OD (1.5375 g NaOD 40 wt %, 0.514 g D_2_O and 2.927 g CD_3_OD, corresponding to a D_2_O:CD_3_OD mole ratio of 3:7 and a hydration number (λ) of 4.8) was prepared for each head group to be tested. To this solution, 0.1 mmol of QA salt and 0.05 mmol of 3-(trimethylsilyl)-1-propanesulfonic acid sodium salt (NaDSS) were added. The ^1^H NMR spectrum was recorded immediately to represent a time of 0 h. The solutions were then heated to either 60 or 80 °C, and ^1^H NMR spectra were recorded at set time intervals up to a total of 240 h.

The ratio of degradation (QA:NaDSS) was calculated by comparing the relative integral area of a chosen QA peak to that of the NaDSS standard (Figure S26, 0.60 ppm, 2H). The ratio of degradation was calculated by one of the following two methods based on the extent of degradation observed. Where degradation product(s) were observable, the ratio of degradation was determined by comparing the relative integral area of a chosen degradation peak against that of NaDSS. Where no degradation product(s) were observable, the ratio of degradation was calculated by comparing the relative integral area of a chosen QA peak against that of NaDSS.

### 2.2 Calculations

All density functional theory (DFT) calculations were performed using the Gaussian 09 package.^53^ All structures were optimized using the B3LYP hybrid functional.^54–56^ Optimizations were carried out with the 6-311++G­(2d,p) basis set.^57–59^ Each optimized geometry was verified through frequency calculations. Transition states were optimized using the Berny algorithm and confirmed to have a single negative frequency. Calculations were computed with the polarizable continuum solvent model using water as the solvent (ε = 78.36),^60^ and explicit water molecules were added individually to simulate different hydroxide hydration numbers. All calculations were performed at 60 °C to replicate the experimental conditions.

## 3 Results and Discussion

### 3.1 Synthesis of Model Head Groups 1–9

Alkaline stability studies on model cationic compounds have been shown to be a powerful approach to understanding degradation pathways and identifying motifs that are resistant to decomposition.^12,19,25,61,62^ While the *N*-benzyl fragment is commonly used to attach cationic head groups to a polymer due to its ready accessibility, the connecting methylene fragment introduces a pathway for cleavage of the cation via S_N_2-based nucleophilic debenzylation.^16–18,49^ Reasoning that replacement of the *N*-benzyl group with *N*-phenyl should improve cation stability by eliminating this degradation pathway, *N*-phenylammonium cations **1**–**6** and *N*-benzylammonium cations **7**–**9** were prepared as models for the corresponding styrene-based head groups to conduct comparative alkaline degradation studies; the structures and details for the synthesis of **1**–**3** and **5**–**9** are shown in Scheme 1. Finally, 3-benzyl-6-azaspiro[5.5]­undecane-6-ium bromide (**10**) was also prepared and used as a literature benchmark standard to assess the influence of alkaline stability on the presence of the tertiary amine in the piperazinium ring.

**Scheme 1:**
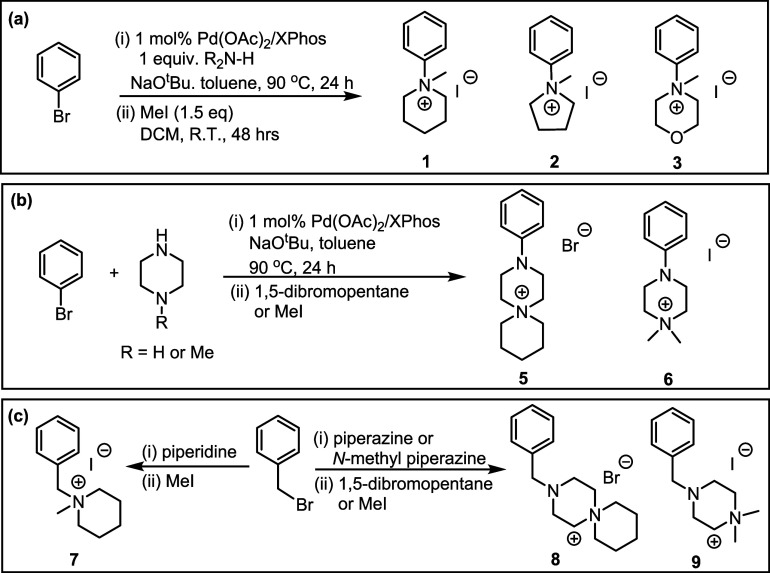
Synthesis of Model Quaternary Ammonium Head Groups (a) **1**–**3**, Where R_2_N–H = Piperidine, Pyrrolidine, or Morpholine, (b) **5**–**6** and (c) **7**–**9** Explored in This Study

This hypothesis was investigated by performing preliminary DFT calculations, as studies have reported a qualitative correlation between the LUMO energy and the stability of quaternary ammonium cations, with a higher LUMO energy translating to a higher alkaline stability.^41,42^ To this end, the effect of the *N*-phenyl group on alkaline stability was explored by calculating the energies of the LUMOs for **1**–**9**. Although the LUMOs for *N*-phenyl-substituted QA cations **5** and **6** were higher in energy than their *N*-benzyl counterparts **8** and **9**, those for **1** and **7** are similar, which suggests that the use of the LUMO energy as an indicator of alkaline stability should be treated with caution. The piperazine motif in cations **5**–**6** and **8**–**9** was chosen because cyclic ammonium cations have been proposed to be more stable than their long-chain alkyl counterparts, as Hofmann elimination is conformationally disfavored,^47,62,63^ while the additional nitrogen heteroatom would facilitate attachment of head groups with promising stability profiles to a polymer backbone, either via the derived styrene-based monomer or by postpolymerization modification.

*N*-Phenylammonium cations **1**–**3** were prepared via a Buchwald–Hartwig amination between bromobenzene and the appropriate secondary amine to afford the corresponding *N*-phenylamine which was subsequently quaternized by the reaction with methyl iodide to yield **1–3,** and 4 was synthesized by methylation of dimethylaniline (Scheme 1a). Similarly, quaternary ammonium cations **5**–**6** were prepared via a selective Buchwald–Hartwig amination between bromobenzene and either piperazine (*R* = H) or *N*-methylpiperazine (*R* = Me) followed by the reaction of the derived amine with 1,5-dibromopentane or methyl iodide to afford cations **5** and **6**, respectively (Scheme 1b). *N*-Benzylammonium cations **7**–**9** were prepared via S_N_2 substitution between benzyl bromide and the appropriate secondary amine, followed by subsequent quaternization using methyl iodide for **7** and **9** and 1,5-dibromopentane for **8** (Scheme 1c) and used to perform comparative stability studies against their *N*-phenyl counterparts, **1**, **5**, and **6**, respectively. All model monomers were characterized by ^1^H and ^13^C NMR spectroscopy and mass spectrometry (see Figures S1–S18), and head groups **1**, **2**, **4**, **5**, **6**, **7**, and **9** were characterized by single-crystal X-ray crystallography. The structures of **1** and **7** are shown in Figure 1a,b, and full details for all structures are provided in Figures S19–S25 and Tables S1–S7.

**Figure 1:**
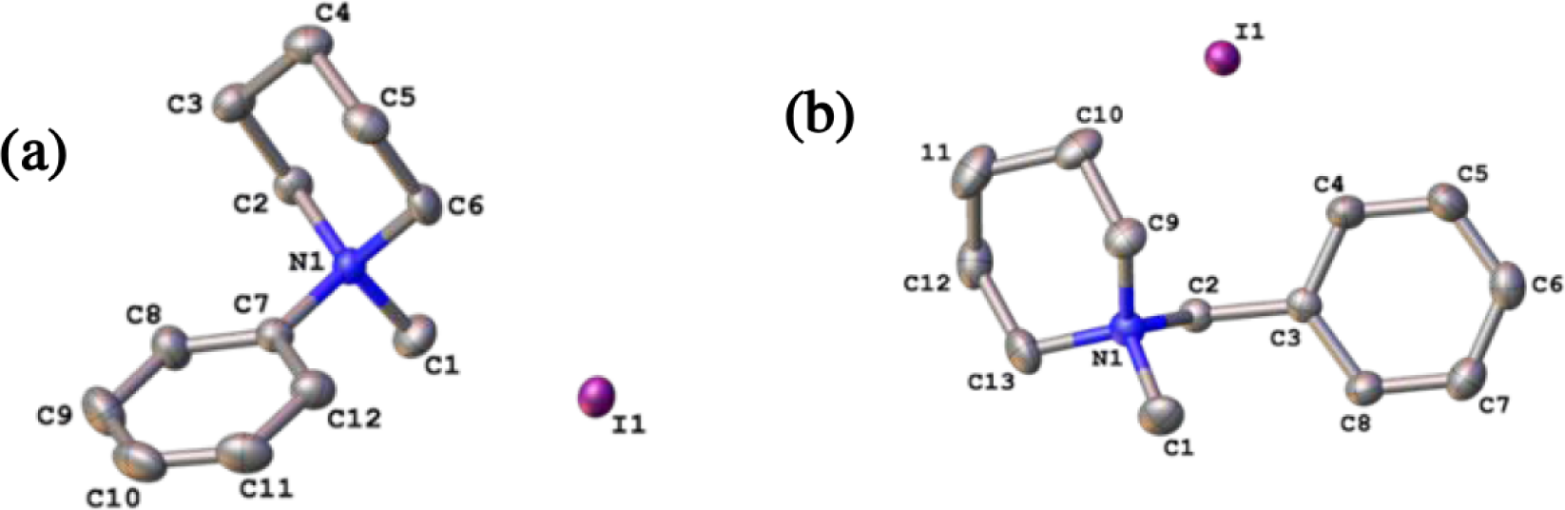
Single-crystal X-ray structures of (a) **1** and (b) **7**. Displacement parameters are drawn at the 50% probability level, and hydrogen atoms are omitted for clarity.

### 3.2 Degradation Studies

Although degradation studies on model compounds provide quantitative data on the stability of cations and have been used to identify key features that are resistant to decomposition,^13,27,52^ it is difficult to compare the results of stability studies across the literature due to the disparate conditions under which alkaline degradation experiments are conducted, including the solvent, temperature, and hydration number.^64,65^ To this end, the stability of cations **1–9** was examined by heating the model head groups in 3 M NaOH/D_2_O/CD_3_OD, corresponding to a hydration level of 4.8, following a standard protocol recently reported to investigate the stability of 24 *N*-heterocyclic ammonium compounds.^52^ Although these conditions are not as harsh as the ultralow hydration levels that might be encountered at the cathode of an AEMFC operating at high current densities,^66,67^ the applied conditions are expected to lead to the same cation degradation pathways as in a working electrolyzer. Moreover, the hydration number associated with this protocol has been widely adopted, which should enable qualitatively reliable comparisons with other studies. In addition, the motifs of some of the synthesized cations are similar to those reported herein and, as such, will provide an informative comparison. Alkaline stability studies were conducted at either 60 or 80 °C using sodium 3-(trimethylsilyl)­propane-1-sulfonate as an internal standard because it does not undergo H/D exchange, as shown by a ^1^H NMR study conducted in an alkaline solution at 60 °C as a function of time over 240 h (Figure S29). The decomposition was mapped by analysis of ^1^H NMR spectra recorded at regular time intervals to identify decomposition products and determine the degradation pathways; the extent of degradation was calculated by integration of the internal standard against the model head group at set time intervals. Integrations were obtained on protons that were clearly identifiable and that did not undergo rapid H/D isotope exchange as this would result in a loss of ^1^H signal intensity; these resonances were chosen as they would provide the most reliable measure of the amount of cation in solution. The problems arising from H/D exchange when using ^1^H NMR spectroscopy to quantify the degradation of quaternary ammonium cations in basic solution have been thoroughly documented.^12,16,18,68^ For each model head group examined, we are confident that the protons used to monitor the degradation and determine the amount of cation remaining do not undergo H-D exchange, as the ratio of the integral for these protons to that of the aromatic resonances remained constant as a function of time across 240 h; full details are provided in Table S8. However, model monomer **7** proved to be an exception, as analysis of the ^1^H NMR spectra for the degradation mixture at time 0 h and after 240 h clearly showed that the benzylic protons underwent significant H/D exchange, as evidenced by a reduction of 62% in the integral ratio between the benzylic CH_2_ and the aromatic multiplets as well as the appearance of a separate high field-shifted signal with an isotope shift of 20 ppb associated with the CHD isotopomer and the appearance of an additional broad signal at δ 61.8 ppm in the ^13^C­{^1^H} NMR spectrum with a secondary isotope shift of 42 ppb (Figures S55 and S70). This is consistent with previously reported data and confirms that H/D exchange is rapid at the benzylic position.^16^ In this case, the resonance associated with the γ-methylene protons of the piperidinium ring was used to determine the amount of cation remaining. The results of kinetic studies on each of the tested cations are shown in Figure 2 as logarithmic plots of the concentration of the cation remaining as a function of time, and a summary of the half-life for each cation is presented graphically in Figure 3 and summarized in Table S9. As benzylammonium cations have been reported to be unstable under alkaline conditions,^16–18,49^ a comparison of the stability of *N*-phenylammonium models **1**, **5**, and **6** with their benzyl counterparts **7**, **8**, and **9** would provide a direct measure of the influence on stability of replacing *N*-benzyl with *N*-phenyl. Furthermore, a comparison of the stability of **2**, **3**, and **4** against **1** would provide a measure of the influence of ring size, the introduction of an additional heteroatom donor, and cyclic versus alkyl-based QA centers on stability.

**Figure 2:**
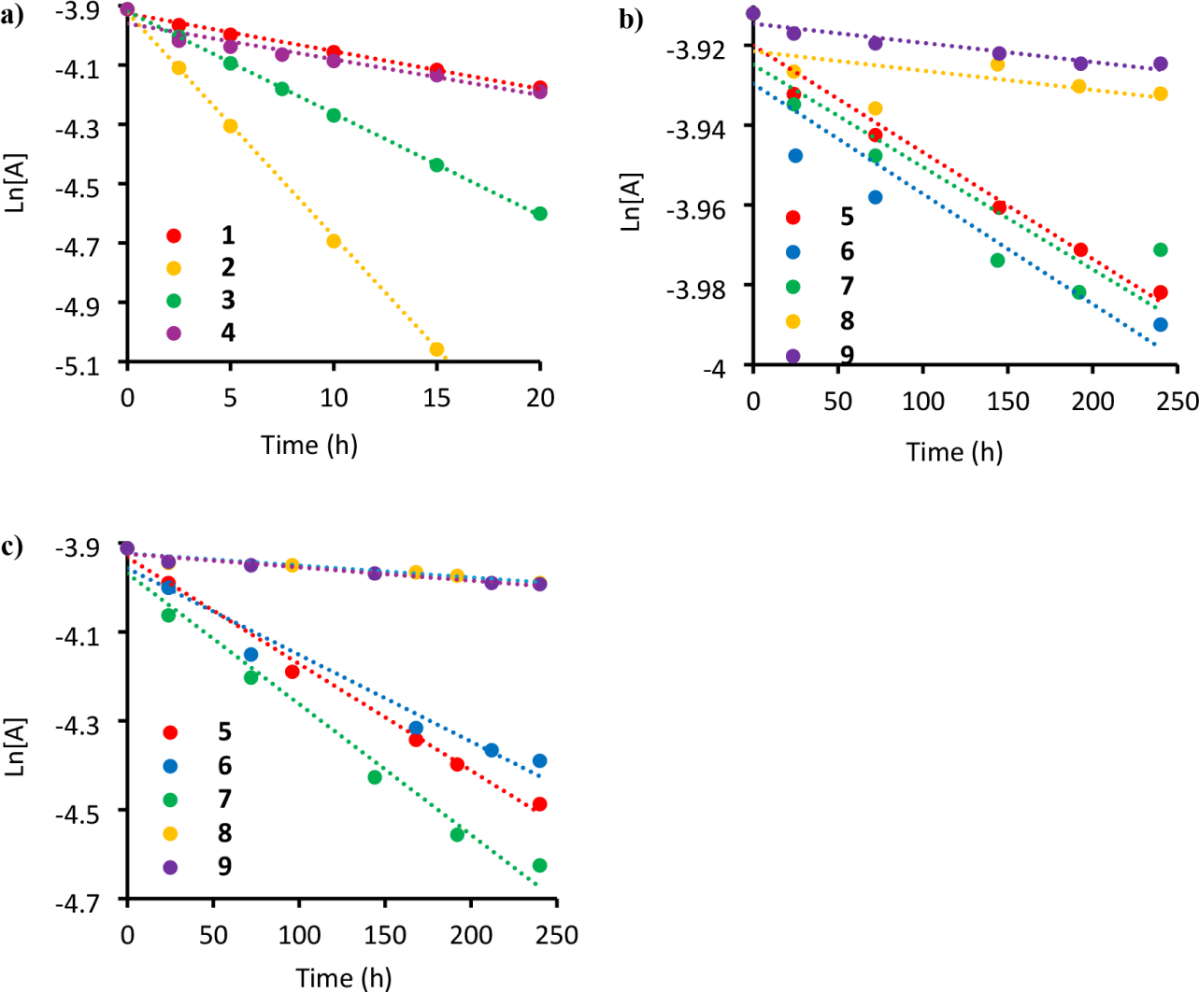
Remaining model QA cation as a function of time at λ = 4.8. The experimental data are fitted with linear trends. (a) Ln­[A] v time for **1**–**4** at 60 °C; (b) ln­[A] v time for **5**–**9** at 60 °C; (c) ln­[A] v time for **5**–**9** at 80 °C. Each data point is an average from three measurements, with errors ranging from ± 0.2% to ± 0.5%.

**Figure 3:**
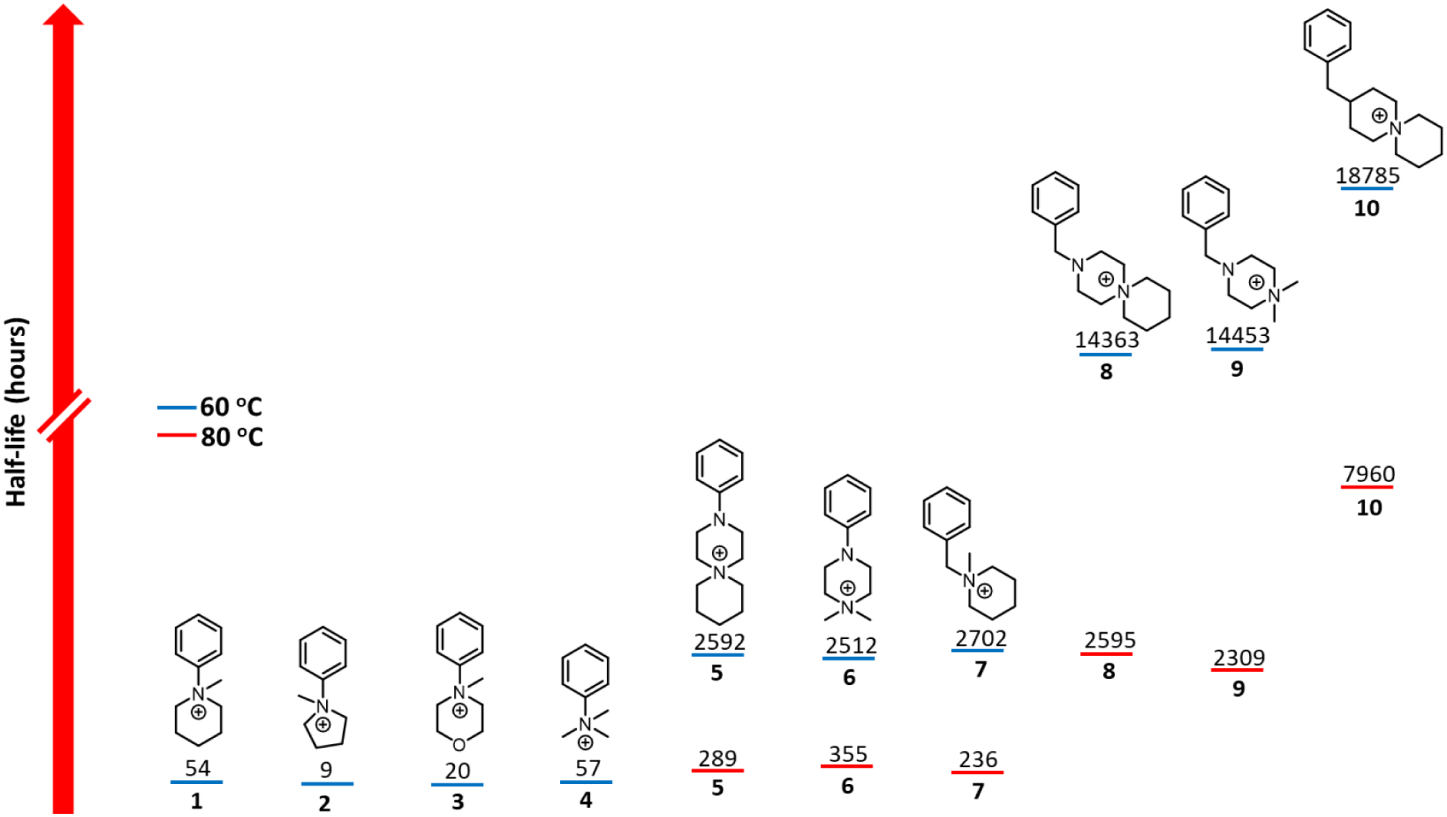
Calculated half-lives for QA cations **1–10** in 3 M NaOD/D_2_O/CD_3_OD, λ = 4.8, at 60 and 80 °C. Each calculated half-life is determined from three independent measurements and carries an associated uncertainty of ±8–10%.

The main degradation pathway for the benzylammonium cation **7** was confirmed by ^1^H NMR spectroscopy to be nucleophilic debenzylation by attack at the benzylic position, rather than at the methyl or cycloalkyl carbon, as expected from previous literature reports (Figure 4b).^16,17,27,48,60,62^ However, contrary to our design principle, comparison of the stability profiles for **1** and **7** (Figure 2) revealed that substitution of the *N*-benzyl group for *N*-phenyl resulted in a dramatic decrease in alkaline stability, as evidenced by the half-lives of 54 h and 2,702 h, respectively, at 60 °C (Figure 3). Thus, removal of the most facile degradation pathway does not improve the overall stability profile, as alternative pathways become markedly more accessible (see later). To this end, analysis of the ^1^H NMR spectra from the stability study with **7** at 60 °C revealed *N*-methylpiperidine and benzyl alcohol to be the only detectable byproducts (Figure 4b), confirming attack of hydroxide at the benzylic carbon to be the major degradation pathway. However, it is not possible to unequivocally rule out degradation by nucleophilic debenzylation with methoxide (OCD_3_),^61^ as the resulting benzylmethyl ether (Bn-OCD_3_) would have a ^1^H NMR profile similar to that of benzyl alcohol; unfortunately, neither product was observed by LC-MS. In comparison, the ^1^H NMR spectra from the corresponding degradation study on **1** contained methanol and *N*-phenylpiperidine as the only products, consistent with facile S_N_2-based demethylation by hydroxide (Figure 4a). The much higher rate of demethylation of **1**, compared to that of **7**, may be the result of conjugative stabilization of the S_N_2 transition state by the adjacent π-system of the *N*-phenyl ring. This effect is not present in the corresponding demethylation of the benzylic cation **7**, and in that case, the debenzylation pathway, which also benefits from this electronic effect, is certainly more sterically hindered than demethylation. The influence of this steric difference is manifested in the distinct nonlinearity between the nucleophile and nucleofuge in the calculated transition state for debenzylation of **7** (see later discussion and Figure 7b) compared to the much more obviously linear trajectory in the corresponding transition state for demethylation of **1** (Figure 7a). Such a difference attests to the greater steric influence of the Ph ring in the benzyl derivative. In addition, the inductive withdrawing nature of *N*-Ph, due to the carbon atoms being sp^2^ hybridized, is likely to facilitate hydroxide-based S_N_2 dealkylative C–N bond scission and/or Hofmann elimination. The introduction of a “spacer” −CH_2_ group between the aromatic ring and the QA center in **7** provides a shielding effect, resulting in reduced inductive destabilization. These factors demonstrate the need to carefully design the structure of the QA-polymer tether combination.

**Figure 4:**
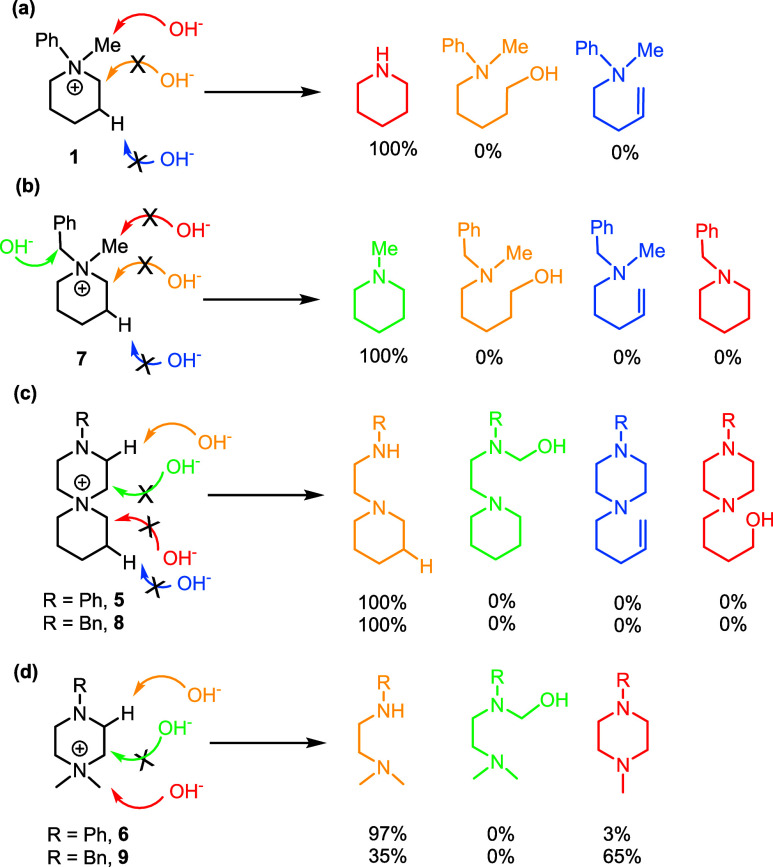
Possible nucleophilic dealkylation and E2 degradation pathways for the (a) *N*-phenyl-*N*-methylpiperidinium (**1)**, (b) *N*-benzyl-*N*-methylpiperidin-1-ium (**7**), (c) 3-phenyl-3,6-diazaspiro[5.5]­undecan-6-ium (**5**) and 3-benzyl-3,6-diazaspiro[5.5]­undecan-6-ium bromide (**8**), and (d) 1,1-dimethyl-4-phenylpiperazin-1-ium (**6**) and 1,1-dimethyl-4-benzylpiperazin-1-ium (**9**) cations at 60 °C, under λ = 4.8 conditions.

**Figure 5:**
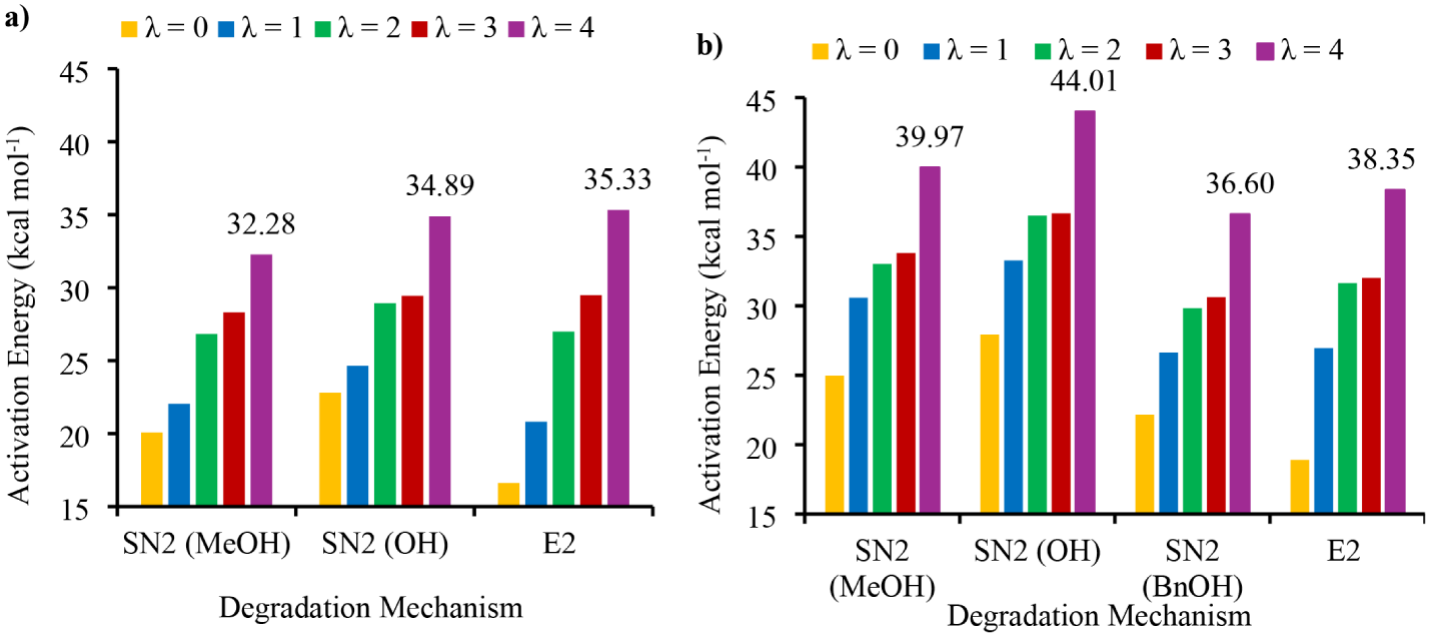
Calculated activation energies (Δ*G* ^⧧^) as a function of the hydration level (HL, λ = 0, 1, 2, 3, 4) for (a) S_N_2 demethylation, S_N_2 ring-opening dealkylation, and E2 elimination for cation **1** and (b) S_N_2 demethylation, S_N_2 ring-opening dealkylation, S_N_2 debenzylation, and E2 pathways for cation **7**.

**Figure 6:**
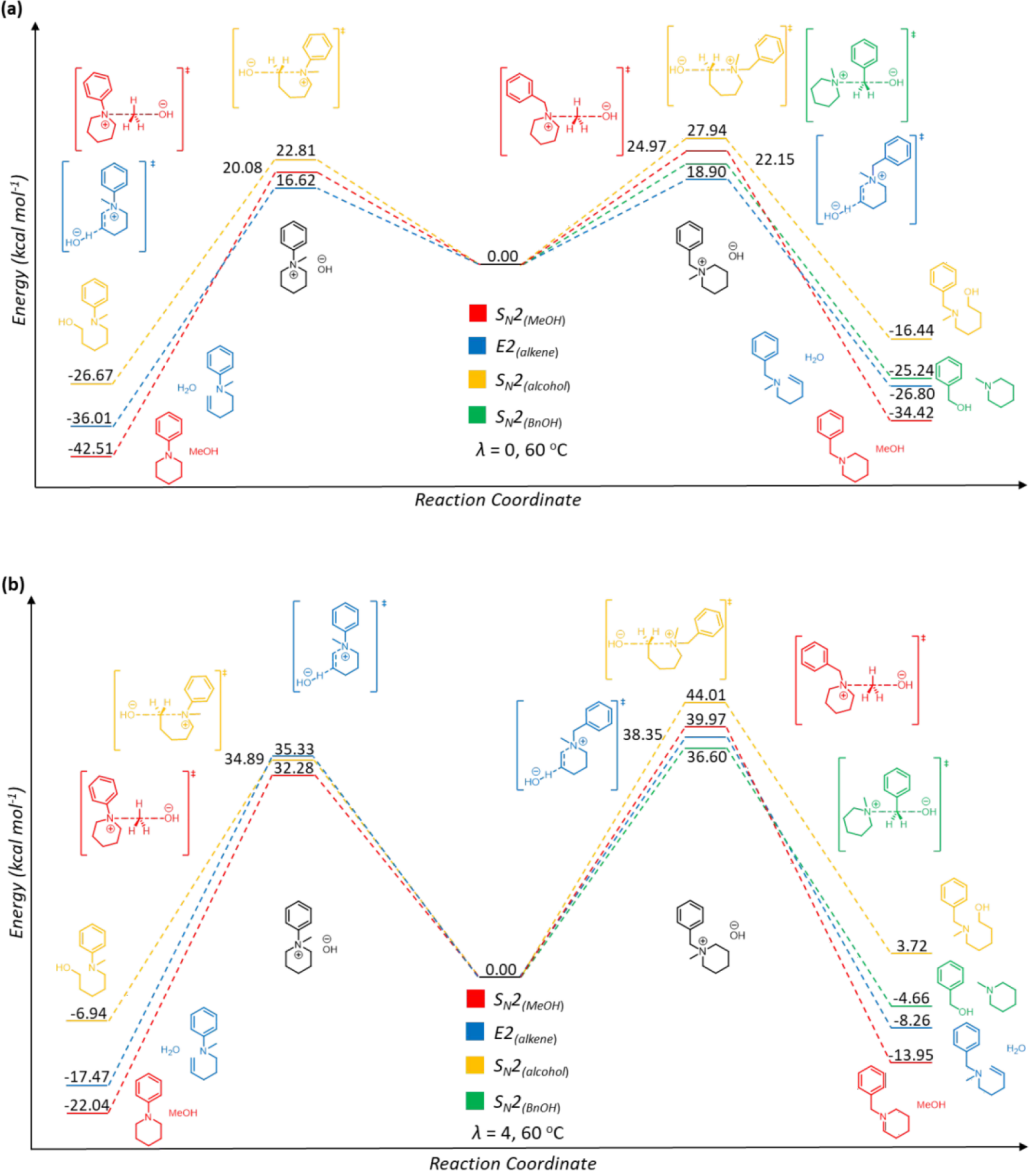
Comparison of the Gibbs free energy profile for the demethylation (S_N_2_MeOH_), ring-opening dealkylation (S_N_2_alcohol_), and E2 elimination (E2) degradation pathways for quaternary ammonium cations 1 and 7 calculated at 60 °C and hydration level (a) λ = 0 and (b) λ = 4.

**Figure 7:**
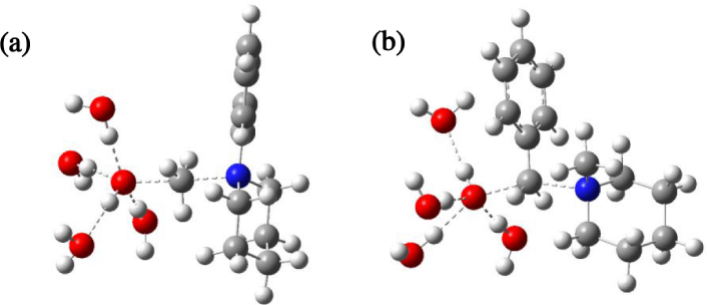
Optimized transition states of experimentally observed degradation pathways for (a) **1** (demethylation) and (b) **7** (debenzylation) at λ= 4.

As comparisons between literature data are often considered unreliable, the stability of **7** was also evaluated at 80 °C under λ = 4.8 conditions. Under these conditions, debenzylation remained the only degradation pathway, and the determined half-life of 236 h was close to the 281 h reported by Lee and Fan,^52^ which lends credibility to comparisons between this work and the reported data.

A similar degradation study between *N*-phenyl-substituted *N*-ASU **5** and *N*-DMPz **6** revealed that the piperazinium motif, in which the ammonium center is distal or more remote from the phenyl ring, improves cation stability quite dramatically, as the half-lives of 2,593 h and 2,512 h, respectively, at 60 °C and λ = 4.8, are considerably longer than the 54 h obtained for **1**. This improvement in stability is consistent with previous reports that a significant enhancement in conductivity and alkaline stability can be achieved by incorporating a long flexible alkyl tether between the QA cation and a polystyrene or polyphenylene backbone.^69,70^ Moreover, the increased stability of **5** and **6** enabled degradation studies to be conducted at 80 °C, which more closely resembles electrolyzer operating temperatures; under these conditions, the half-lives of 289 and 355 h, respectively, surpass those reported for *N*-benzyltrimethylammonium (172 h) and *N*-benzylmethylpiperidinium (281 h) at λ = 4.8.^52^ Even though **5** and **6** have markedly greater alkaline stability than **1**, both were significantly less stable than their *N*-benzylammonium counterparts, **8** and **9**, which have half-lives of 14,363 h and 14,453 h, respectively, at 60 °C and 2,595 h and 2,309 h at 80 °C. The enhancement in alkaline stability for *N*-benzyl-substituted piperazinium cations **8** and **9** compared with their *N*-Ph analogues **5** and **6** may result from the difference in their inductive effects, as the more electronegative *N*-Ph fragment would be expected to facilitate Hofmann elimination by increasing the acidity of the beta-hydrogen atoms, as well as having a larger destabilizing effect on the QA center.^71^ Additional conformational effects on the piperazinium ring, caused by the increased planarity of *N*-Ph compared to *N*-Bn, cannot be ruled out. Unfortunately, an analysis of the detailed conformational energy landscape of the two systems is unlikely to provide much additional insight, since relative ground-state differences cannot necessarily be extrapolated to the corresponding relative transition-state effects. Moreover, as the electronic and steric/conformational effects are tightly coupled in these systems, it will be extremely challenging to deconvolute and separately analyze the contribution of these effects toward calculated differences in activation barriers. However, a synthesis and computational study on a series of further substrates, in which the aromatic ring (Ph or Bn) is substituted with remote, electronically distinct groups (e.g., in the *para*-position), which might be assumed to have a negligible, steric, and conformational effect on the six-membered ring while still influencing the electronic properties, could be used to explore the role of electronic effects.

Analysis of the degradation mixture for **5** and **8** by ^1^H NMR spectroscopy revealed that both decomposed by Hofmann elimination at the piperazinium ring as the sole pathway (Figure 4c), as evidenced by the appearance of resonance characteristic of the substituted ethylenediamine motif (**II**) resulting from hydrolysis of the corresponding enamine (**I**), as shown in Scheme 2. This was confirmed by analysis of the reaction mixture using positive-mode electrospray ionization mass spectrometry (see Figures S42–S46 and S57–S61 for full details). Moreover, the absence of vinylic signals in the ^1^H NMR spectra for the decomposition of either **5** or **8** confirmed that Hofmann elimination at the piperidinium ring does not occur. In contrast, while **6** degraded primarily via Hofmann elimination to afford *N,N*-dimethyl-*N*-phenylethane-1,2-diamine, with only a minor amount of 1-methyl-4-phenylpiperazine (<3% of the degradation products) resulting from demethylation, the degradation of **9** gave more 1-methyl-4-benzylpiperazine than *N*-benzyl-*N*,*N*-dimethylethane-1,2-diamine indicating that S_N_2 demethylation (65%) is notably faster than E2 elimination (35%) when the *N*-Ph fragment is replaced by *N*-Bn (Figure 4d). The presence of 1-methyl-4-phenylpiperazine and 1-methyl-4-benzylpiperazine in the final decomposition mixtures for **6** and **9**, respectively, was confirmed by electrospray ionization mass spectrometry (see Figures S45 and S60) and comparison of the ^1^H NMR spectra with authentic samples of the piperazine (Figures S47–S51 and S62–S66).

**Scheme 2:**
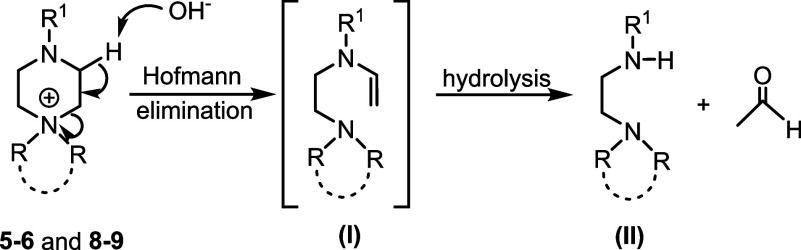
Decomposition of Cations **5**, **6**, **8**, and **9** via Hofmann Elimination and Hydrolysis of the Resulting Enamine (**I**) to Afford Diamine (**II**)

The β–H–C–C–N dihedral angles of 175.86°/176.00° and 177.65°/179.24° in **6** and **9**, respectively, determined from the X-ray crystal structures, are close to the corresponding angles of 175.01°/176.08° and 175.74°/175.81° for the calculated optimized structures and are appropriate for antiperiplanar elimination. However, the differences are relatively small and unlikely to account for the different product distributions obtained from the degradation of **6** and **9**. Although the dominant degradation pathways for **6** and **9** are different, the change in the activation barriers required to affect this product distribution will be relatively small (*ca*. 1–1.5 kcal mol^–1^). Thus, such a minor change in the activation barrier for degradation is perhaps not too surprising, as the steric, electronic, and geometrical properties of **6** and **9** are clearly dissimilar in that the former is a conjugated aniline derivative, while the latter is a tertiary amine. Further calculations on model head groups **6** and **9** at different hydration levels, combined with detailed degradation studies on, for example, electronically distinct *para*-substituted *N*-phenyl-dimethylpiperazinium cations, will be required to start deconvoluting how these properties influence the activation barriers for the various degradation pathways.

In a recent related study reporting the degradation of organic cations under alkaline conditions, the faster degradation of a spirocyclic *N*-piperazinium cation compared with the corresponding ASU cation was attributed to acceleration of the ring-opening Hofmann elimination by the extra nitrogen atom in the piperazinium ring.^61^ Thus, while the amine moiety in piperazinium-based head groups could be a useful functionality to access monomers for the preparation of polymeric membranes or as a linker for postpolymer modification, a judicious choice of connector will be required, as is evident from the disparate stability profiles of the *N*-Ph and *N*-Bn-based model head groups. The slightly higher stability of **8** compared with **9** at 80 °C, as evidenced by the half-lives of 2,595 h and 2,309 h, respectively, is consistent with the spirocyclic piperidinium motif, which has been reported to be more stable than the corresponding *N,N*-dimethyl-substituted piperidinium cations.^16,20,46,52,62^

A direct comparison of the stability profiles for piperidinium **1** and pyrrolidinium **2** revealed that the latter degrades faster, with a half-life of only 9 h at 60 °C compared with 54 h for **1**. This observation is consistent with the trend reported for five- and six-membered *N*-spirocyclic cations,^61^ as well as a recent computational study that analyzed the degradation mechanisms of a series of heterocyclic QA cations.^62^ While *N*-phenyl-*N*-methyl piperidinium **1** degraded via S_N_2-based demethylation, its pyrrolidinium counterpart also underwent hydroxide- and methoxide-based S_N_2 ring-opening to afford 4-(methyl­(phenyl)­amino)­butan-1-ol and d_3_-*N*-(4-methoxybutyl)-*N*-methylaniline, respectively. These products were identified by the presence of molecular ions at *m*/*z* = 180 and 197, respectively, together with the expected fragmentation patterns in the electrospray ionization mass spectra (see Figures S30–S35). As the ^1^H NMR spectra for the products resulting from nucleophilic ring opening by hydroxide and d_3_-methoxide are very similar, it is difficult to establish which is the major product in the absence of further analysis. However, the disparate intensities of the molecular ions associated with the two ring-opened products in the mass spectrum suggests nucleophilic degradation by methoxide to be the dominant pathway, consistent with recent degradation studies of organic cations in KOH/CD_3_OH.^61^ This is perhaps expected due to the higher basicity of methoxide relative to hydroxide and the presence of both in large excess in the degradation solution. Interestingly, this is the only head group that undergoes S_N_2 ring-opening dealkylation, which is most likely due to the higher ring strain compared to its six-membered counterpart.

The *N*-phenyl *N*-methylmorpholinium cation **3** was also prepared for comparison with **1** on the basis that a derived membrane would exhibit increased conductivity due to an expanded hydration sphere surrounding the organic cation.^72^ However, **3** also degraded rapidly at 60 °C (*t*_1/2_ = 20 h) via S_N_2-based demethylation as the major pathway, as *N*-phenylmorpholine and methanol were identified in the ^1^H NMR spectrum and the electrospray ionization mass spectrum of the final degradation mixture (see Figures S36–S38). A minor amount of vinyl ether degradation product was also observed, as evidenced by a set of signals at δ 6.45 and 4.01 ppm in the ^1^H NMR spectrum and a molecular ion peak at 178.1 in the electrospray ionization mass spectrum. Degradation via Hofmann elimination most likely arises from the increased acidity of the beta-hydrogen atoms adjacent to the oxygen in the morpholinium ring, compared with those in the piperidinium ring of **1**,^73^ and is consistent with previous reports and computational investigations.^61,62^ The dramatic difference in stability between *N*-Ph-based QA cations and their *N*-benzyl counterparts also applies to the *N,N,N-*trimethylanilinium cation **4**, which has a half-life of only 57 h at 60 °C under λ = 4.8 conditions, whereas the analogous [NBnMe_3_]^+^ is markedly more stable with a reported half-life of 172 h at 80 °C at the same hydration level.^52^

While the initial hypothesis was to replace the *N*-benzyl fragment in quaternary ammonium cations with *N*-phenyl to improve stability, based on the fact that the benzyl group is highly susceptible to debenzylation by S_N_2 attack, comparison of the stability profiles for **1**, **5**, and **6** with those for **7**–**9** shows that this modification results in a dramatic decrease in alkaline stability. This appears to be due to a combination of the inductive-withdrawing *N*-Ph fragment destabilizing the quaternary ammonium cation and an enhancement in the rate of Hofmann elimination by increasing the acidity of the beta-hydrogen atoms. Finally, with the aim of quantifying the influence of the piperazinium amine fragment on stability, an alkaline decomposition study was conducted with **10**, as it bears a close structural similarity to **8** but with the amine nitrogen replaced by CH and, as such, was the most appropriate literature benchmark model head group. The half-life of 7,960 h, determined at 80 °C and λ = 4.8, is consistent with the previously reported value of 7,953 h^50–51^ and is nearly 3-fold higher than the corresponding half-lives determined for **8** and **9**, confirming that the *N*-heteroatom does have a dramatic effect on the stability of QA cations, in which it activates the piperazinium ring toward E2 elimination. As a further comparison with **8** and **9**, the degradation of **10** was also conducted at 60 °C, and the half-life of 18,785 h is markedly higher than the 14,363 and 14,453 h obtained with **8** and **9**, respectively (Figure S67). However, despite the improved stability of Bn-ASU (**10**), it would be synthetically challenging to introduce a polymerizable vinyl group into this cation, and as such, it is not a viable motif to develop individual monomers for the fabrication of polystyrene-based AEMs. This highlights the need to include the *N*-heteroatom as a tether point despite the notable effect on alkaline stability.

### 3.3 Computational Studies

DFT calculations were performed to establish the relative stability of model head groups **1** and **7** and to determine the activation energies for hydroxide-based nucleophilic dealkylative substitutions and E2 elimination, supporting the results of the experimentally observed degradation. The DFT functional B3LYP was selected to enable comparisons with a recent computational study that analyzed the degradation mechanisms of a series of heterocyclic QA cations.^62^ Calculations were conducted at 60 °C, as this temperature was initially used to study the stability of all the model head groups across a range of hydration numbers, exploring the influence of increasing solvation (λ = 0, 1, 2, 3, 4) on the reaction energies and activation energies for each of the possible degradation pathways for cations **1** and **7**. The results are summarized in Figure 5 (see Figures S78–S82 for the complete set of Gibbs free energy profiles at hydration levels λ = 0–4 and S71–S77 for the corresponding calculated TS structures). To this end, an increase in the solvation of hydroxide has previously been reported to slow the degradation of quaternary ammonium cations by reducing its nucleophilicity.^66,67^ A maximum hydration number of four was chosen based on a recent combined experimental and computational study that provided unequivocal evidence for the presence of four water molecules in the primary solvation shell of the hydroxide anion.^74^ This computational study was restricted to exploring degradation by nucleophilic attack of hydroxide due to the time-intensive nature of the calculations and because methoxide was observed to act as a nucleophile only in the ring-opening dealkylative degradation of **2**.

Interestingly, initial calculations conducted at λ = 0 were not consistent with the relative stabilities determined from the experimental degradations. Hofmann elimination was computed to have the lowest activation energy of all the potential degradation pathways for both **1** and **7**, with Δ*G* ^⧧^ values of 16.62 kcal mol^–1^ and 18.90 kcal mol^–1^, respectively, indicating that E2 should be the major degradation pathway at low hydration levels (Figure 5). These values were notably lower than the calculated activation energies for the observed degradation pathways, which were 20.08 kcal mol^–1^ for the demethylation of **1** and 22.15 kcal mol^–1^ for the debenzylation of **7**. As the hydration level of the hydroxide anion increased, there was an observed increase in all the activation energies for both head groups, as well as a change in ordering. At hydration level λ = 4, computed conditions that most closely resemble those used for the experimental degradation conducted at λ = 4.8, the lowest activation energy pathways are demethylation for **1** (32.28 kcal mol^–1^) and debenzylation for **7** (36.60 kcal mol^–1^). These values are in line with the experimentally observed degradations, as dealkylation was identified as the only pathway for both cations, and moreover, the values are consistent with the relative stability of **1** and **7**, as the former has a much shorter half-life than the latter. For comparison, the activation energies for E2 elimination at hydration level λ = 4 increased to 35.33 kcal mol^–1^ and 38.35 kcal mol^–1^ for **1** and **7**, respectively. The Gibbs free energy profiles for the various S_N_2 dealkylations and E2 elimination pathways for cations **1** and **7** calculated at hydration levels of 0 and 4 are shown in Figure 6a,b, and the corresponding profiles calculated under λ = 1, 2, and 3 conditions are presented in Figures S79–S81. Figure 5 also shows that the activation energies for each of the three degradation pathways for *N*-phenyl piperidinium **1** are significantly lower at each hydration level than the corresponding activation energy for the analogous *N*-benzyl piperidinium head group **7**.

These calculations also indicate that of the competing S_N_2 pathways, those involving ring-opening dealkylation have consistently higher activation barriers than those for either demethylation or debenzylation across all hydration numbers (λ = 0–4), which is in keeping with the experimental degradation studies, as there was no evidence for the formation of ring-opened alcohol degradation products.

The computed activation energies in Figures 5 and 6 also confirm the dramatic effect of hydration on head group degradation, which has been attributed to the nucleophilicity of the HO^–^(H_2_O)_λ_ cluster anion.^71^ As the hydration level increases, the hydroxide becomes more solvated and thereby less nucleophilic, with a larger effective size, which translates to an increase in the activation energy for nucleophilic attack.^75,76^ These computed free energies have also shown that the hydration level influences the major degradation pathway, as changes in the activation energy are hydration-dependent; i.e., E2 is favored where the hydroxide is least strongly solvated and hence has the highest charge density and thereby the greatest basicity. The change in the degradation pathway with increasing hydration suggests that the number of solvating water molecules has a greater impact on the transition state for E2 elimination than on that for S_N_2-based demethylation and debenzylation. This difference may be a result of the steric bulk associated with the first hydration shell of the hydroxide at λ = 4, limiting its approach to the acidic beta hydrogen atom in the transition state for E2 elimination more than its approach to the methyl/benzyl group in the transition state for S_N_2 dealkylation (Figure 7a,b, respectively). Using **1** as an example, the former requires a much shorter HO---H distance of 1.21 Å in the TS compared with a HO---CH_3_ distance of 2.05 Å for the latter. Moreover, the hydrogen-bonded solvation sphere must be disrupted prior to nucleophilic attack, and the extent of this disruption may well be mechanism-dependent.

Finally, while it is worthwhile to consider stepwise deprotonation of a β–C–H to generate the corresponding conjugate base, followed by ring-opening nitrogen–carbon bond cleavage as the C–C double bond forms and the amine leaves, i.e., an E1cB mechanism, we do not believe this to be a credible pathway. The β–C–H bonds in **1** and **7** are adjacent to a methylene fragment and are unlikely to be acidic; moreover, these protons will have similar p*K*_a_s. Preliminary investigative calculations on model head group **1** suggest that this pathway is unfavorable compared to E2 elimination, as the energy of the optimized structure for the conjugate base was disproportionately high compared to the energy of the E2 transition state. Moreover, the conjugate base optimized to adopt the same geometry as the alkene E2 elimination product, confirming that degradation of piperidinium cations via E1cB elimination is highly unlikely, even in the presence of a strong electron-withdrawing phenyl substituent, as in the case of **1**. However, for the piperazinium-based head groups **5**–**6** and **8**–**9**, the β–C-H atoms that would be involved in the E1cB elimination are adjacent to either *N*-Ph (**5** and **8**) or *N*-Bn (**6** and **9**). These fragments may influence the acidity of adjacent protons more than the methylene fragment influences the adjacent β-protons in **1** and **7**. Further computational studies will be required to thoroughly explore each possible degradation pathway for the piperazinium-based head groups to develop an understanding of how the amine in the ring influences stability and degradation.

The Gibbs free energy changes also reveal that degradations using the bare hydroxide nucleophile (i.e., λ = 0) are exothermic, with Δ*G*_r_ values between −16.44 and −42.51 kcal mol^–1^, consistent with previously reported reaction energies.^77^ As the hydration level increases, the reaction becomes progressively less exothermic, although even at a hydration level of 4, the overall reaction remains exothermic, with Δ*G*_r_ values between −4.66 and −22.04 kcal mol^–1^. The only exception is for S_N_2 ring-opening of **7**, which is the only degradation pathway to be endothermic, with a Δ*G*_r_ value of 3.72 kcal mol^–1^ at λ = 4. In contrast, debenzylation of the trimethylbenzylammonium cation has been reported to become endothermic for λ = 3, reaching 8.81 kcal mol^–1^ for λ = 4.^66^

The change in the degradation mechanism for quaternary ammonium cations as a function of the hydration number highlights the importance of considering all degradation pathways in the design of model head groups, as well as their modification for use as monomers to prepare AEM-based solid polymer electrolytes with sufficient chemical and mechanical stability. This is particularly relevant when designing membranes for electrolyzer or fuel cell applications, as each device experiences different hydration levels and might require different supporting electrolytes (e.g., a WE requires electrolyte pH 13–14). Thus, a selected head group for one device may not be suitable for application in the other.

## 4 Conclusions

A series of *N*-phenyl-substituted quaternary ammonium and piperazinium cationic head groups have been prepared, and their alkaline stability has been investigated and compared with their *N*-benzyl counterparts on the basis that the *N*-Ph fragment can be constructed via Buchwald–Hartwig amination and, as such, is a potentially versatile linker to attach a head group to a polymer. While substitution of the *N*-benzyl fragment with *N*-phenyl was expected to improve the alkaline stability of the derived cations by eliminating the S_N_2 debenzylation pathway, it appears to enhance the rate of E2 elimination, and as such, *N*-phenyl-based quaternary ammonium cations are much less stable than their *N*-benzylammonium counterparts. The stability profiles of *N*-phenyl and *N*-benzyl-based piperazinium model head groups were also explored, as the tertiary amine is a potential site of attachment to a polymer. While the *N*-phenyl piperazinium head groups were markedly more stable than *N*-Ph quaternary ammonium cations, they were much less stable than the corresponding *N*-benzyl piperazinium cations, as the *N*-Ph fragment activates the beta-hydrogen atoms toward E2 elimination. The half-life of 7,960 h determined for the benchmark cation **10** at 80 °C and λ = 4.8 is nearly 3-fold higher than the corresponding half-life determined for its *N*-heteroatom-tethered analogue, confirming that the *N*-heteroatom does have a dramatic effect on the stability of QA cations in that it activates the piperazinium ring toward E2 elimination. However, despite the improved stability of **10**, it would be synthetically challenging to introduce a polymerizable vinyl group into this cation, and as such, it is not a viable motif to develop monomers for the fabrication of polystyrene-based AEMs. We are now utilizing our developed stability–structure descriptor to inform the design of more stable amino-modified head groups to identify alkaline-stable and durable anion-exchange polyelectrolytes for use in fuel cells and electrolyzers toward green energy applications. To this end, we are currently exploring the influence of the tether length and location of the amine on alkaline stability and have developed a series of model amino-modified head groups with impressive stability profiles. Polyelectrolytes based on the most stable of these monomers are currently being prepared, and their performance as ionomers and AEMs in a water electrolyzer is being investigated, full details of which will be disclosed in future publications.

## Supplementary Material



## Notes

Synthesis and characterization of model head groups **1–9**, kinetic plots for alkaline degradation studies on head groups **1–9**, NMR data and mass spectra for degradation mixtures, and degradation product assignment; details of calculated Gibbs free energies for model head groups **1** and **7**, TS structures for the various S_N_2 dealkylative substitution, ring-opening dealkylation, and E2 degradation pathways for **1** and **7** and Gibbs free energy profiles of the S_N_2 dealkylative substitution, ring-opening dealkylation, and E2 degradation pathways for head groups **1** and **7**, as a function of hydration number; CCDC 2425253-2425257, 2426576, and 2426577 contain the supplementary crystallographic data for this paper; these data can be obtained free of charge via www.ccdc.cam.ac.uk/data_request/cif, or by emailing data_request@ccdc.cam.ac.uk, or by contacting The Cambridge Crystallographic Data Centre, 12 Union Road, Cambridge CB2 1EZ, UK; fax: + 44 1223 336033 (PDF)
