# Supplementary material for: Structure–Stability Correlations on Quaternary Ammonium Cations as Model Monomers for Anion-Exchange Membranes and Ionomers
Source: ACS Appl Energy Mater. 2025 Jun 28;8(13):9718–30. doi: 10.1021/acsaem.5c01293 (PMC12264860; doi:10.1021/acsaem.5c01293)
Supplement: Supplementary file 1 [file ae5c01293_si_001.pdf]

Supporting Information

Structure-Stability Correlations on Quaternary Ammonium Cations as Model Monomers for Anion Exchange Membranes and Ionomers

Samuel D. T. Power[a], Corinne Wills[a], Casey M. Dixon[a], Paul G. Waddell[a], Julian G. Knight[a], Mohamed Mamlouk[b],* and Simon Doherty[a],*

[a] Newcastle University Centre for Catalysis (NUCAT), School of Chemistry, Newcastle University, Bedson Building, Newcastle upon Tyne NE1 7RU, UK

[b] School of Engineering, Merz Court, Newcastle University, Newcastle upon Tyne NE17RU, UK

* Email: simon.doherty@newcastle.ac.uk,

* Email: mohamed.mamlouk@newcastle.ac.uk

Contents

Synthesis and characterization of Model Head Groups **1-9**...S3-S19

X-ray Crystallography...S20-S35

Headgroup Alkaline Stability Studies on Model Head Groups 1-9................................S36-S65

Computational study of head groups **1** and **7**..S66-S77

Synthesis and characterization of Model Head Groups 1-9

General Comments

Methods and Instrumentation. All ^{1}H and ^{13}C{^{1}H} NMR spectra were recorded either in $CDCl_3$ or CD_3OD on a Bruker Avance III 300 MHz, Bruker Avance NEO 400 MHz, Bruker Avance III HD 500 MHz or a Bruker Avance III HD 700 MHz NMR spectrometer at 25 °C; chemical shifts are reported relative to the residual solvent peak ($CDCl_3$ or CD_3OD) and are quoted in ppm relative to tetramethylsilane. Electrospray ionization mass spectrometry (ESI-MS) analyses were performed on an Agilent MSD-XT instrument.

Chemicals. 3-(Trimethylsilyl)-1-propanesulfonic acid sodium salt, methanol-d_4, NaOD 40 wt. % in D_2O, 4-benzylpiperidine (Sigma-Aldrich), palladium(II) acetate (Merck), piperidine (Merck), XPHOS (Fluorochem), D_2O, sodium *tert*-butoxide, piperazine, *N*-methyl piperazine, pyrrolidine, morpholine, *N,N*-dimethylaniline, bromobenzene, benzyl bromide, 1,5-dibromopentane and methyl iodide (Thermo Fischer Scientific) were all used as received.

Experimental

Synthesis of *N*-phenyl-based tertiary amines (1b, 2b, 3b, 5b and 6b). To an oven dried Schlenk flask under N_2 was added amine (25 mmol, 1.0 eq), bromobenzene (3.14 g, 20 mmol, 1.2 eq), NaO^{t}Bu (2.88 g, 30 mmol, 1.5 eq), $Pd(OAc)_2$ (0.045 g, 0.2 mmol, 0.01 eq) and XPhos (0.142 g, 0.3 mmol, 0.015 eq) and dry toluene (30 mL). The resulting mixture was heated to 90 °C and stirred for 24 h, after which time the solution was allowed to cool to room temperature, diluted with ethyl acetate (30 mL) and filtered. The filtrate was washed with water (3 x 50 mL), brine (50 mL) and $NaHCO_3$ (50 mL), before the organic phase was dried over $MgSO_4$ and the volatiles removed under reduced pressure to afford the desired tertiary amines **1b**, **2b**, **3b**, **5b** and **6b**, which were used without further purification. Recorded ^{1}H NMR spectra were consistent with previous literature reports.[1,2] **1b:** ^{1}H NMR (400 MHz, $CDCl_3$, δ): 7.27-7.22 (m, 2H), 6.95 (m, 2H), 6.82 (m 1H), 3.15 (t, 4H), 1.71 (m, 4H), 1.58 (m, 2H). **2b:** ^{1}H NMR (400 MHz, $CDCl_3$, δ): 7.30-7.25 (m, 2H), 6.70 (m, 1H), 6.62 (m, 2H), 3.33 (m, 4H), 2.05 (m,

4H). **3b:** ^{1}H NMR (400 MHz, $CDCl_3$, *δ*): 7.33-7.26 (m, 2H), 6.94-6.85 (m, 3H), 3.87 (t, 4H), 3.16 (t, 4H). **5b:** ^{1}H NMR (400 MHz, $CDCl_3$, *δ*): δ 7.32-7.24 (M, 2H), 6.95-6.92 (m, 2H), 6.88-6.84 (m, 1H), 3.15 (m, 4H), 3.04 (m, 4H). **6b:** ^{1}H NMR (300 MHz, $CDCl_3$, *δ*): δ 7.32-7.21 (m, 2H), 6.94 (d, 2H), 6.87 (t, 1H), 3.27 (m, 4H), 2.66 (m, 4H), 2.41 (s, 3H).

Synthesis of *N*-benzylpiperidine (7b). An oven dried Schlenk flask under N_2 was charged with benzyl bromide (6.91 g, 40.4 mmol, 1.0 eq) and ethyl acetate (80 mL) before cooling to 0 °C, after which piperidine (6.88 g, 80.8 mmol, 2.0 eq) was added dropwise. The resulting solution was warmed to room temperature and stirred for 1 h. After this time the reaction mixture was diluted by addition of ethyl acetate (100 mL) before washing with water (3 x 100 mL) and brine (100 mL). The organic phase was dried over $MgSO_4$ and the volatiles removed under reduced pressure to afford *N*-benzylpiperidine (**7b**) as a colorless oil (6.38 g, 89%).[3] ^{1}H NMR (400 MHz, $CDCl_3$, *δ*): 7.25-7.13 (m, 5H), 3.40 (s, 2H), 2.30 (m, 4H), 1.50 (q, 4H), 1.35 (m, 2H).

Synthesis of *N*-benzylpiperazine (8b). To a two-necked-flask under nitrogen was added piperazine (17.24 g, 200 mmol, 5 eq) and dry dichloromethane (100 mL). The solution was cooled to 0 °C, after which benzyl bromide (6.84 g, 40 mmol, 1.0 eq) was added dropwise and the reaction mixture was stirred at 0 °C for 1 h. After this time the reaction mixture was washed with water (2 x 100 mL) and $NaHCO_3$ (2 x 100 mL). The organic phase was dried over $MgSO_4$ and the volatiles removed under reduced pressure to afford *N*-benzyl-piperazine as a colourless oil (4.05 g, 57 %).[4] ^{1}H NMR (400 MHz, $CDCl_3$, *δ*): 7.34-7.28 (m, 4H), 7.26-7.21 (m, 1H), 3.47 (s, 2H), 2.88 (t, 4H), 2.54-2.36 (m, 4H).

Synthesis of *N*-benzyl-*N'*-methylpiperazine (9b). An oven dried Schlenk under N_2 was charged with K_2CO_3 (1.66 g, 12 mmol, 1.2 eq), *N'*-methylpiperazine (1.26 g, 12.62 mmol, 1.2 eq) and dry dichloromethane (30 mL). Benzyl bromide (1.723 g, 10.10 mmol, 1.0 eq) was added dropwise and the solution was stirred at room temperature for 24 h, after which time the

volatiles were removed under reduced pressure. The residue was diluted with water (100 mL) and then extracted with ethyl acetate (3 x 50 mL). The organic phases were collected, washed with brine (50 mL), dried using $MgSO_4$ and the volatiles removed under vacuum to afford *N*-benzyl-*N'*-methylpiperazine as a colorless oil (0.90 g, 4.74 mmol, 47 %).[5] ^{1}H NMR (400 MHz, $CDCl_3$, δ): 7.33-7.27 (m, 4H), 7.27-7.22 (m, 1H), 3.51 (s, 2H), 2.59-2.31 (m, 8H), 2.28 (s, 3H).

Synthesis of 1-methyl-1-phenylpiperidinium iodide (1), 1-methyl-1-phenylpyrrolidinium iodide (2), 1-methyl-1-phenylmorpholinium iodide (3) and benzyltrimethyl ammonum iodide (4). A round-bottomed flask was charged with amine (20 mmol, 1.0 eq) and dichloromethane (25 mL) and cooled to 0 °C before adding MeI (4.26 g, 30 mmol, 1.5 eq) dropwise. The reaction mixture was allowed to warm to room temperature, flushed with N_2 and stirred for 48 hours. The reaction mixture was pipetted into ether causing precipitation. The precipitate was collected by filtration, washed with diethyl ether (3 x 20 mL) before being dissolved in water (50 mL). The aqueous phase was washed with dichloromethane (3 x 25 mL) and the water removed under reduced pressure to give *N*-phenyl quaternary ammonium salts **1-4** as spectroscopically pure products. **1:** ^{1}H NMR (400 MHz, $CDCl_3$, δ): 7.91 (d, 2H), 7.59 (t, 2H), 7.47 (t, 1H), 4.58 (m, 2H), 4.41 (m 2H), 3.68 (s, 3H)2.00-1.64 (m, 6H). $^{13}C\{^1H\}$ NMR (101 MHz, $CDCl_3$, δ): 142.51, 131.16, 130.31, 121.55, 63.11, 59.99, 21.27, 20.72. LCMS (ESI): $[M]^+$ = 176.1. **2:** ^{1}H NMR (400 MHz, $CDCl_3$, δ): 7.90 (d, 2H), 7.55-7.44 (m, 3H), 4.69 (m, 2H), 4.06 (m, 2H), 3.64 (s, 3H), 2.48-2.24 (m, 4H). $^{13}C\{^1H\}$ NMR (101 MHz, $CDCl_3$, δ): 145.69, 130.28, 130.14, 120.83, 66.20, 55.25, 20.67. LCMS (ESI): $[M]^+$ = 162.1. **3:** ^{1}H NMR (300 MHz, CD_3OD, δ): 7.95 (m, 2H), 7.75 (m, 2H), 7.67 (m, 1H), 4.49 (m, 2H), 4.18-4.09 (m, 4H), 3.90-3.84 (m, 2H), 3.62 (s, 3H). $^{13}C\{^1H\}$ NMR (75 MHz, CD_3OD, δ): 142.78, 131.02, 130.30, 121.59, 61.72,61.69, 58.91. LCMS (ESI): $[M]^+$ = 178.1. **4:** ^{1}H NMR (300 MHz, $CDCl_3$, δ): 7.93 (m, 2H), 7.68-7.54 (m, 3H), 4.04 (s, 9H).$^{13}C\{^1H\}$ NMR (75 MHz, $CDCl_3$, δ): 148.52, 131.63, 131.60, 121.21, 57.98. LCMS (ESI): $[M]^+$ = 136.1.

Synthesis of 1,1-dimethyl-4-phenylpiperazin-1-ium iodide (6). A round-bottomed flask was charged with *N*-phenylpiperazine (1.00 g, 5.67 mmol, 1.0 eq) and dichloromethane (10 mL) and cooled to 0 °C before adding MeI (0.64 g, 4.54 mmol, 0.8 eq) dropwise with rapid stirring. The solution was warmed to room temperature, flushed with N_2 and stirred for 48 h after which time the volatiles were removed under reduced pressure. The residue was mixed with ether (50 mL) to form a suspension, which was separated by filtration and the collected solid was washed with ether (3 x 20 mL). The solid was dissolved in water (25 mL), then washed with dichloromethane (3 x 25 mL) and the aqueous phase collected and the water removed under reduced pressure to 1,1-dimethyl-4-phenylpiperazin-1-ium iodide as an off-white solid in 90% yield (1.614 g, 5.07 mmol). ^{1}H NMR (400 MHz, CD_3OD, δ): 7.30 (m, 2H), 7.06 (d, 2H), 6.95 (t, 1H), 3.65 (m, 4H), 3.56 (m, 4H), 3.28 (s, 6H).^{13}C{^{1}H} NMR (101 MHz, CD_3OD, δ): 150.90, 130.27, 122.30, 117.72, 62.84, 51.97, 44.56. LCMS (ESI): $[M]^+$ = 191.1.

Synthesis of *N*-benzyl-*N*-methylpiperidin-1-ium iodide (7). A nitrogen-filled round-bottomed flask was charged with *N*-benzylpiperidine (6.07 g, 34.63 mmol, 1.0 eq) and dry THF (50 mL) and cooled to 0 °C before adding MeI (7.75 g 54.6 mmol, 1.5 eq) dropwise with rapid stirring. The reaction mixture was warmed to room temperature and stirred for 48 h, during which time a white precipitate formed. The solution was filtered, and the precipitate washed with ether (3 x 50 mL) to collect *N*-benzyl-*N*-methylpiperidin-1-ium iodide as a free-flowing white solid in 89% yield (10.23 g, 32.25 mmol). ^{1}H NMR (300 MHz, $CDCl_3$, δ): 7.60 (dd, 2H), 7.36-7.25 (m,3H), 4.98 (s, 2H), 3.71 (m, 2H), 3.51 (m, 2H), 3.09 (s, 3H), 1.90-1.57 (m, 6H). ^{13}C{^{1}H} NMR (75 MHz, $CDCl_3$, δ): 133.15, 130.35, 128.79, 126.53, 66.95, 59.82, 45.81, 20.44, 19.94. LCMS (ESI): $[M]^+$ = 190.1.

Synthesis of 1,1-dimethyl-4-benzylpiperazin-1-ium iodide (9). A nitrogen-filled round-bottomed flask was charged with *N*-benzyl-*N′*-methylpiperazine (0.8 g, 4.2 mmol, 1.0 eq) and dichloromethane (20 mL) and cooled to 0 °C before adding MeI (0.48 g, 3.37 mmol, 0.8 eq)

dropwise. The flask was warmed to room temperature, flushed with N_2 and stirred for 48 h after which time the volatiles were removed under reduced pressure. The residue was triturated with ethanol (50 mL) resulting in suspension of a colorless solid which was collected by filtration and washed with diethyl ether (3 x 20 mL) to afford 1,1-dimethyl-4-benzylpiperazin-1-ium iodide as a colorless solid in 80 % yield (0.89 g, 2.67 mmol). ^{1}H NMR (400 MHz, CD_3OD, δ): 7.39-7.27 (m, 5H), 3.69 (s, 2H), 3.51 (t, 4H), 3.20 (s, 6H), 2.83 (m, 4H). ^{13}C{^{1}H} NMR (101 MHz, CD_3OD, δ): 138.33, 130.21, 129.53, 128.64, 63.07, 62.38, 47.40. LCMS (ESI): $[M]^+$ = 205.1.

Synthesis of 3-phenyl-3,6-diazaspiro[5.5]undecan-6-ium bromide (5), 3-benzyl-3,6-diazaspiro[5.5]undecan-6-ium bromide (8) and 3-benzyl-6-azaspiro[5.5]undecane-6-ium bromide (10). A two-necked round-bottomed flask was charged with 1,5-dibromopentane (0.69 g, 3.00 mmol, 1.0 eq), K_2CO_3 (0.50 g, 3.60 mmol, 1.2 eq) and MeCN (10 mL) and the mixture heated to reflux before adding amine (3.00 mmol, 1 eq) dissolved in MeCN (5 mL) dropwise through a septum. The septum was replaced with a stopper and the flask heated at reflux for 20 h. After this time the reaction mixture was cooled to room temperature and the volatiles removed under reduced pressure. The residue was triturated with EtOH (50 mL), filtered and the filtrate concentrated under reduced pressure before being pipetted dropwise into a large volume of diethyl ether with vigorous stirring. The precipitate was allowed to settle, the majority of the diethyl ether decanted, and the remaining solution filtered. The solid was washed with diethyl ether (3 x 20 mL) and dried to afford **5**, **8** and **10** as colorless free-flowing powders. **5:** ^{1}H NMR (400 MHz, CD_3OD, δ): 7.31 (m, 2H), 7.06 (d, 2H), 6.95 (t, 1H), 3.72 (t, 4H), 3.60 (t, 4H), 3.54 (t, 4H), 1.97 (m, 4H), 1.78 (q, 2H). ^{13}C{^{1}H} NMR (101 MHz, CD_3OD, δ): 150.94, 130.32, 122.21, 117.57, 60.58, 59.67, 43.77, 22.60, 20.37. LCMS (ESI): $[M]^+$ = 231.1. **8:** ^{1}H NMR (400 MHz, CD_3OD, δ): 7.37-7.27 (m, 5H), 3.67 (s, 2H), 3.54 (t, 4H), 3.50 (t, 4H), 2.80 (t, 4H), 1.91 (m, 4H), 1.73 (q, 2H). ^{13}C{^{1}H} NMR (101 MHz, CD_3OD, δ): 138.07,

130.30, 129.53, 128.70, 62.38, 59.74, 46.65, 22.52, 20.32. LCMS (ESI): $[M]^+$ = 245.1. **10:** ^{1}H NMR (400 MHz, CD_3OD, δ): 7.33-7.27 (m, 2H), 7.23-7.18 (m, 3H), 3.81-3.76 (m, 2H), 3.53 (t, 2H), 3.43 (t, 2H), 3.27-3.17 (m, 2H), 2.68 (d, 2H), 2.03-1.67 (m, 11H).

References

1. Desmarets, C.; Schneider, R.; Fort, Y. Nickel(0)/Dihydroimidazol-2-ylidene Complex Catalyzed Coupling of Aryl Chlorides and Amines. *J. Org. Chem.* **2002**, *67*, 3029–3036.

2. Kwong, F. Y.; Klapars, A.; Buchwald, S. L. Copper-Catalyzed Coupling of Alkylamines and Aryl Iodides: An Efficient System Even in an Air Atmosphere. *Org. Lett.* **2002**, *4*, 581–584.

3. Sperotto, E.; van Klink, G. P. M. de Vries, J. G.; van Koten, G. C–N Coupling of nitrogen nucleophiles with aryl and heteroaryl bromides using aminoarenethiolato–copper(I) (pre-)catalyst, *Tetrahedron* **2010**, *66*, 3478-3484.

4. Yu, Z.; Yu, Z.; Li, M.; Wang, K.; Gu, Y; Guo, S.; Wang, W.; Ma, Y.; Liu, H.; Chen, Y. Novel Hybrids of 3-Substituted Coumarin and Phenylsulfonylfuroxan as Potent Antitumor Agents with Collateral Sensitivity against MCF-7/ADR. *J. Med. Chem.* **2022**, *65*, 13, 9328–9349.

5. Cui, X.; Dai, X.; Deng, Y.; Shi, F. Development of a General Non-Noble Metal Catalyst for the Benign Amination of Alcohols with Amines and Ammonia. *Chem. Eur. J.* **2013**, *19*, 3665–3675.

NMR Spectra for Model Headgroups 1-9

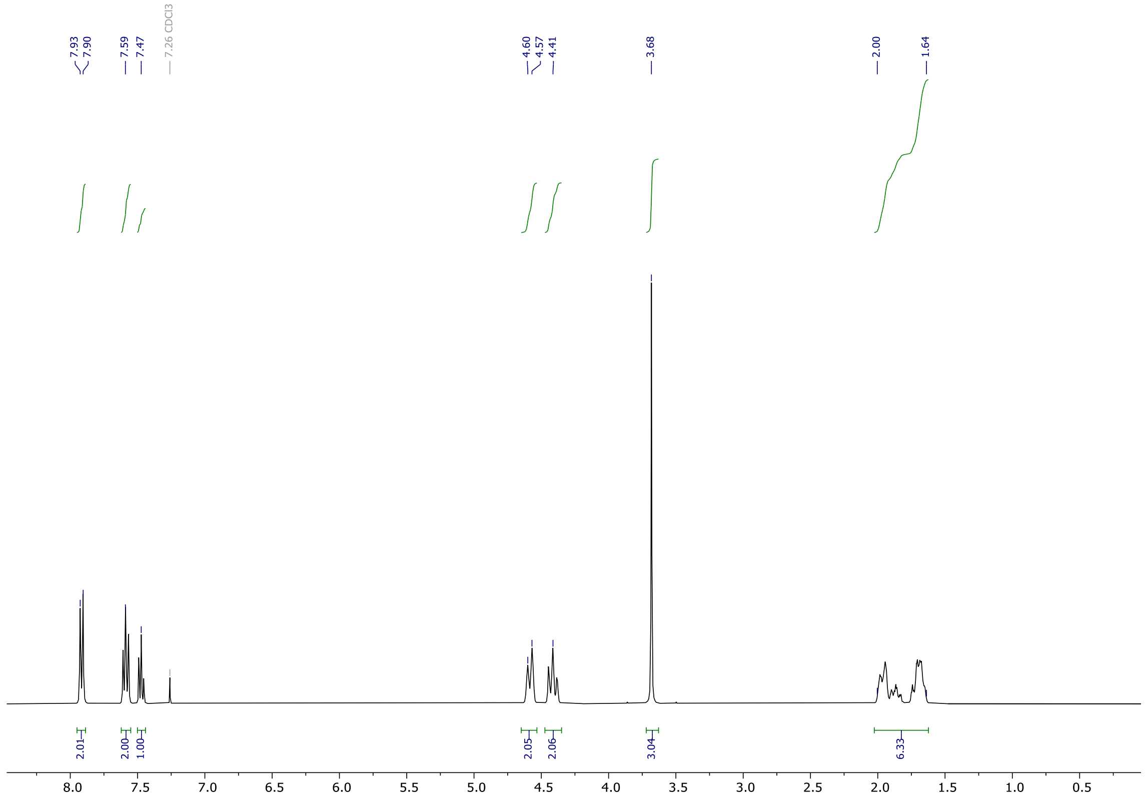

Figure S1: ^{1}H NMR spectrum (400 MHz) of **1** recorded in $CDCl_3$.

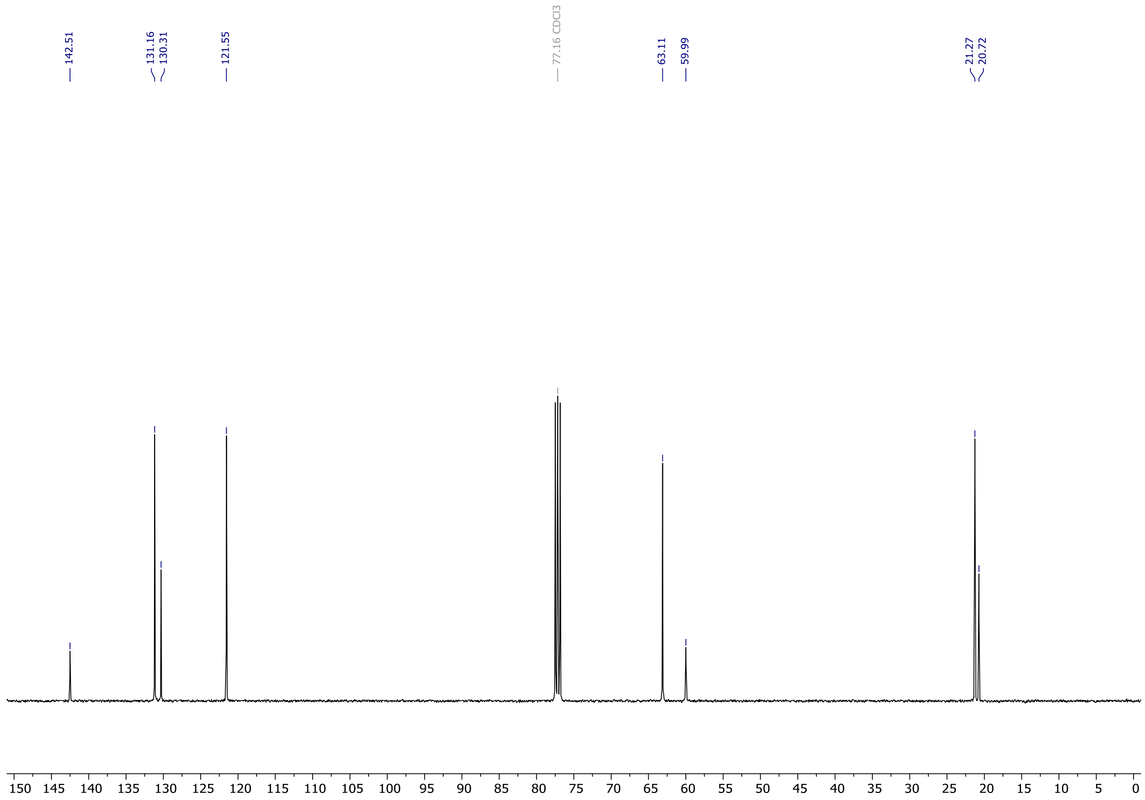

Figure S2: $^{13}C\{^{1}H\}$ NMR spectrum (75 MHz) of **1** recorded in $CDCl_3$.

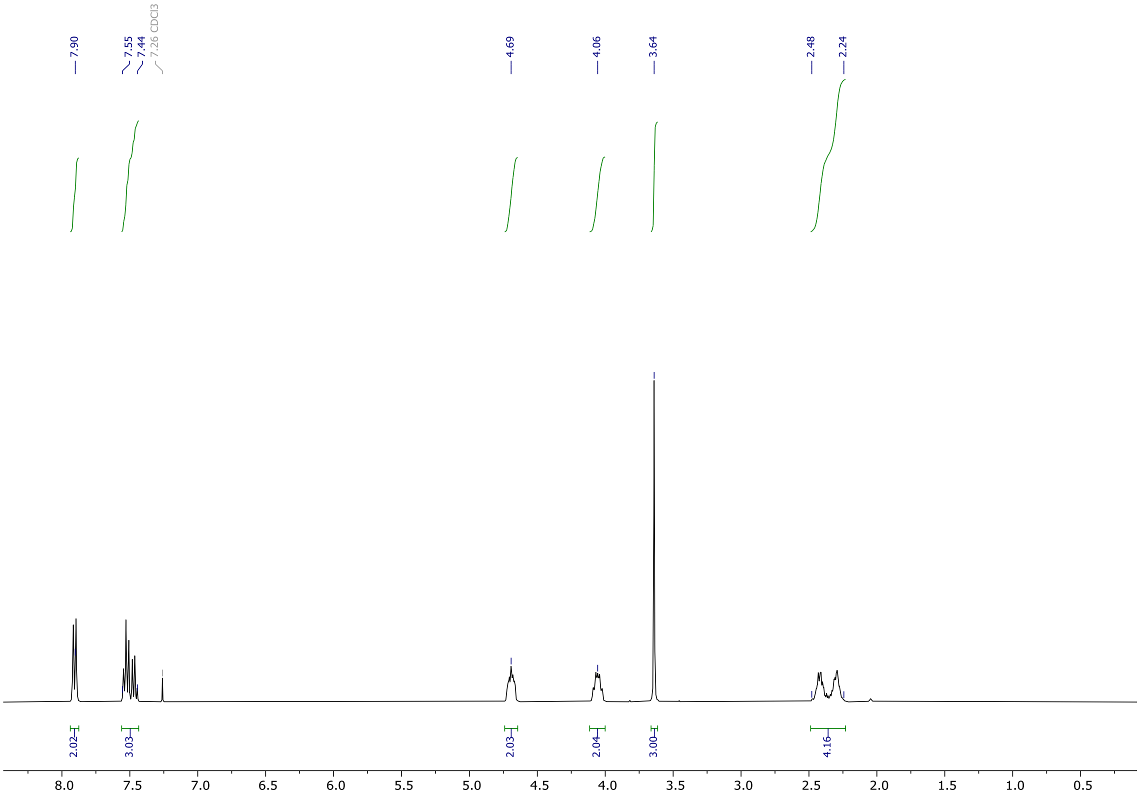

Figure S3: ^{1}H NMR spectrum (400 MHz) of **2** recorded in $CDCl_3$.

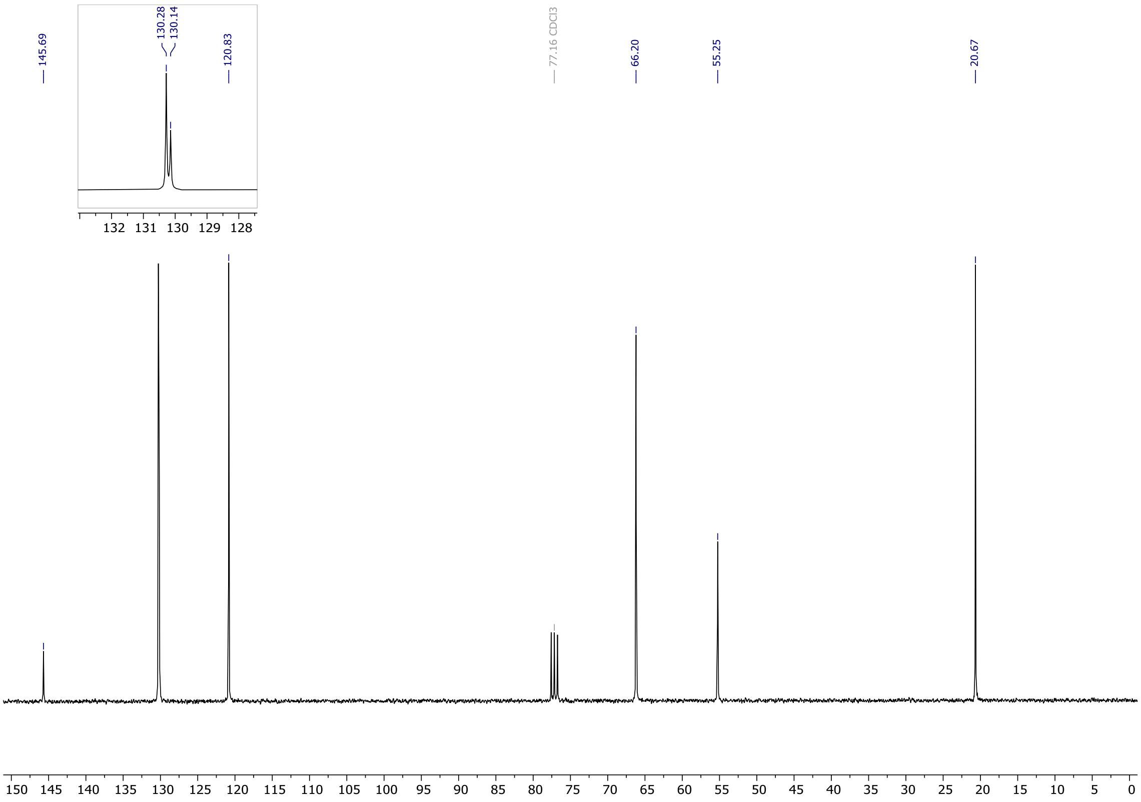

Figure S4: $^{13}C\{^{1}H\}$ NMR spectrum (75 MHz) of **2** recorded in $CDCl_3$.

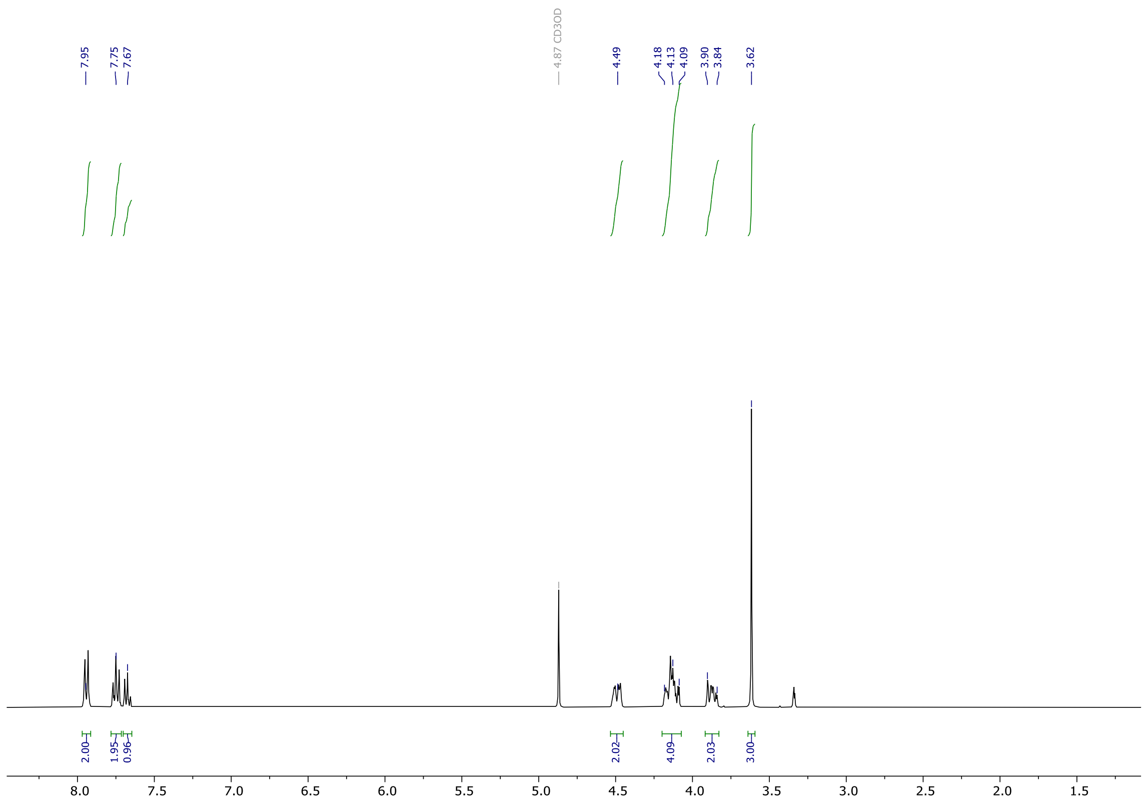

Figure S5: ^{1}H NMR spectrum (400 MHz) of **3** recorded in CD_3OD.

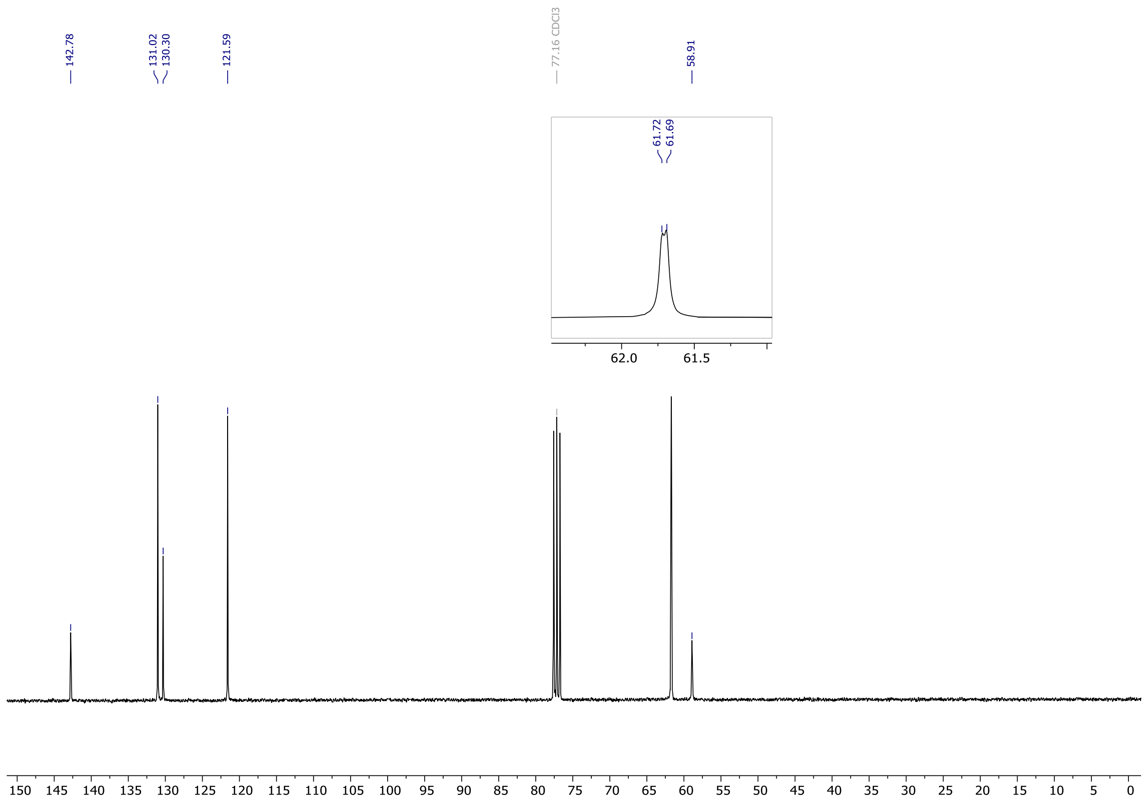

Figure S6: $^{13}C\{^{1}H\}$ NMR spectrum (75 MHz) of **3** recorded in $CDCl_3$

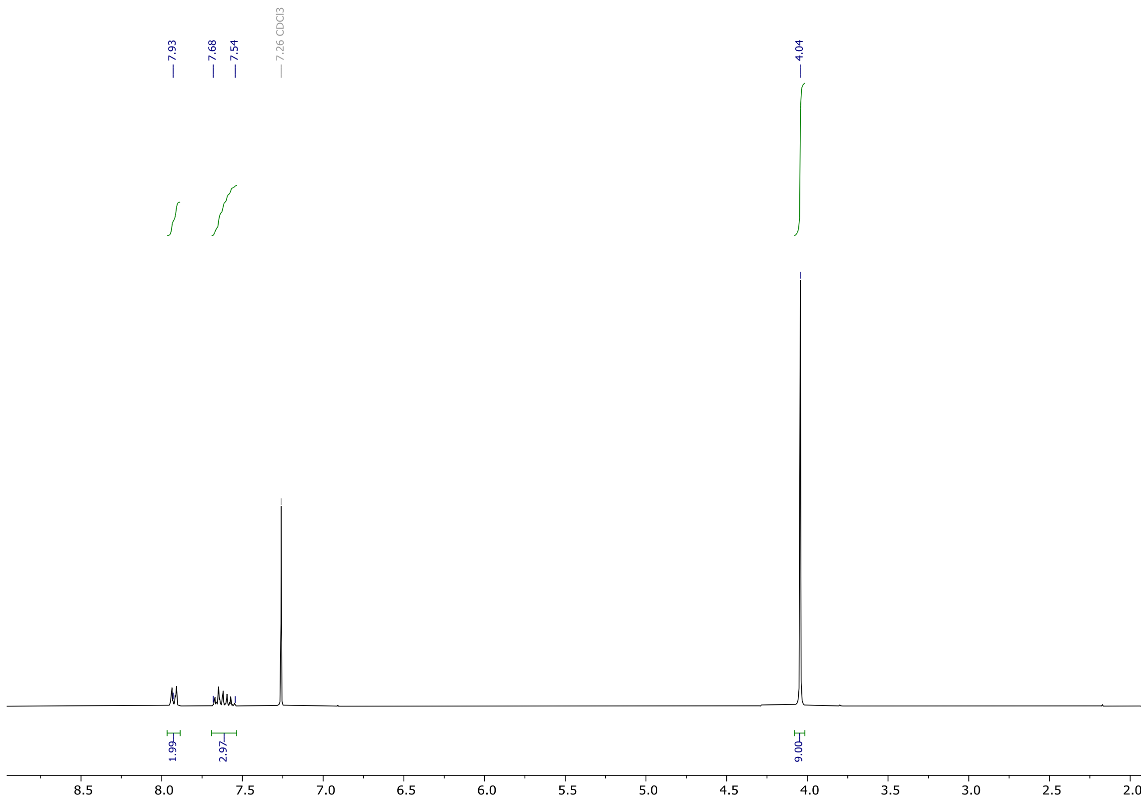

Figure S7: ^{1}H NMR spectrum (400 MHz) of **4** recorded in $CDCl_3$.

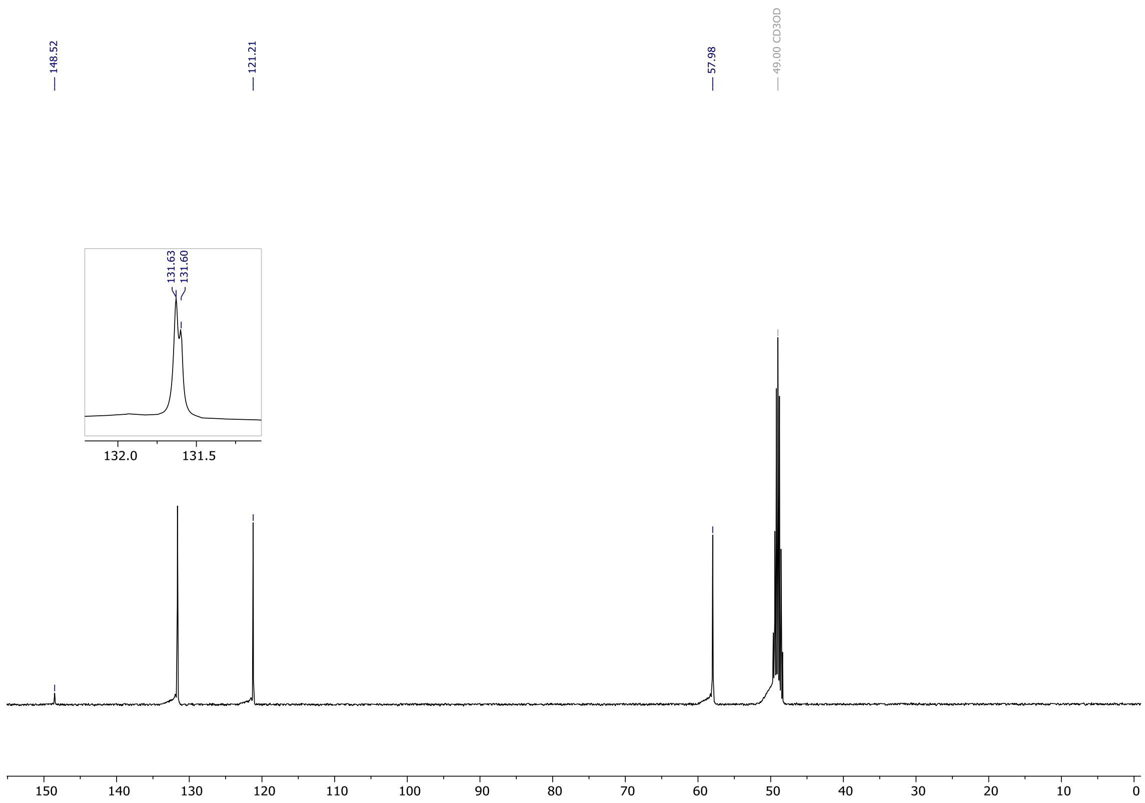

Figure S8: $^{13}C\{^{1}H\}$ NMR spectrum (101 MHz) of **4** recorded in CD_3OD.

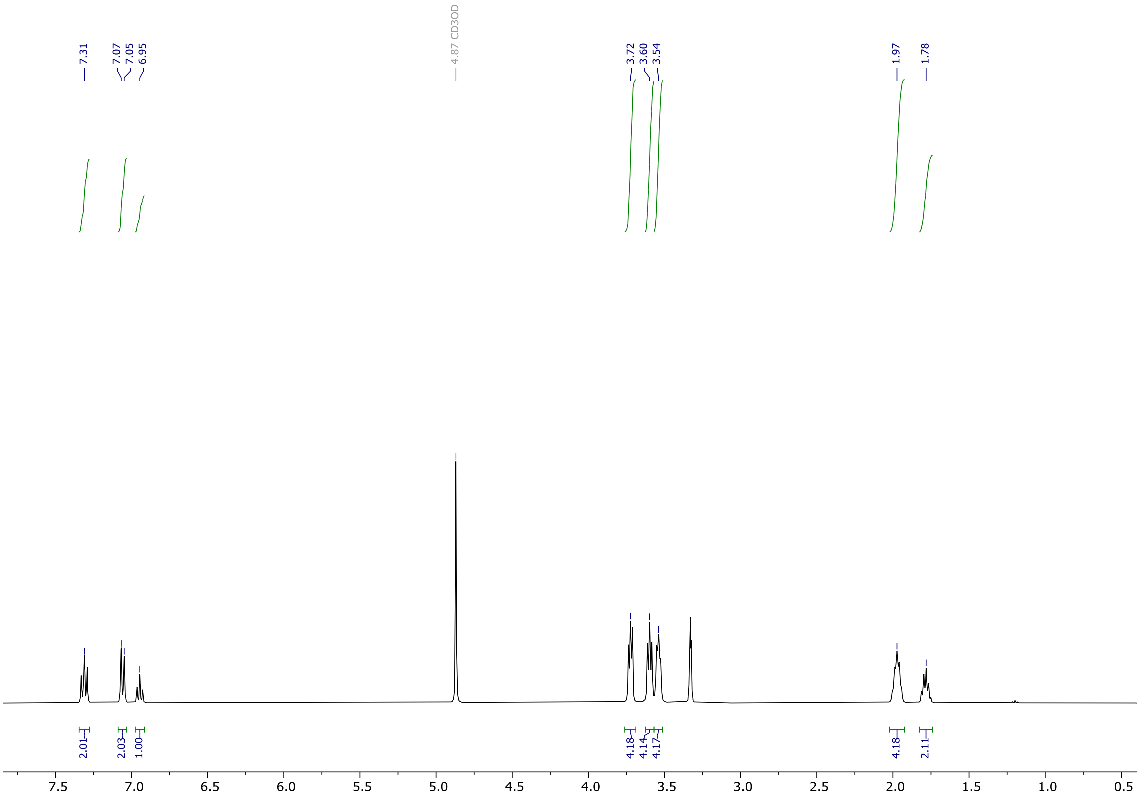

Figure S9: ^{1}H NMR spectrum (400 MHz) of **5** recorded in CD_3OD.

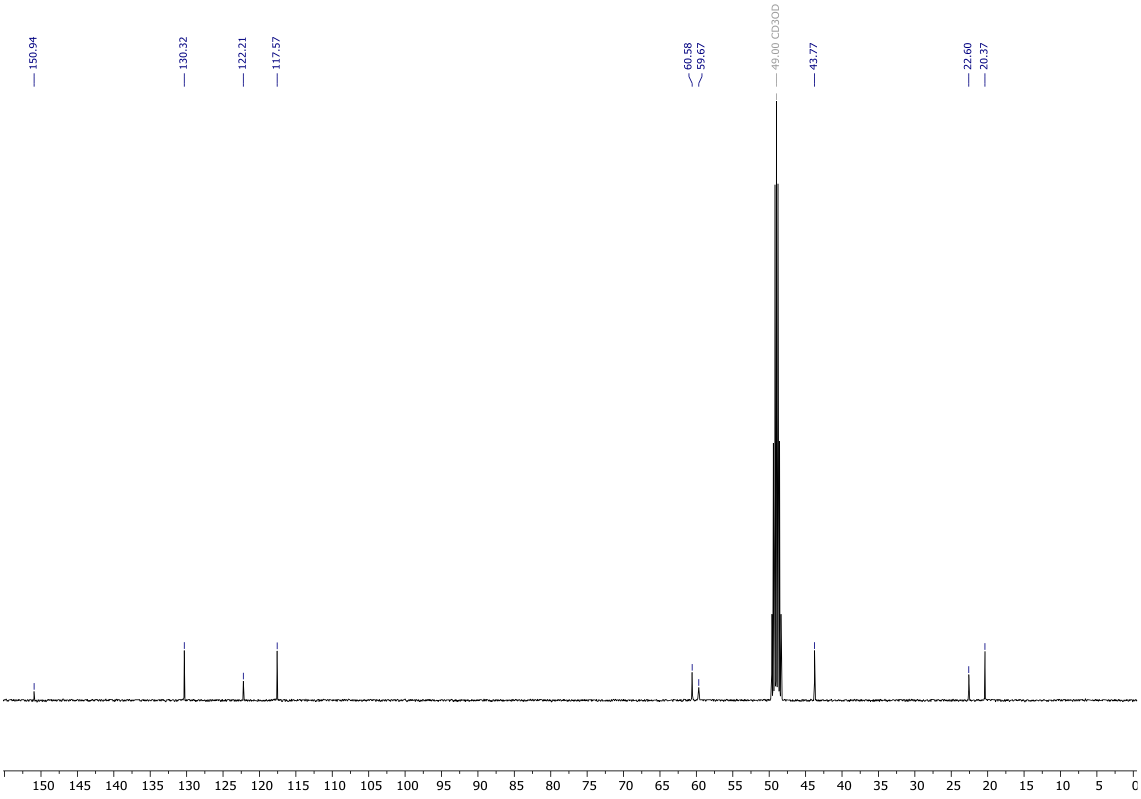

Figure S10: $^{13}C\{^{1}H\}$ NMR spectrum (101 MHz) of **5** recorded in CD_3OD.

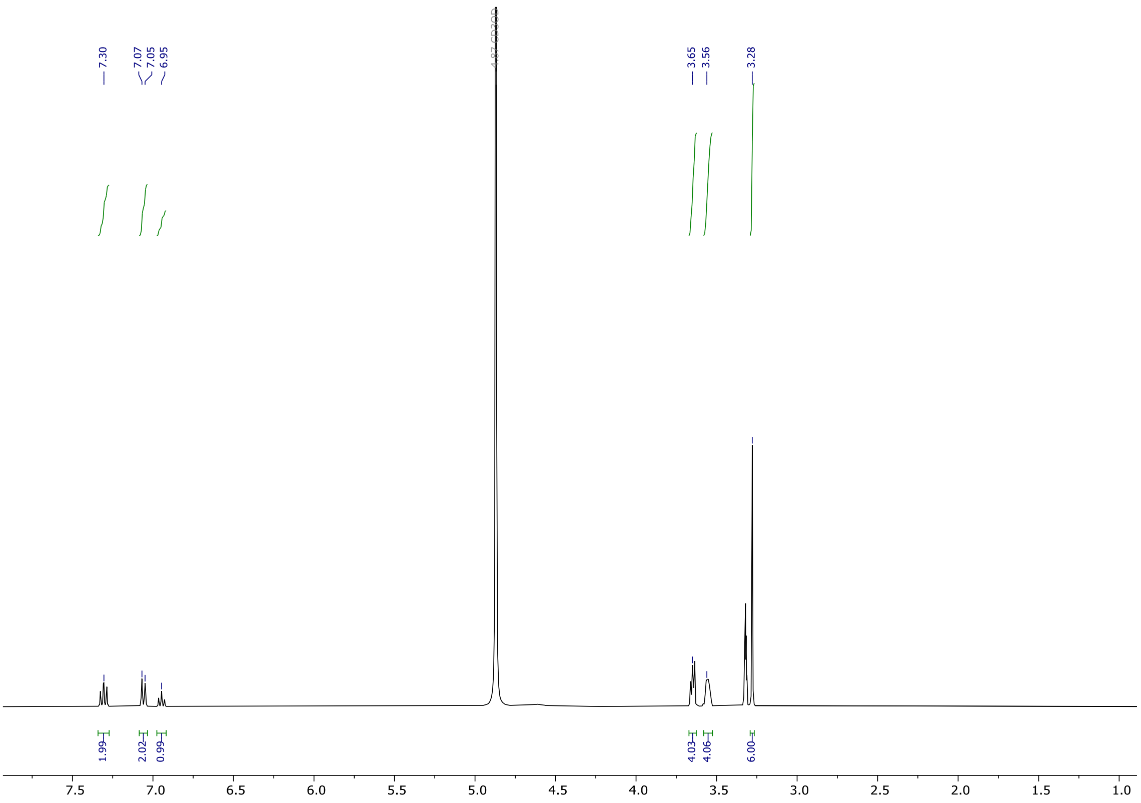

Figure S11: ^{1}H NMR spectrum (400 MHz) of **6** recorded in CD_3OD.

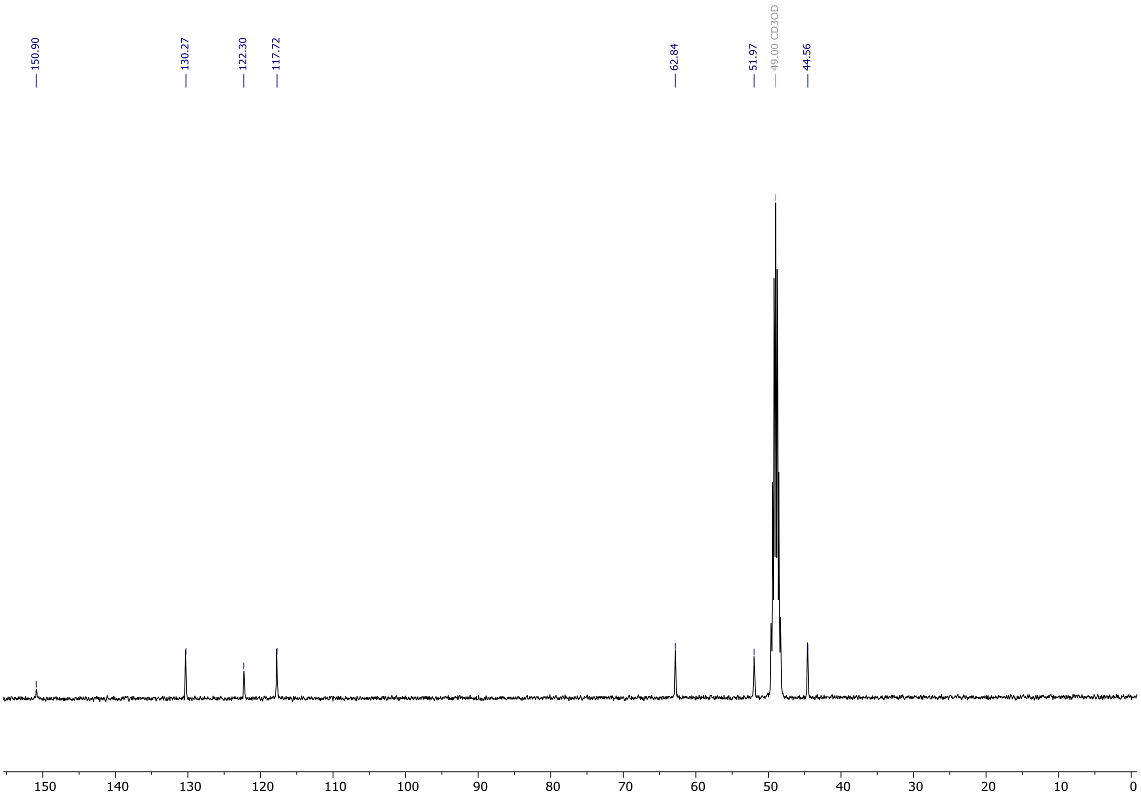

Figure S12: $^{13}C\{^{1}H\}$ NMR spectrum (101 MHz) of **6** recorded in CD_3OD.

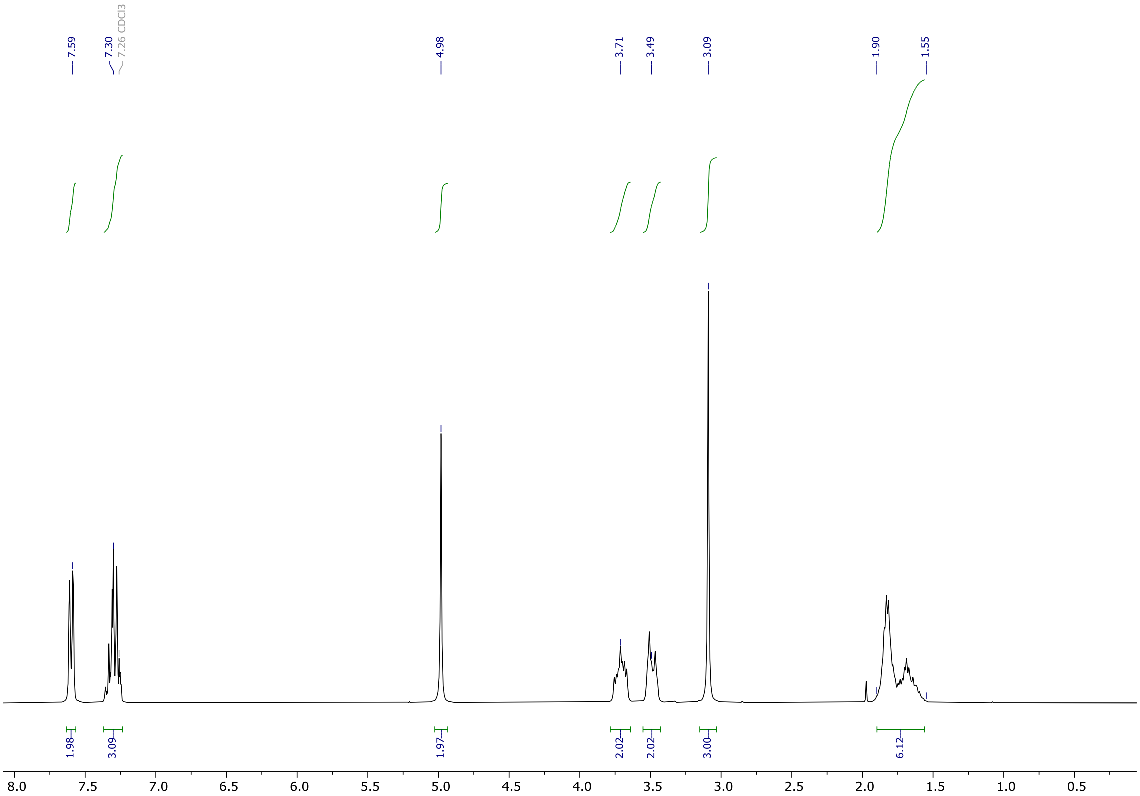

Figure S13: ^{1}H NMR spectrum (300 MHz) of **7** recorded in $CDCl_3$

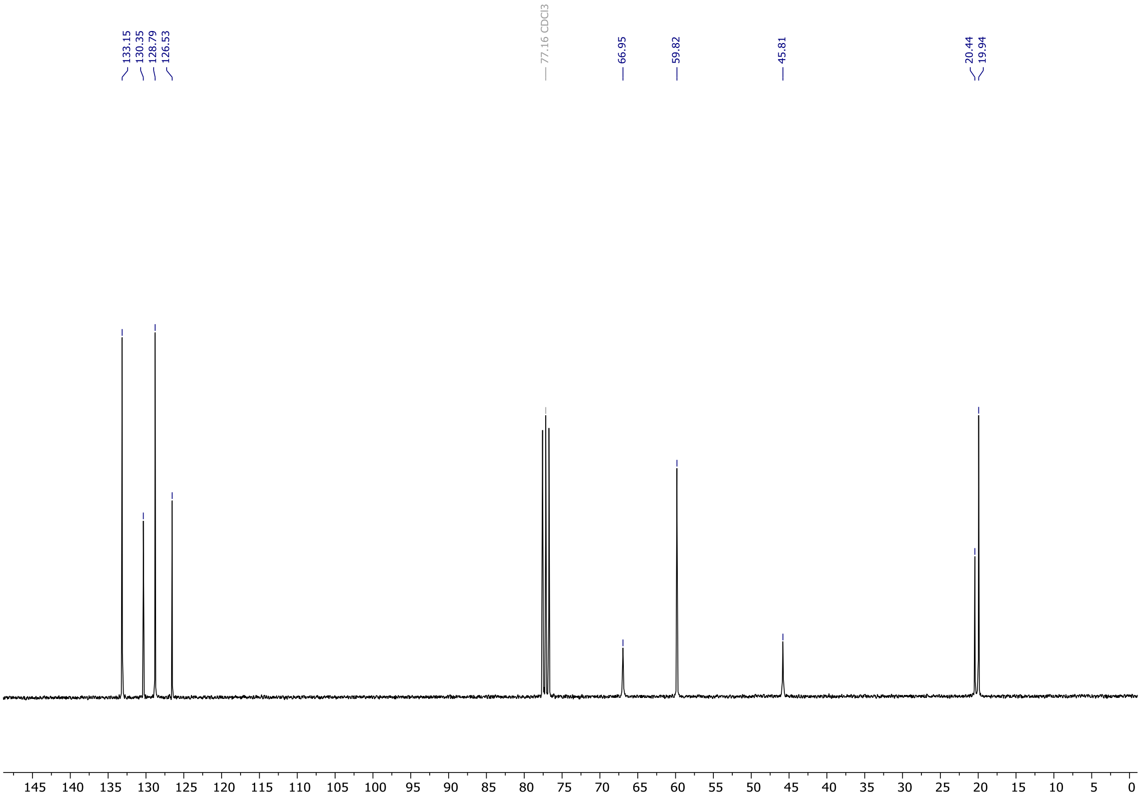

Figure S14: $^{13}C\{^{1}H\}$ NMR spectrum (75 MHz) of **7** recorded in $CDCl_3$.

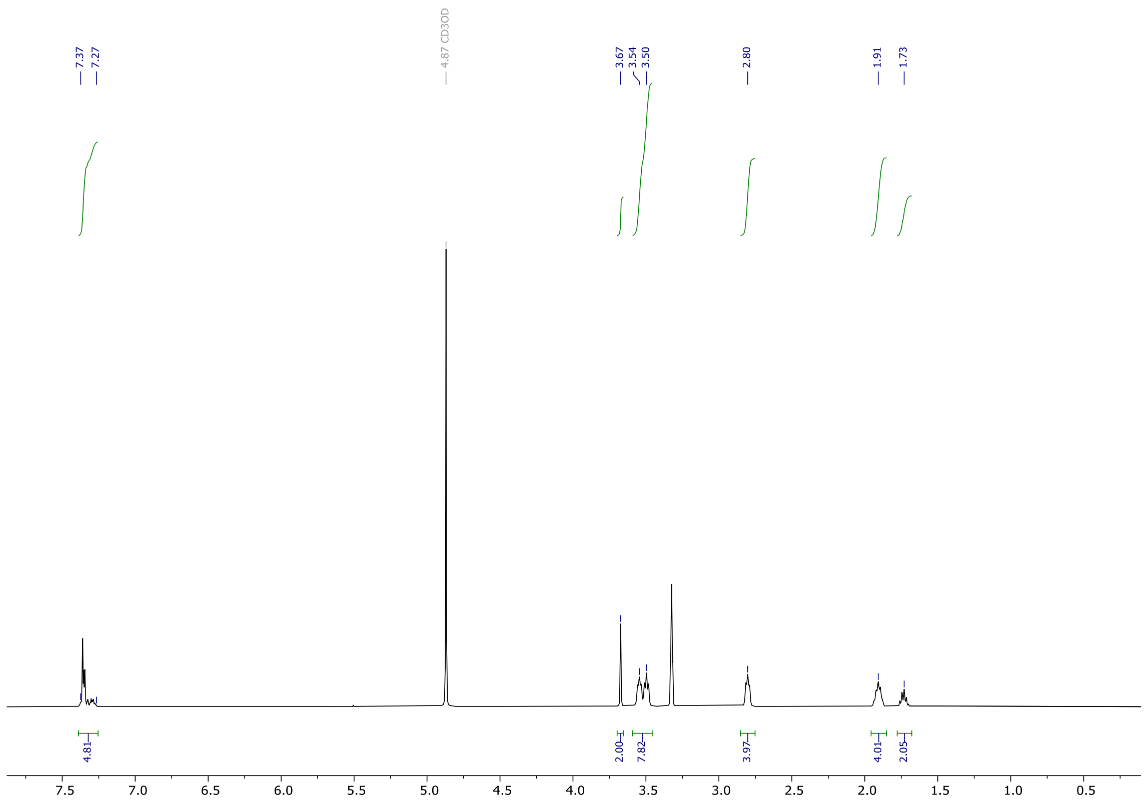

Figure S15: ^{1}H NMR spectrum (400 MHz) of **8** recorded in CD_3OD.

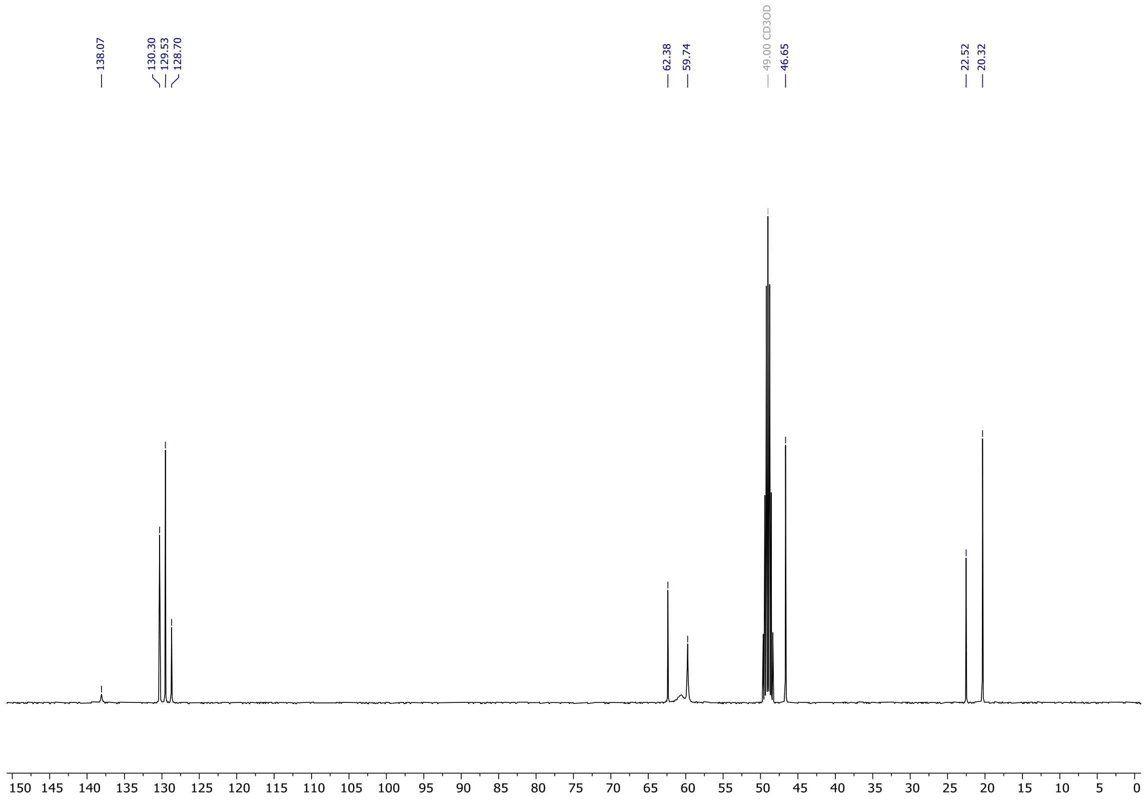

Figure S16: $^{13}C\{^1H\}$ NMR spectrum (101 MHz) of **8** recorded in CD_3OD.

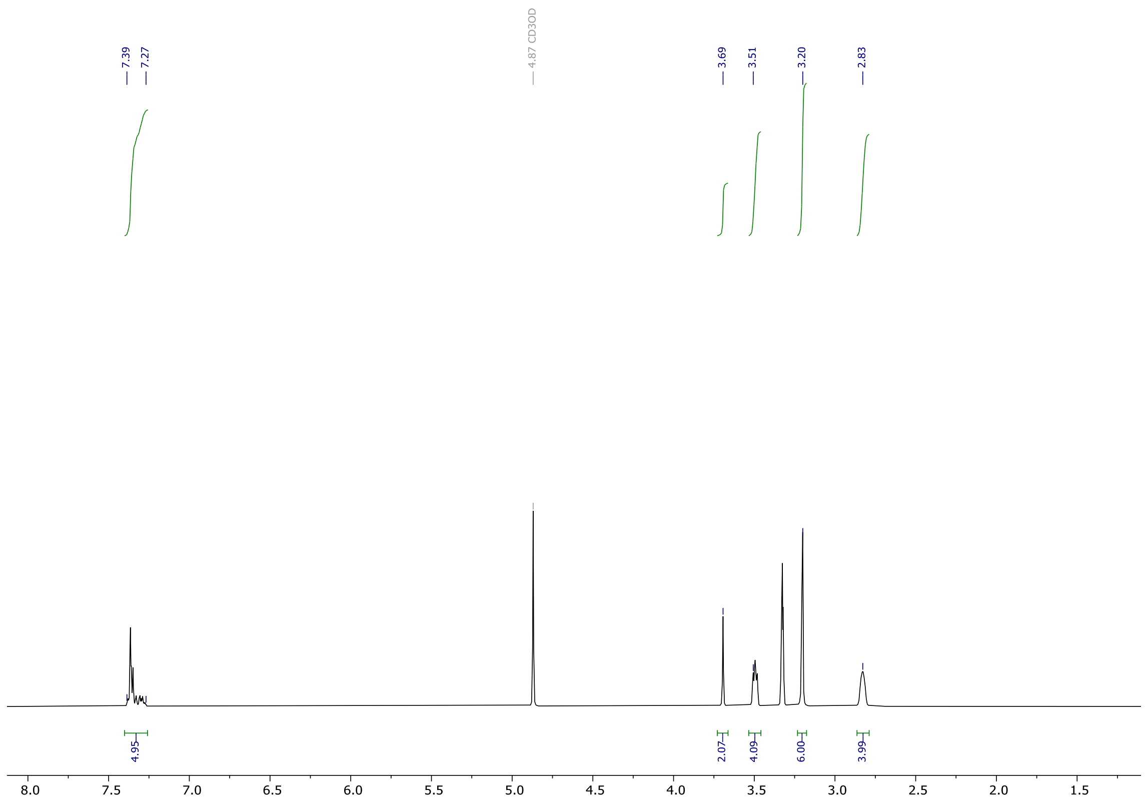

Figure S17: ^{1}H NMR spectrum (400 MHz) of **9** recorded in CD_3OD.

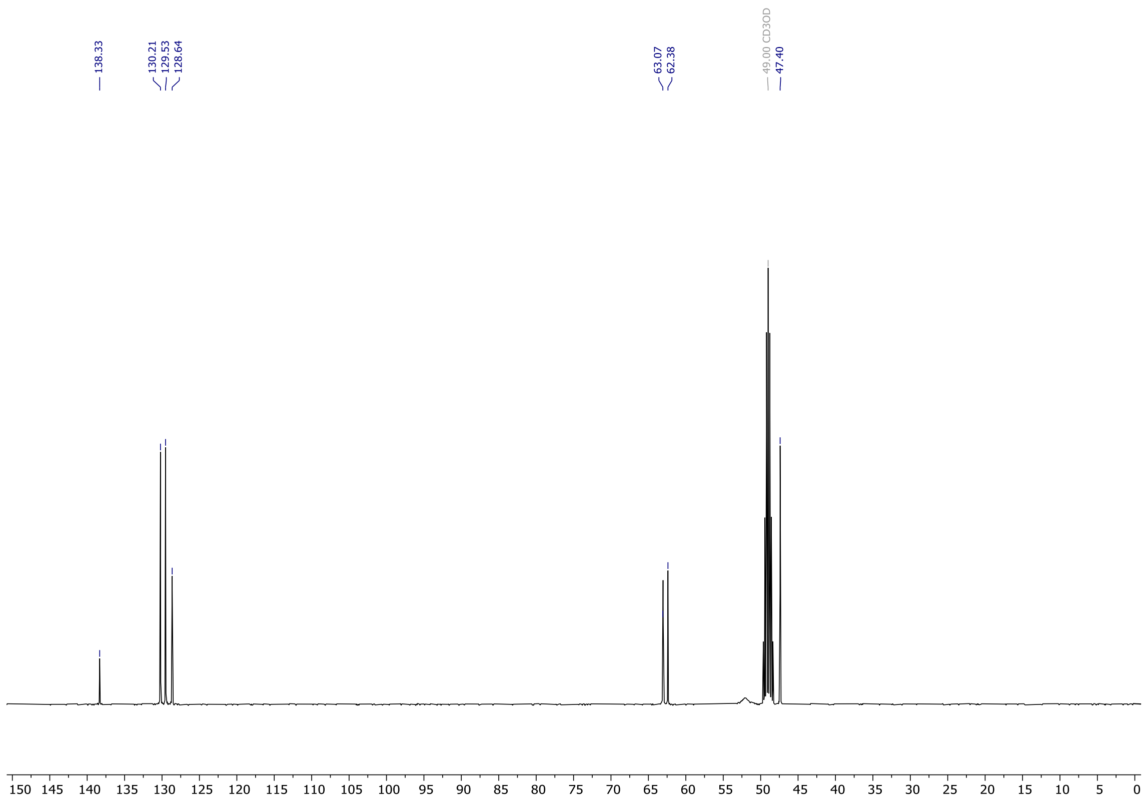

Figure S18: $^{13}C\{^{1}H\}$ NMR spectrum (101 MHz) of **9** recorded in CD_3OD.

X-ray Crystallography

Experimental

Single crystal diffraction data were collected on an XtaLAB Synergy HyPix-Arc 100 diffractometer using copper radiation ($\lambda_{CuK\alpha}$ = 1.54184 Å) at 150 K using an Oxford Cryosystems CryostreamPlus open-flow N_2 cooling device.

Intensities were corrected for absorption using a multifaceted crystal model created by indexing the faces of the crystal for which data were collected [1]. Cell refinement, data collection and data reduction were undertaken via the software CrysAlisPro [2].

All structures were solved using XT [3] and refined by XL [4] using the Olex2 interface [5]. All non-hydrogen atoms were refined anisotropically and hydrogen atoms were positioned with idealised geometry. The displacement parameters of the hydrogen atoms were constrained using a riding model with $U_{(H)}$ set to be an appropriate multiple of the U_{eq} value of the parent atom.

[1] R. C. Clark, J.S. Reid, *Acta Cryst.,* **1995**, A**51**, 887

[2] CrysAlisPro, Rigaku Oxford Diffraction, Tokyo, Japan.

[3] Sheldrick, G.M. *Acta Crystallogr., Sect. A: Found. Crystallogr.* **2015,** *71*, 3-8.

[4] Sheldrick, G.M. *Acta Crystallogr., Sect. A: Found. Crystallogr.* **2008,** *64*, 112-122.

[5] Dolomanov, O.V.; Bourhis, L.J.; Gildea, R.J.; Howard, J.A.K.; Puschmann, H. *J. Appl. Cryst.* **2009,** *42*, 339-341.

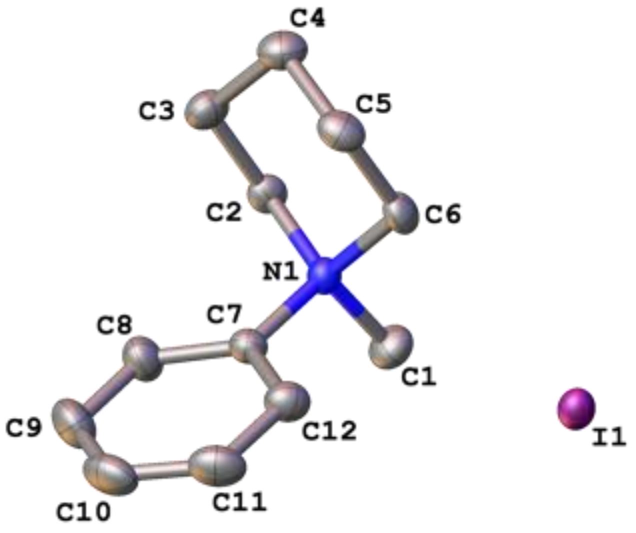

Figure S19: Molecular structure of **1** as determined by single-crystal X-ray diffraction; thermal ellipsoids at 50 % probability, hydrogen atoms oitted for clarity.

Table S1: Crystal data and structure refinement for 1

Identification code	**1**
Empirical formula	$C_{12}H_{18}IN$
Formula weight	303.17
Temperature/K	150.0(2)
Crystal system	monoclinic
Space group	$P2_1/c$
a/Å	10.33590(10)
b/Å	18.62180(10)
c/Å	13.07300(10)
α/°	90
β/°	91.7790(10)
γ/°	90
Volume/Å^3	2514.99(3)
Z	8
ρ_{calc}g/cm^3	1.601
μ/mm^{-1}	19.710
F(000)	1200.0
Crystal size/mm^3	0.18 × 0.09 × 0.02
Radiation	CuKα (λ = 1.54184)
2Θ range for data collection/°	8.266 to 153.672
Index ranges	-12 ≤ h ≤ 12, -22 ≤ k ≤ 23, -16 ≤ l ≤ 14
Reflections collected	29835
Independent reflections	5109 [R_{int} = 0.0383, R_{sigma} = 0.0246]
Data/restraints/parameters	5109/0/255
Goodness-of-fit on F^2	1.059
Final R indexes [I>=2σ (I)]	R_1 = 0.0197, wR_2 = 0.0462
Final R indexes [all data]	R_1 = 0.0224, wR_2 = 0.0472
Largest diff. peak/hole / e Å^{-3}	0.34/-0.45

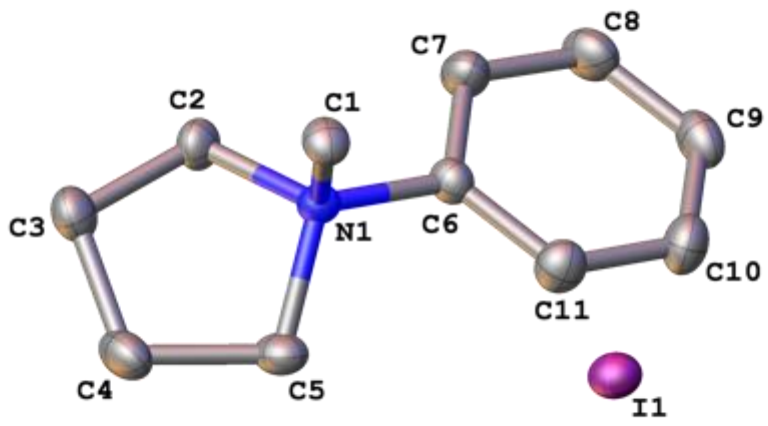

Figure S20: Molecular structure of **2** as determined by single crystal X-ray diffraction; thermal ellipsoids at 50 % probability, hydrogen atoms omitted for clarity.

Table S2: Crystal data and structure refinement for 2

Identification code	**2**
Empirical formula	$C_{11}H_{16}IN$
Formula weight	289.15
Temperature/K	150.0(2)
Crystal system	monoclinic
Space group	$P2_1/c$
a/Å	12.3872(2)
b/Å	9.91560(10)
c/Å	10.1793(2)
α/°	90
β/°	109.906(2)
γ/°	90
Volume/Å^3	1175.59(4)
Z	4
ρ_{calc}g/cm^3	1.634
μ/mm^{-1}	21.052
F(000)	568.0
Crystal size/mm^3	0.35 × 0.08 × 0.04
Radiation	CuKα (λ = 1.54184)
2Θ range for data collection/°	7.59 to 152.33
Index ranges	-14 ≤ h ≤ 15, -4 ≤ k ≤ 12, -12 ≤ l ≤ 12
Reflections collected	7626
Independent reflections	2271 [R_{int} = 0.0330, R_{sigma} = 0.0315]
Data/restraints/parameters	2271/0/120
Goodness-of-fit on F^2	1.058
Final R indexes [I>=2σ (I)]	R_1 = 0.0237, wR_2 = 0.0598
Final R indexes [all data]	R_1 = 0.0255, wR_2 = 0.0610
Largest diff. peak/hole / e Å^{-3}	0.90/-0.58

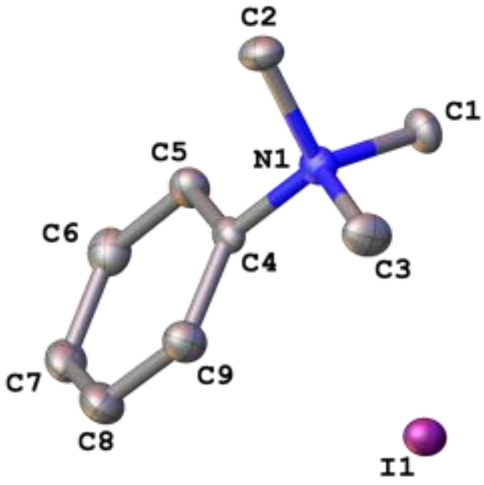

Figure S21: Molecular structure of **4** as determined by single crystal X-ray diffraction; thermal ellipsoids at 50 % probability, hydrogen atoms omitted for clarity.

Table S3: Crystal data and structure refinement for 4

Identification code	**4**
Empirical formula	$C_9H_{14}IN$
Formula weight	263.11
Temperature/K	150.0(2)
Crystal system	orthorhombic
Space group	$P2_12_12_1$
a/Å	5.7655(2)
b/Å	7.9152(2)
c/Å	22.1901(6)
α/°	90
β/°	90
γ/°	90
Volume/Å^3	1012.65(5)
Z	4
ρ_{calc}g/cm^3	1.726
μ/mm^{-1}	24.368
F(000)	512.0
Crystal size/mm^3	0.22 × 0.09 × 0.03
Radiation	CuKα (λ = 1.54184)
2Θ range for data collection/°	7.968 to 152.088
Index ranges	-6 ≤ h ≤ 6, -8 ≤ k ≤ 9, -26 ≤ l ≤ 25
Reflections collected	4721
Independent reflections	1939 [R_{int} = 0.0372, R_{sigma} = 0.0438]
Data/restraints/parameters	1939/0/104
Goodness-of-fit on F^2	1.026
Final R indexes [I>=2σ (I)]	R_1 = 0.0259, wR_2 = 0.0605
Final R indexes [all data]	R_1 = 0.0280, wR_2 = 0.0622
Largest diff. peak/hole / e Å^{-3}	0.78/-0.75
Flack parameter	-0.015(13)

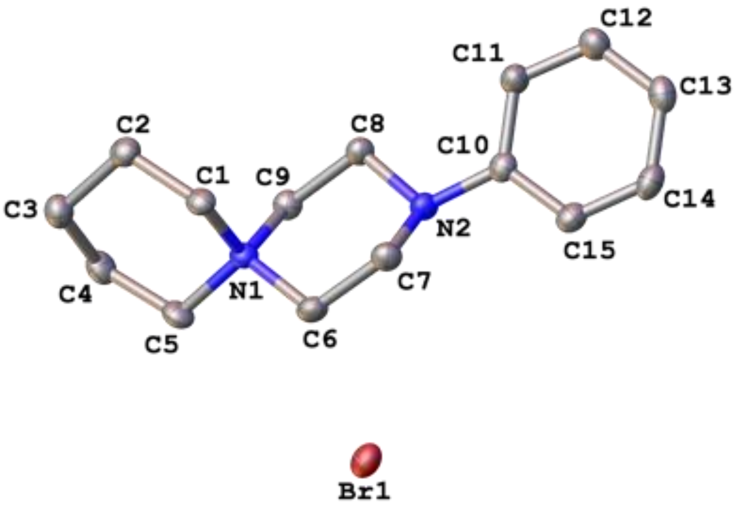

Figure S22: Molecular structure of **5** as determined by single crystal X-ray diffraction; thermal ellipsoids at 50 % probability, hydrogen atoms omitted for clarity.

Table S4: Crystal data and structure refinement for 5

Identification code	**5**
Empirical formula	$C_{15}H_{23}BrN_2$
Formula weight	311.26
Temperature/K	150.0(2)
Crystal system	monoclinic
Space group	$P2_1/c$
a/Å	9.24030(10)
b/Å	15.5328(2)
c/Å	9.91870(10)
α/°	90
β/°	94.9070(10)
γ/°	90
Volume/Å^3	1418.39(3)
Z	4
ρ_{calc}g/cm^3	1.458
μ/mm^{-1}	3.810
F(000)	648.0
Crystal size/mm^3	0.13 × 0.12 × 0.05
Radiation	CuKα (λ = 1.54184)
2Θ range for data collection/°	9.606 to 154.212
Index ranges	-10 ≤ h ≤ 11, -18 ≤ k ≤ 18, -12 ≤ l ≤ 12
Reflections collected	16681
Independent reflections	2839 [R_{int} = 0.0392, R_{sigma} = 0.0248]
Data/restraints/parameters	2839/0/163
Goodness-of-fit on F^2	1.070
Final R indexes [I>=2σ (I)]	R_1 = 0.0235, wR_2 = 0.0601
Final R indexes [all data]	R_1 = 0.0255, wR_2 = 0.0614
Largest diff. peak/hole / e Å^{-3}	0.31/-0.37

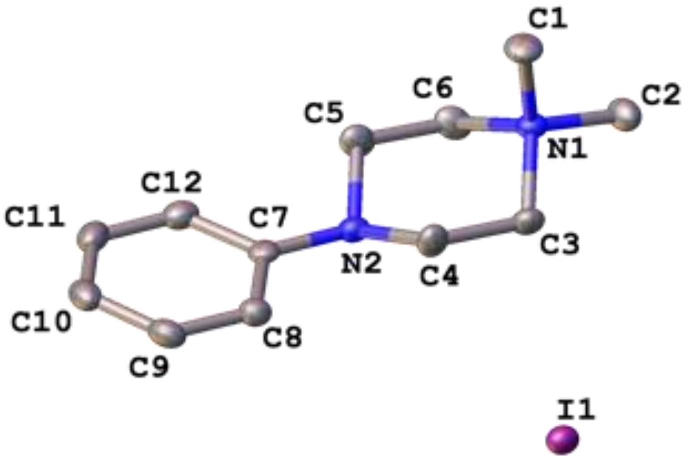

Figure S23: Molecular structure of **6** as determined by single crystal X-ray diffraction; thermal ellipsoids at 50 % probability, hydrogen atoms omitted for clarity.

Table S5: Crystal data and structure refinement for 6

Identification code	**6**
Empirical formula	$C_{12}H_{19}IN_2$
Formula weight	318.19
Temperature/K	150.0(2)
Crystal system	monoclinic
Space group	$P2_1/c$
a/Å	6.22030(10)
b/Å	7.68290(10)
c/Å	27.5077(3)
α/°	90
β/°	96.4140(10)
γ/°	90
Volume/Å^3	1306.36(3)
Z	4
ρ_{calc}g/cm^3	1.618
μ/mm^{-1}	19.026
F(000)	632.0
Crystal size/mm^3	0.21 × 0.11 × 0.06
Radiation	CuKα (λ = 1.54184)
2Θ range for data collection/°	6.466 to 153.816
Index ranges	-6 ≤ h ≤ 7, -9 ≤ k ≤ 9, -33 ≤ l ≤ 34
Reflections collected	15139
Independent reflections	2614 [R_{int} = 0.0461, R_{sigma} = 0.0288]
Data/restraints/parameters	2614/0/139
Goodness-of-fit on F^2	1.066
Final R indexes [I>=2σ (I)]	R_1 = 0.0260, wR_2 = 0.0730
Final R indexes [all data]	R_1 = 0.0287, wR_2 = 0.0756
Largest diff. peak/hole / e Å^{-3}	0.93/-0.73

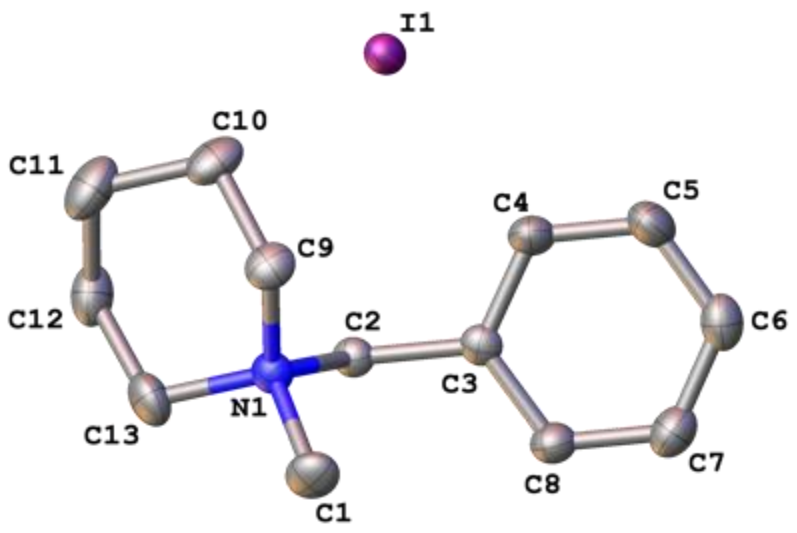

Figure S24: Molecular structure of **7** as determined by single crystal X-ray diffraction; thermal ellipsoids at 50 % probability, hydrogen atoms omitted for clarity.

Table S6: Crystal data and structure refinement for 7

Identification code	**7**
Empirical formula	$C_{13}H_{20}IN$
Formula weight	317.20
Temperature/K	150.0(2)
Crystal system	orthorhombic
Space group	Pbca
a/Å	9.64100(10)
b/Å	16.3899(2)
c/Å	17.1888(2)
α/°	90
β/°	90
γ/°	90
Volume/Å^3	2716.09(5)
Z	8
ρ_{calc}g/cm^3	1.551
μ/mm^{-1}	18.278
F(000)	1264.0
Crystal size/mm^3	0.16 × 0.12 × 0.03
Radiation	CuKα (λ = 1.54184)
2Θ range for data collection/°	10.292 to 153.842
Index ranges	-12 ≤ h ≤ 10, -19 ≤ k ≤ 19, -21 ≤ l ≤ 17
Reflections collected	13994
Independent reflections	2704 [R_{int} = 0.0352, R_{sigma} = 0.0241]
Data/restraints/parameters	2704/0/137
Goodness-of-fit on F^2	1.053
Final R indexes [I>=2σ (I)]	R_1 = 0.0241, wR_2 = 0.0699
Final R indexes [all data]	R_1 = 0.0275, wR_2 = 0.0726
Largest diff. peak/hole / e Å^{-3}	0.96/-0.75

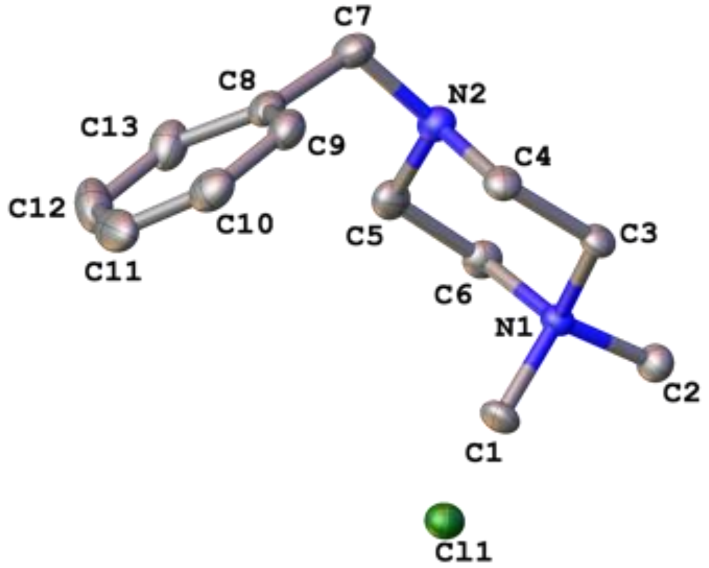

Figure S25: Molecular structure of **9** as determined by single crystal X-ray diffraction; thermal ellipsoids at 50 % probability, hydrogen atoms omitted for clarity.

Table S7: Crystal data and structure refinement for 9

Identification code	**9**
Empirical formula	$C_{13}H_{21}ClN_2$
Formula weight	240.77
Temperature/K	150.0(2)
Crystal system	monoclinic
Space group	$P2_1$
a/Å	6.36860(10)
b/Å	9.7589(3)
c/Å	10.6030(2)
α/°	90
β/°	96.219(2)
γ/°	90
Volume/Å^3	655.10(3)
Z	2
ρ_{calc}g/cm^3	1.221
μ/mm^{-1}	2.372
F(000)	260.0
Crystal size/mm^3	0.15 × 0.12 × 0.11
Radiation	CuKα (λ = 1.54184)
2Θ range for data collection/°	8.388 to 153.88
Index ranges	-8 ≤ h ≤ 7, -11 ≤ k ≤ 11, -12 ≤ l ≤ 13
Reflections collected	6621
Independent reflections	2240 [R_{int} = 0.0297, R_{sigma} = 0.0305]
Data/restraints/parameters	2240/1/148
Goodness-of-fit on F^2	1.053
Final R indexes [I>=2σ (I)]	R_1 = 0.0266, wR_2 = 0.0677
Final R indexes [all data]	R_1 = 0.0272, wR_2 = 0.0685
Largest diff. peak/hole / e Å^{-3}	0.17/-0.14
Flack parameter	-0.019(9)

Headgroup Alkaline Stability Studies

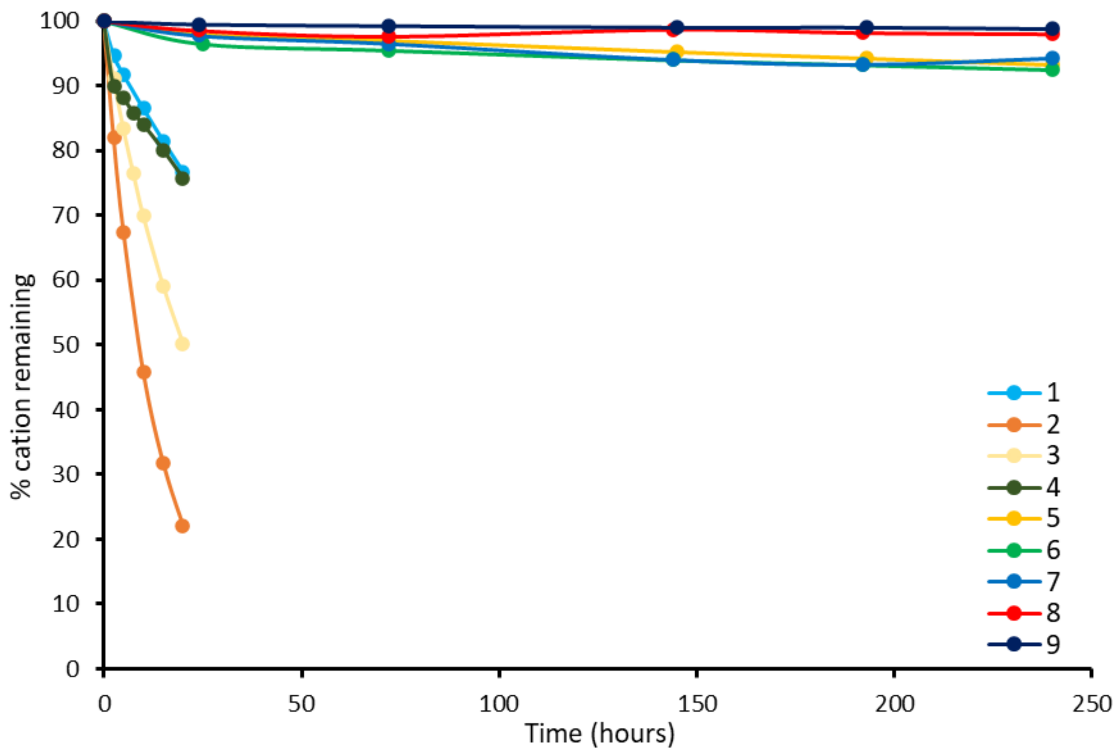

Figure S26: Percentage of headgroups **1 – 9** remaining in 3M NaOD/D_2O/CD_3OD solution at 60 °C as a function of time.

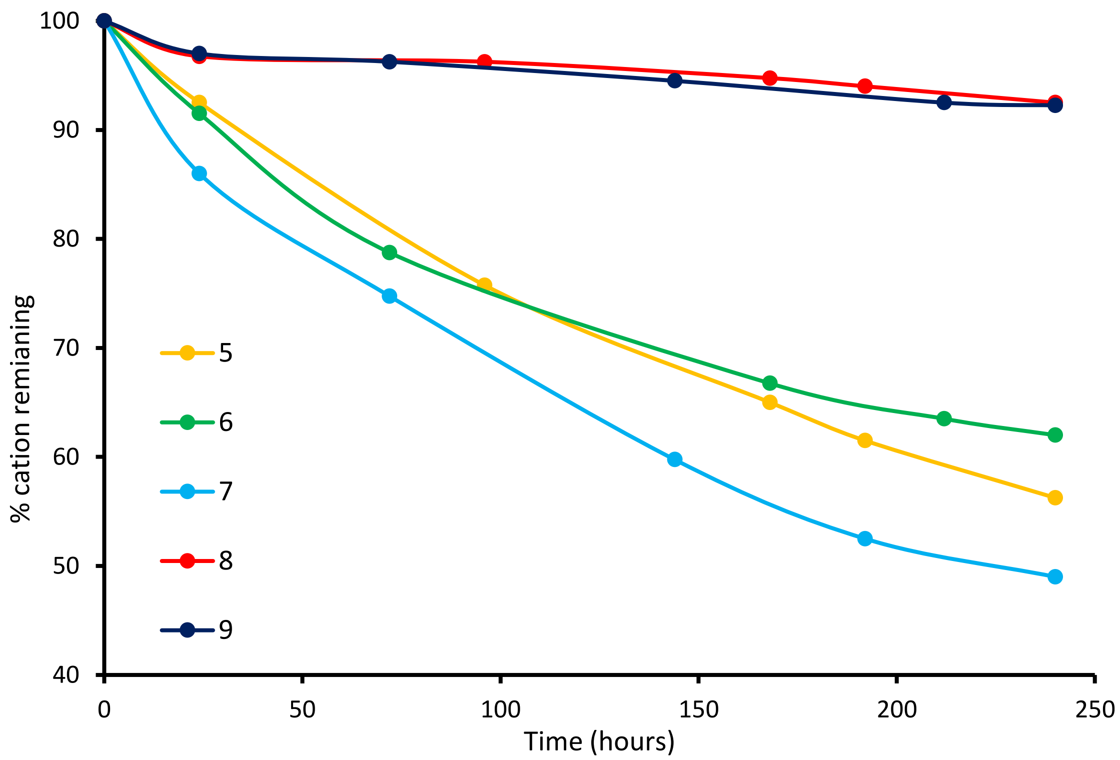

Figure S27: Percentage of headgroups **5 – 9** remaining in 3M NaOD/D_2O/CD_3OD solution at 80 °C as a function of time.

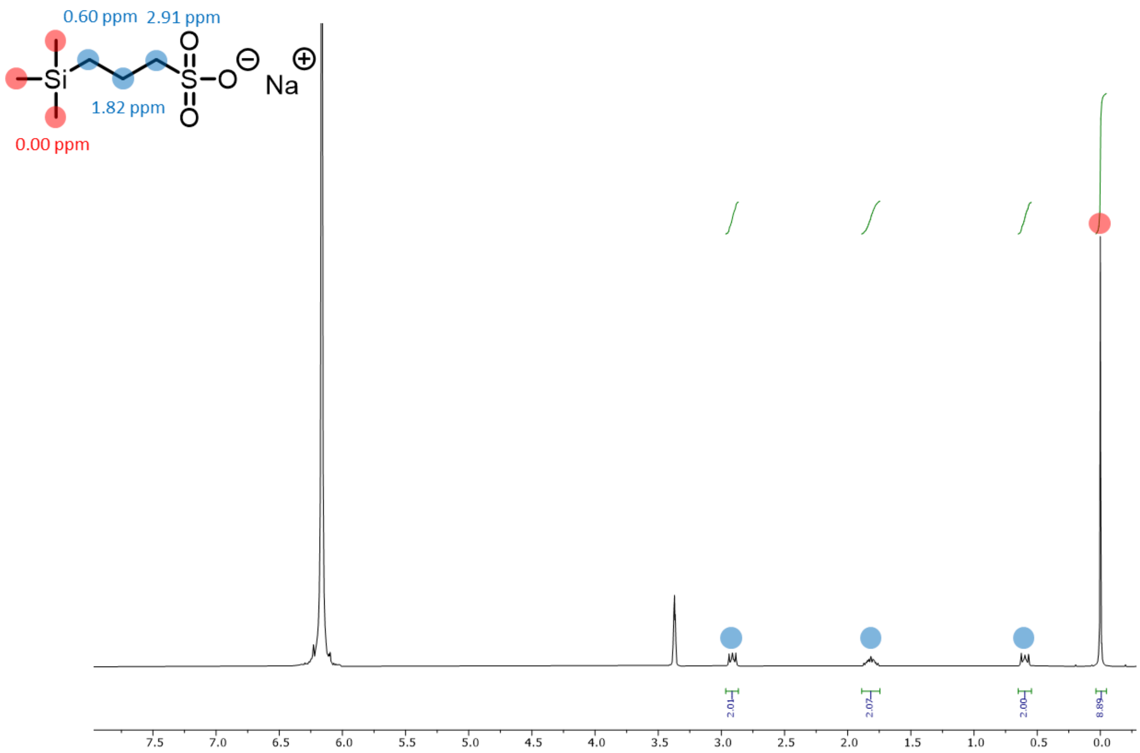

Figure S28: ^{1}H NMR spectra (300 MHz) of internal standard (NaDSS) dissolved in a 3M NaOD/D_2O/CD_3OD solution.

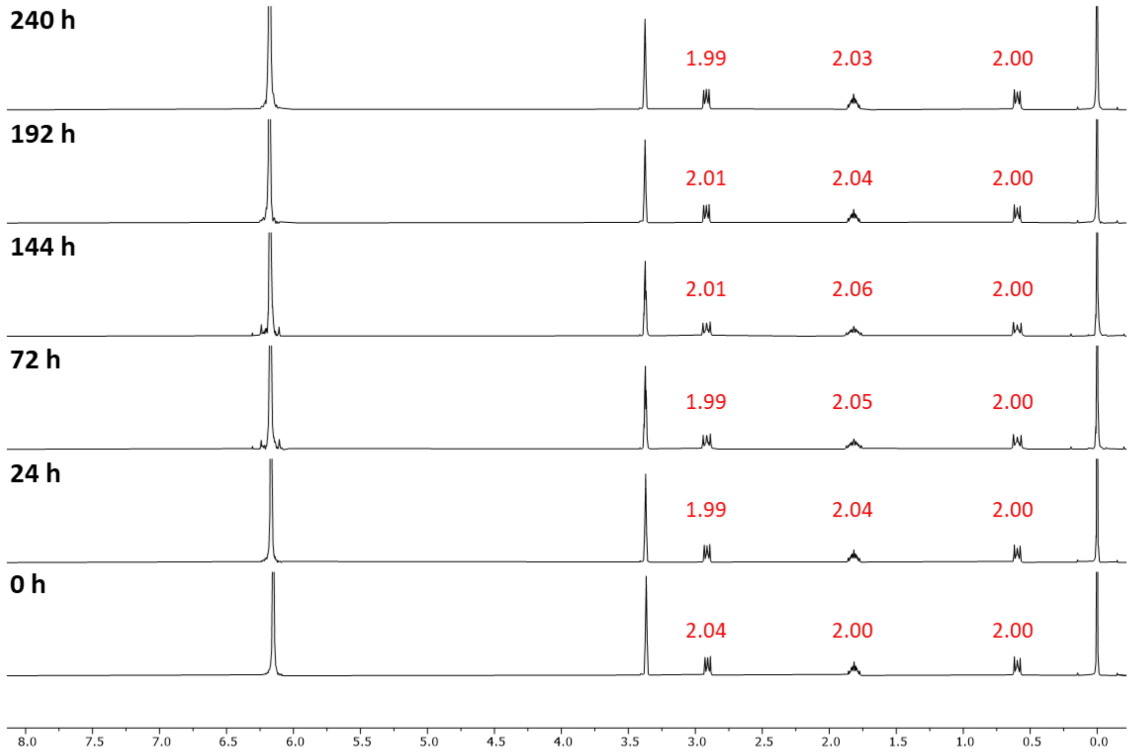

Figure S29: ^{1}H NMR spectra (400 MHz) of internal standard (NaDSS) over 240 hours dissolved in a 3M NaOD/D_2O/CD_3OD solution at 60 °C. Calculated integrals are included to show no degradation or H/D exchange occurs.

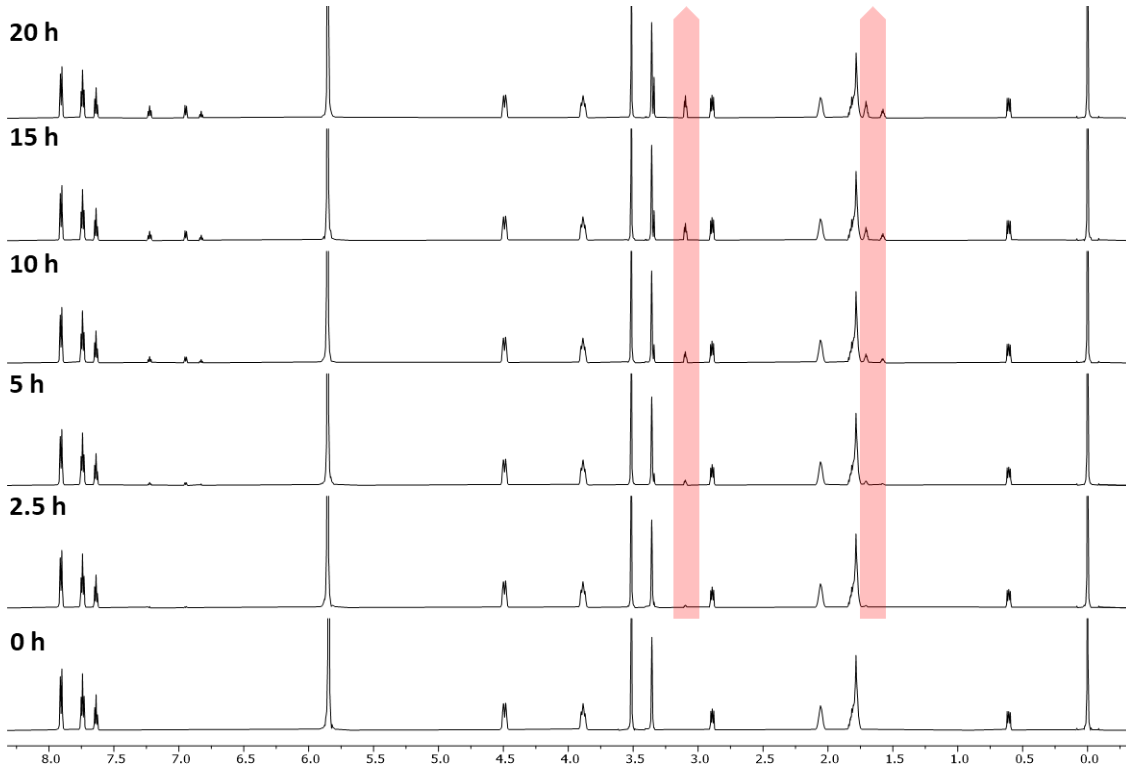

Figure S30: ^{1}H NMR spectra (700 MHz) of **1** over 20 hours dissolved in a 3M NaOD/D_2O/CD_3OD solution with internal standard (NaDSS) at 60 °C.

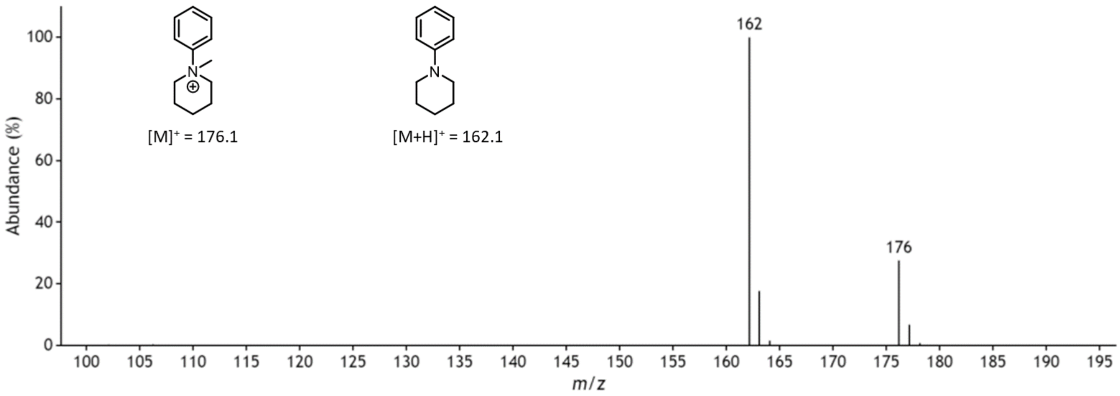

Figure S31: MS of **1** after 20 hours dissolved in a 3M NaOD/D_2O/CD_3OD solution.

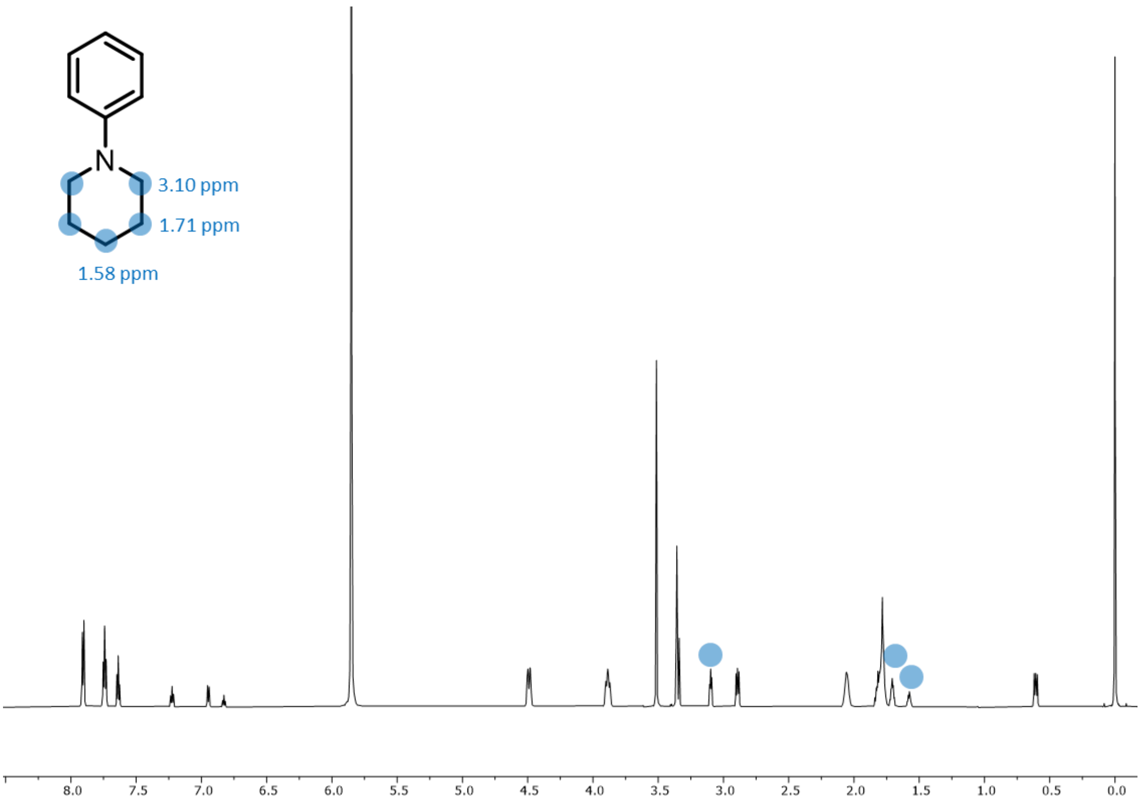

Figure S32: ^{1}H NMR spectra (700 MHz) of **1** after 20 hours dissolved in a 3M NaOD/D_2O/CD_3OD solution with an internal standard (NaDSS) at 60 °C, degradation product assigned.

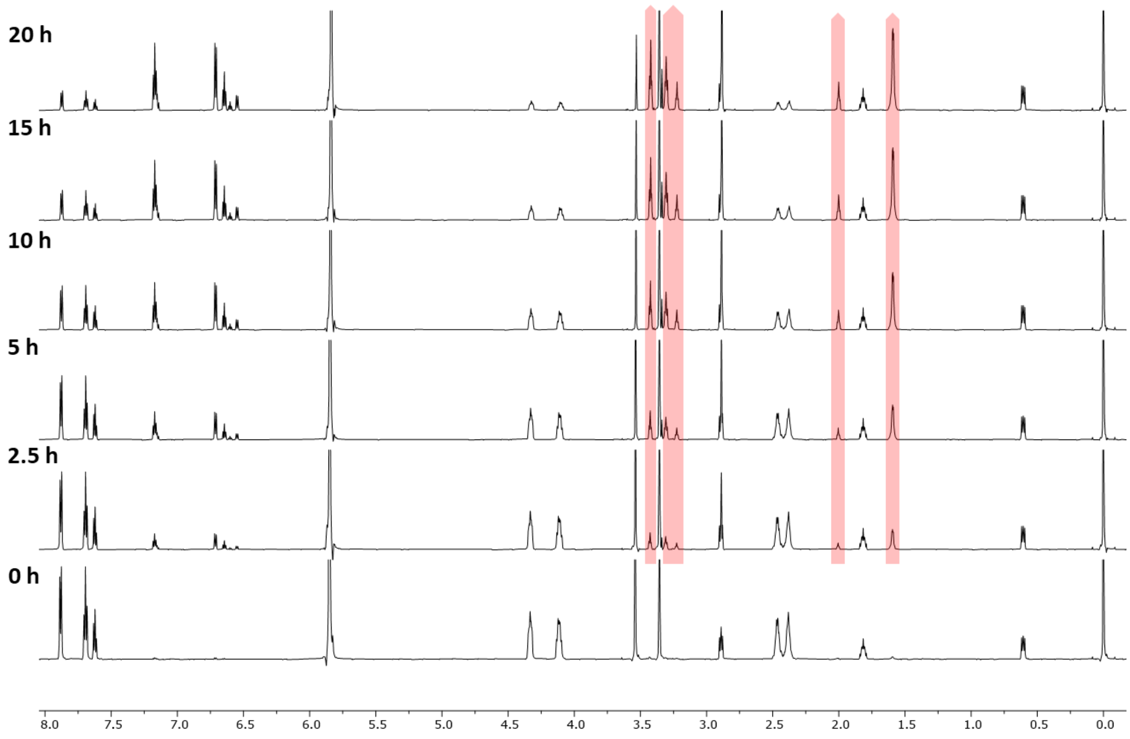

Figure S33: ^{1}H NMR spectra (700 MHz) of **2** over 20 hours dissolved in a 3M NaOD/D_2O/CD_3OD solution with internal standard (NaDSS) at 60 °C.

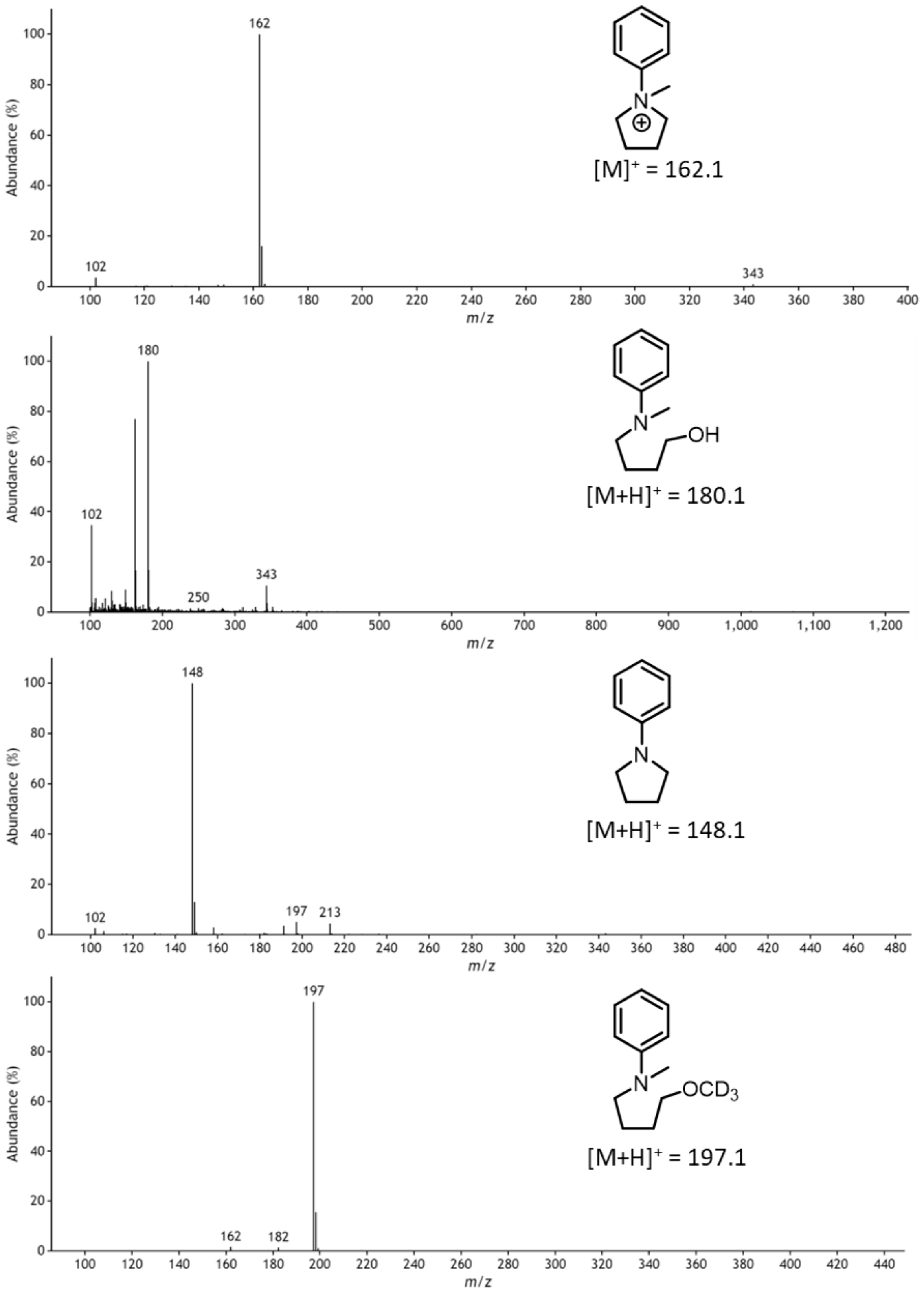

Figure S34: MS of **2** after 20 hours dissolved in a 3M NaOD/D_2O/CD_3OD solution.

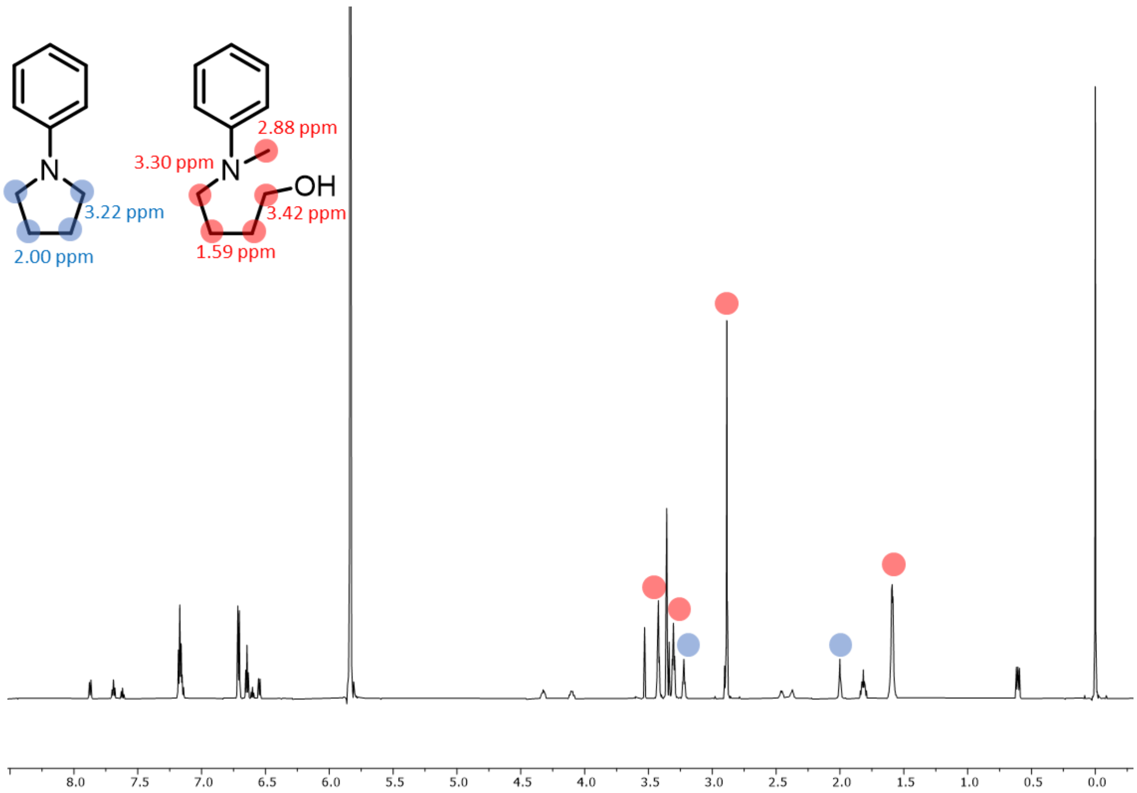

Figure S35: ^{1}H NMR spectra (700 MHz) of **2** after 20 hours dissolved in a 3M NaOD/D_2O/CD_3OD solution with an internal standard (NaDSS) at 60 °C, degradation products assigned.

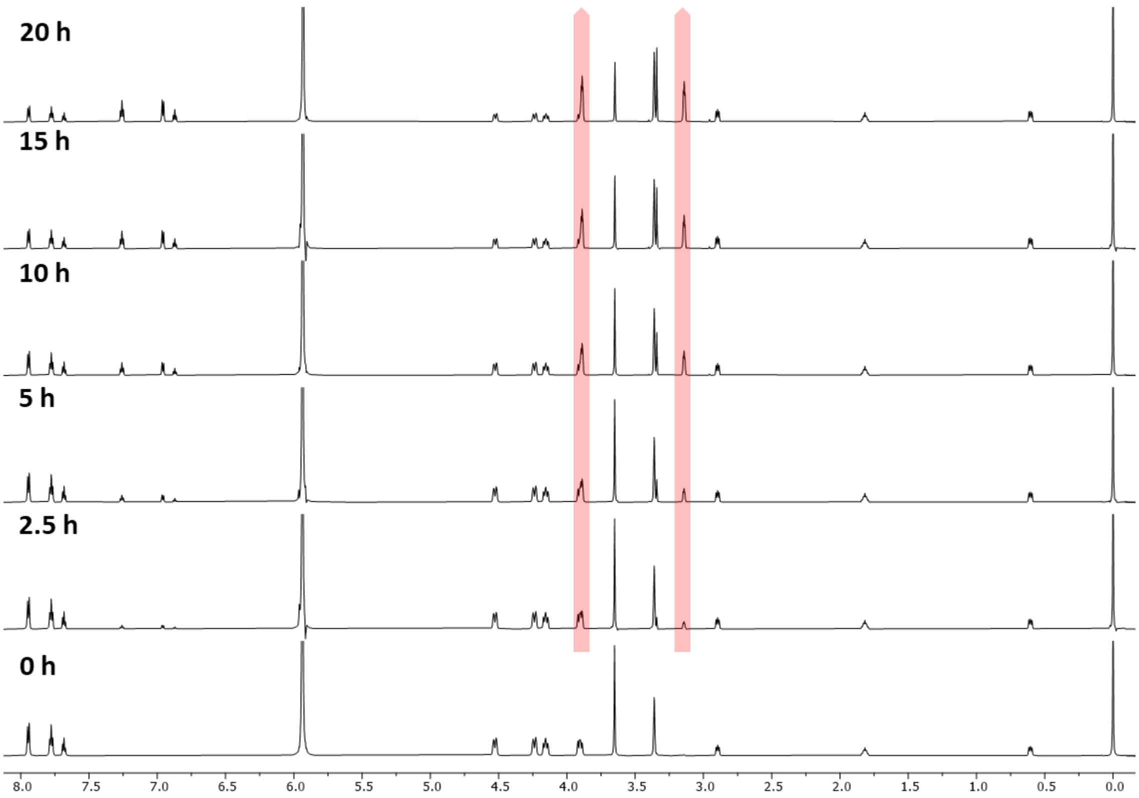

Figure S36: ^{1}H NMR spectra (700 MHz) of **3** over 20 hours dissolved in a 3M NaOD/D_2O/CD_3OD solution with internal standard (NaDSS) at 60 °C.

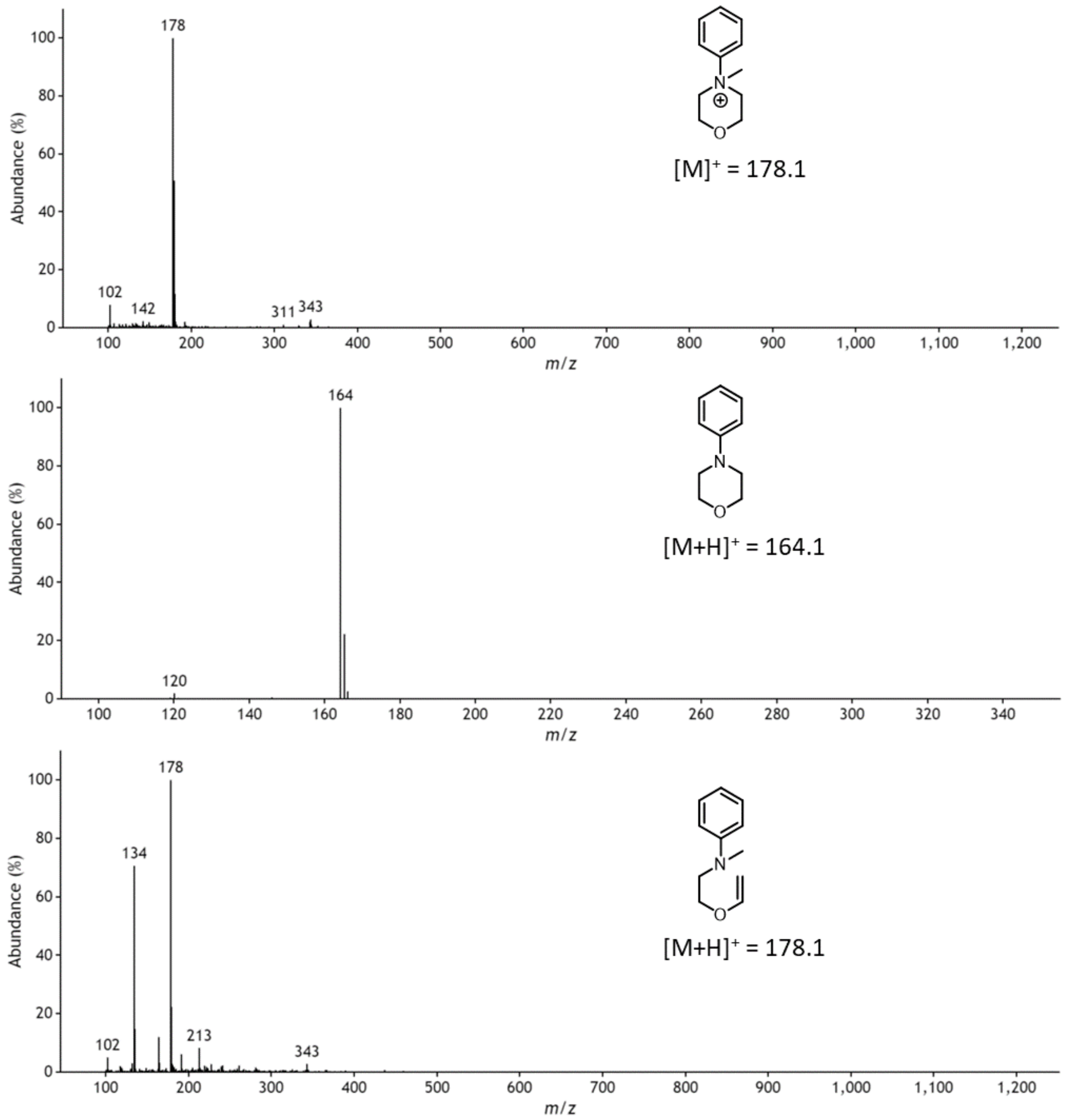

Figure S37: MS of **3** after 20 hours dissolved in a 3M NaOD/D_2O/CD_3OD solution.

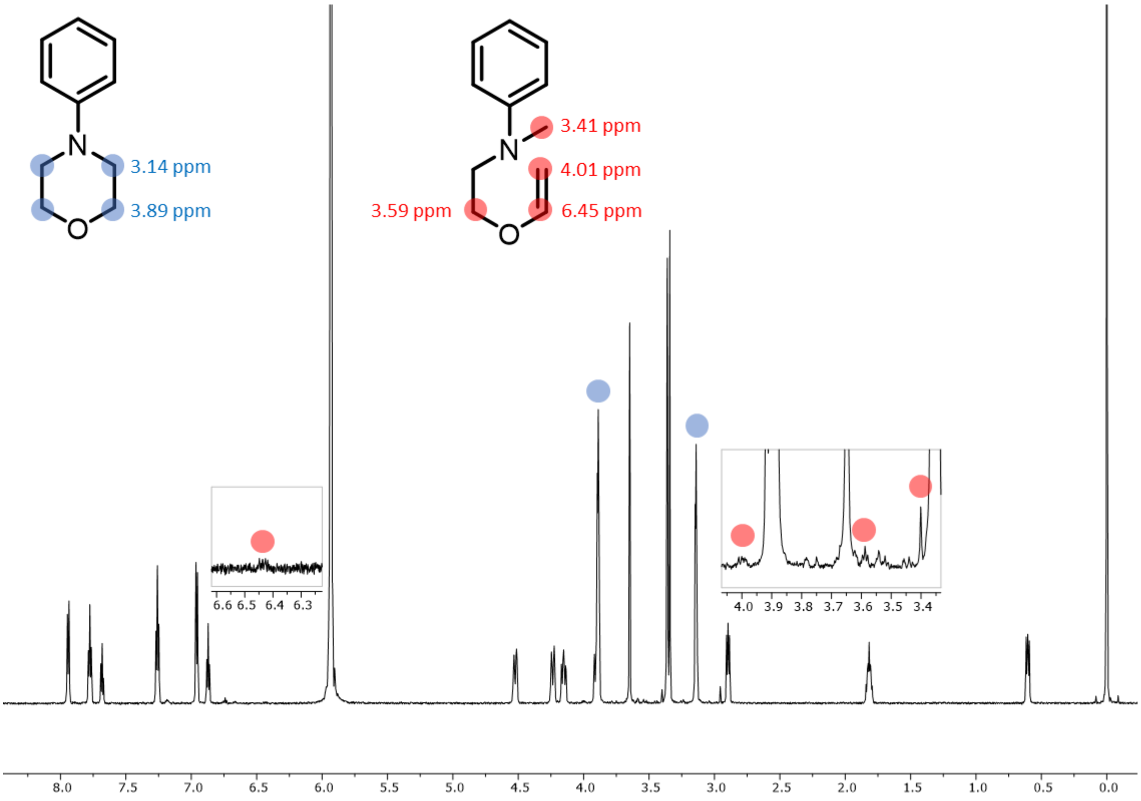

Figure S38: ^{1}H NMR spectra (700 MHz) of **3** after 20 hours dissolved in a 3M NaOD/D_2O/CD_3OD solution with an internal standard (NaDSS) at 60 °C, degradation products assigned.

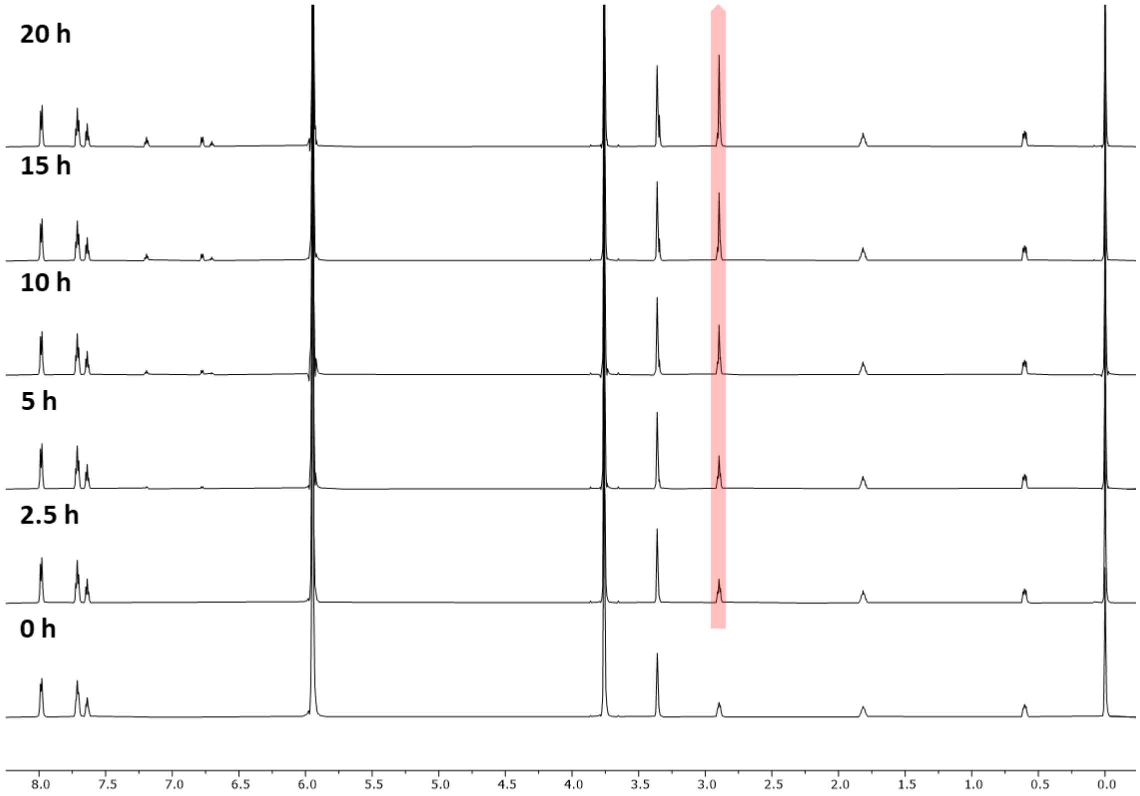

Figure S39: ^{1}H NMR spectra (700 MHz) of **4** over 20 hours dissolved in a 3M NaOD/D_2O/CD_3OD solution with internal standard (NaDSS) at 60 °C.

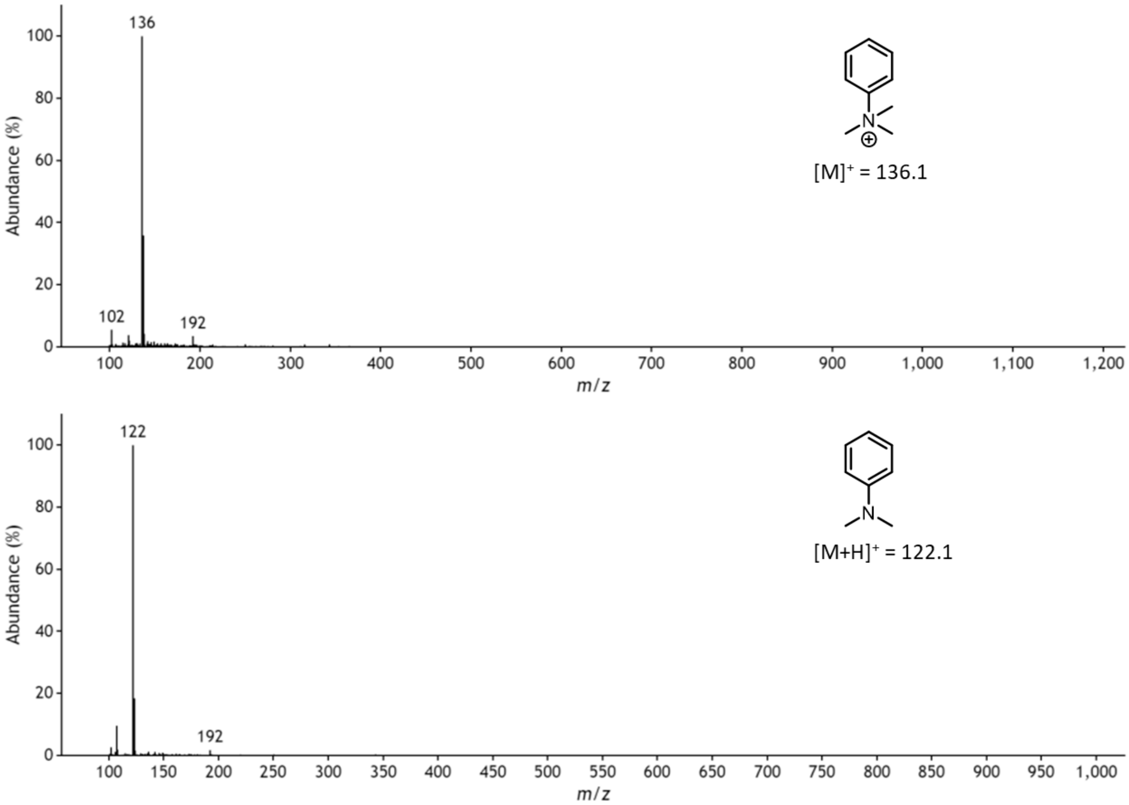

Figure S40: MS of **4** after 20 hours dissolved in a 3M NaOD/D_2O/CD_3OD solution.

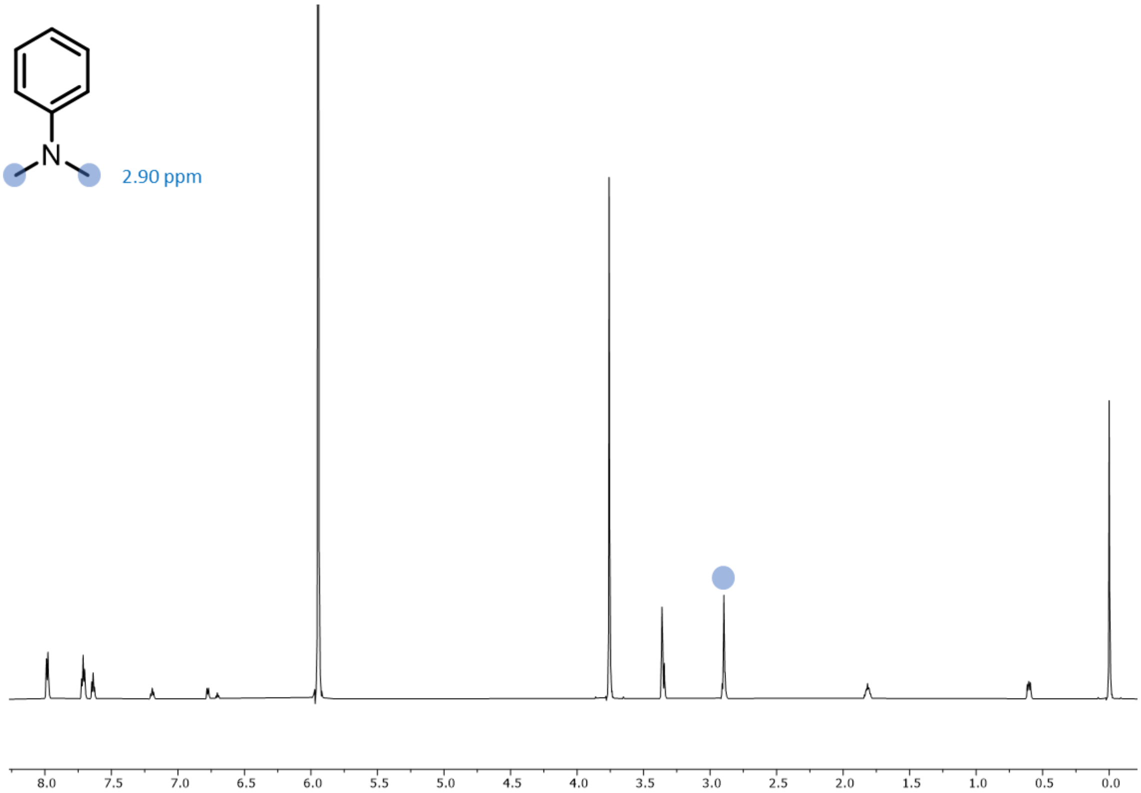

Figure S41: ^{1}H NMR spectra (700 MHz) of **4** after 20 hours dissolved in a 3M NaOD/D_2O/CD_3OD solution with internal standard (NaDSS) at 60 °C, degradation product assigned.

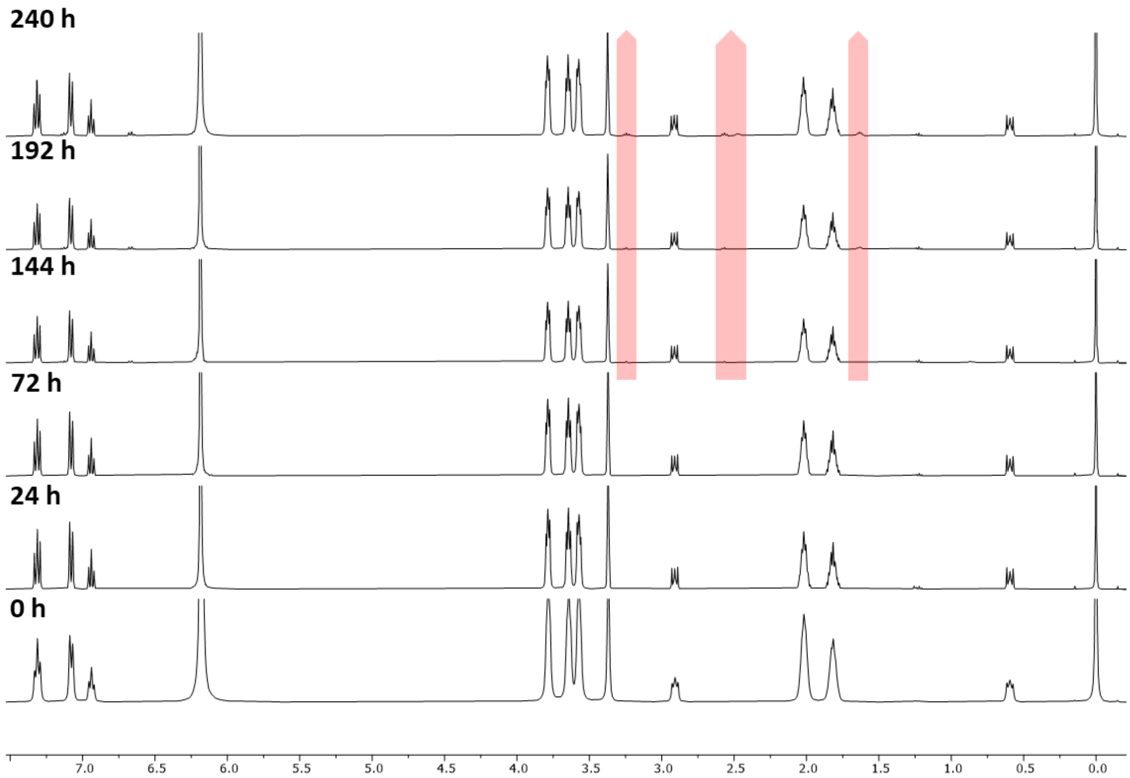

Figure S42: ^{1}H NMR spectra (400 MHz) of **5** over 240 hours dissolved in a 3M NaOD/D_2O/CD_3OD solution with internal standard (NaDSS) at 60 °C.

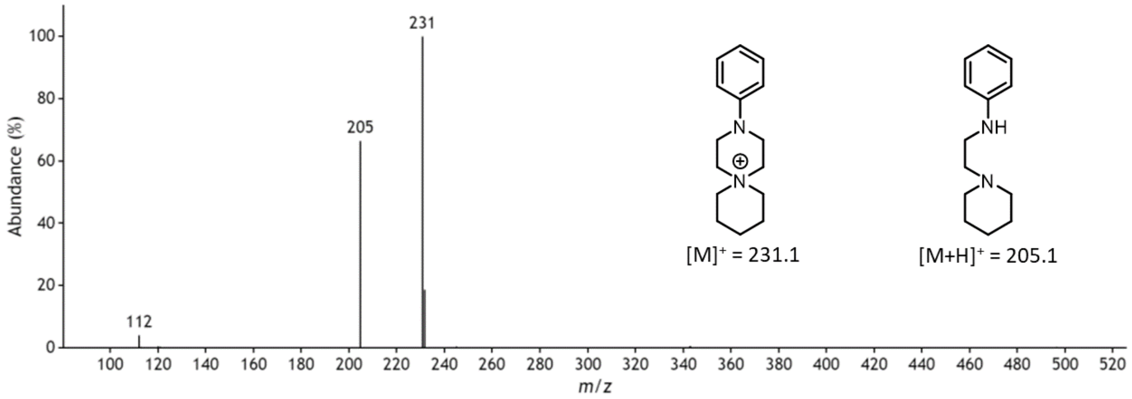

Figure S43: MS of **5** after 240 hours dissolved in a 3M NaOD/D_2O/CD_3OD solution.

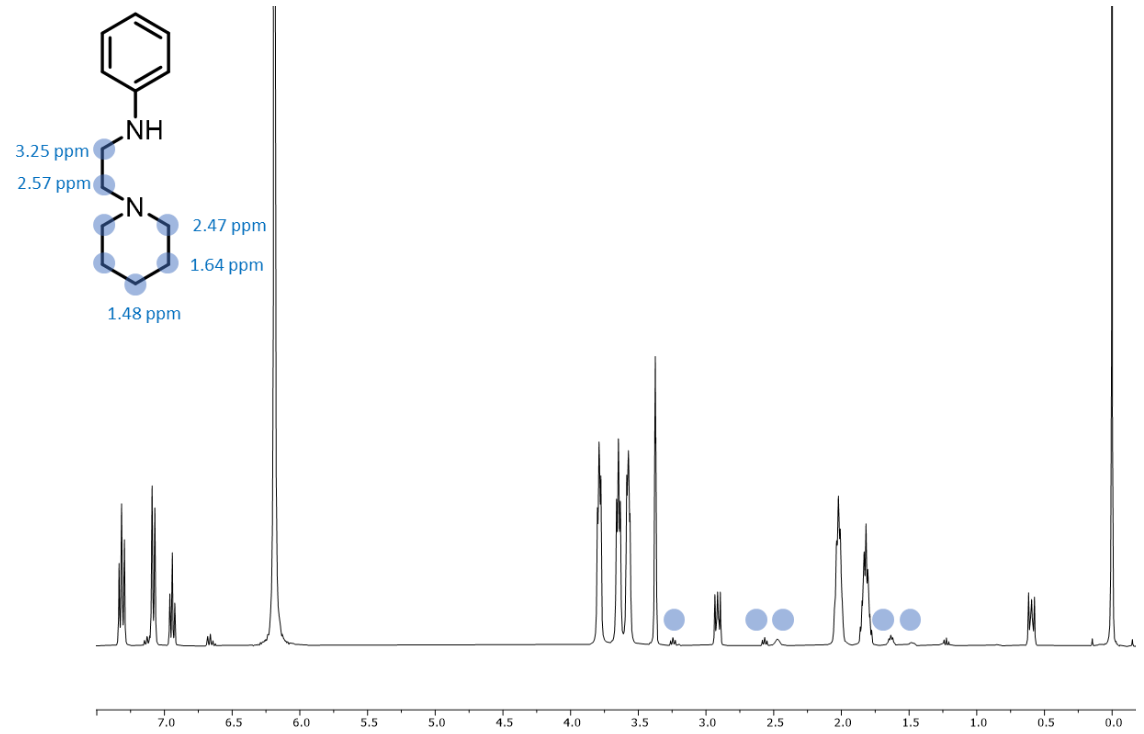

Figure S44: ^{1}H NMR spectra (400 MHz) of **5** after 240 hours dissolved in a 3M $NaOD/D_2O/CD_3OD$ solution with internal standard (NaDSS) at 60 °C, degradation product assigned.

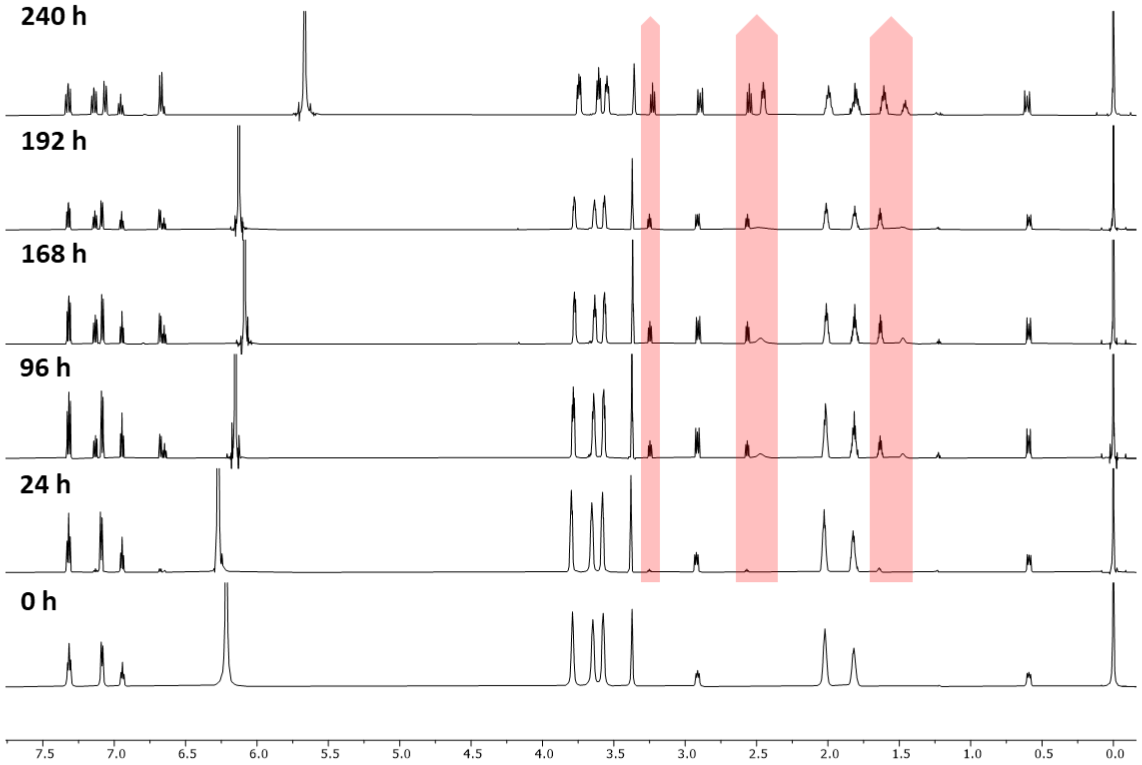

Figure S45: ^{1}H NMR spectra (700 MHz) of **5** over 240 hours dissolved in a 3M $NaOD/D_2O/CD_3OD$ solution with internal standard (NaDSS) at 80 °C.

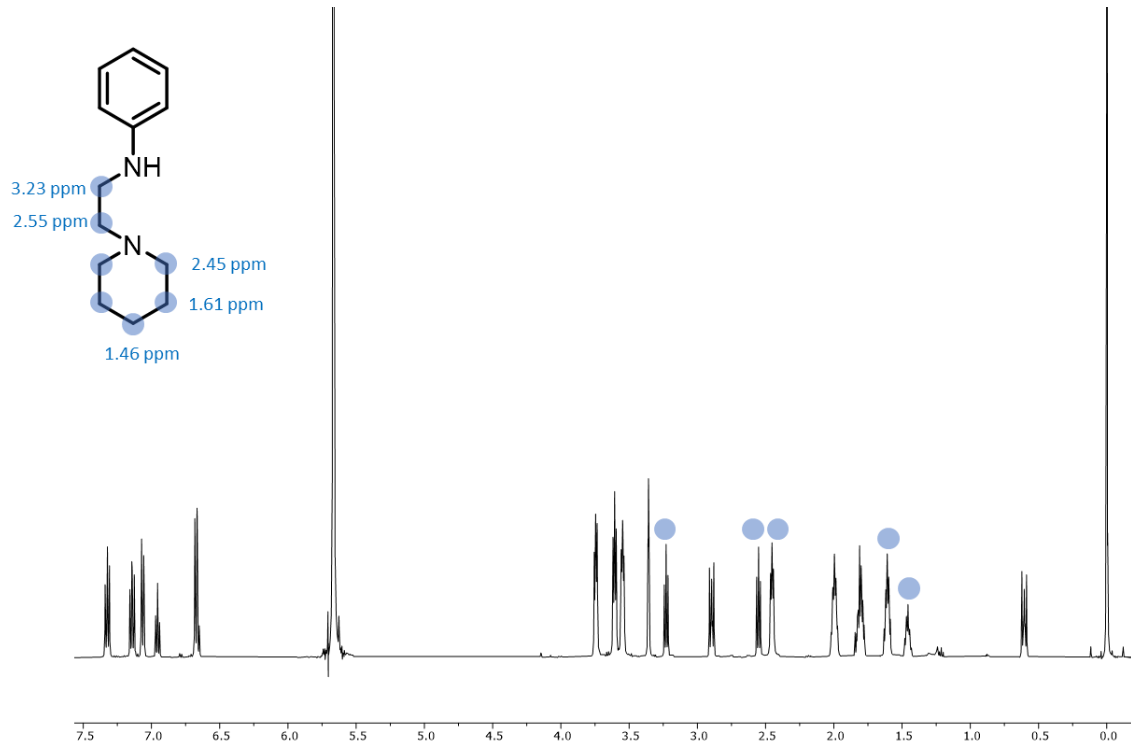

Figure S46: ^{1}H NMR spectra (700 MHz) of **5** after 240 hours dissolved in a 3M NaOD/D_2O/CD_3OD solution with internal standard (NaDSS) at 80 °C, degradation product assigned.

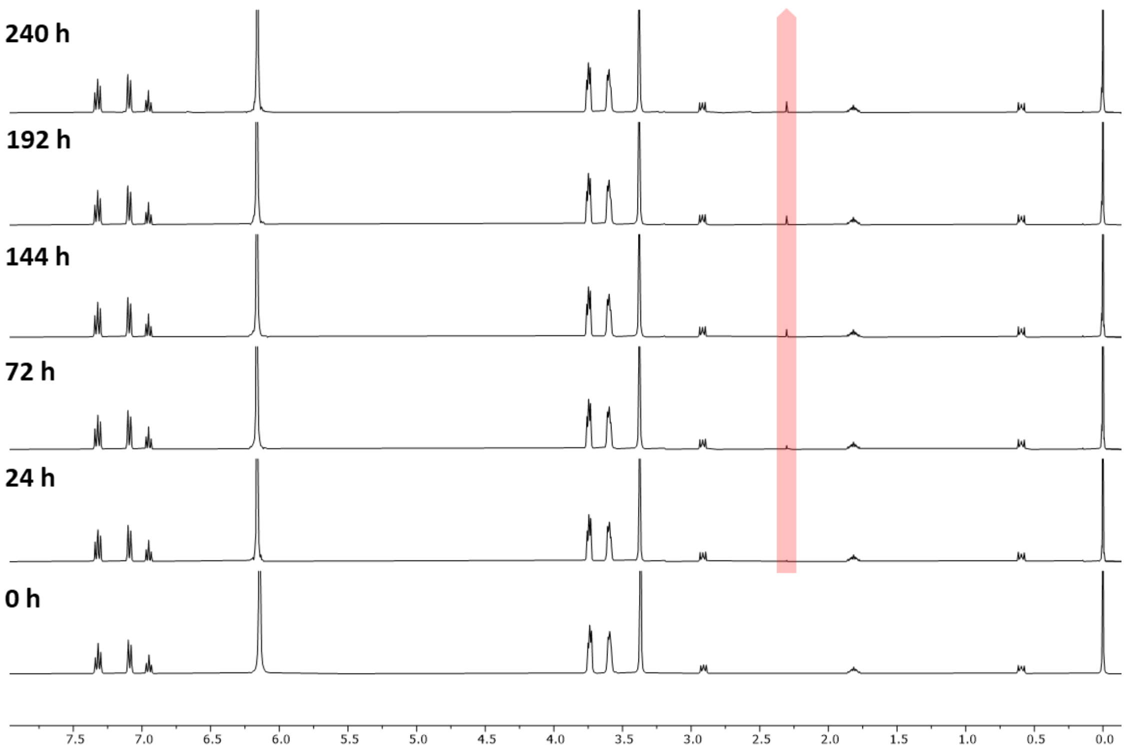

Figure S47: ^{1}H NMR spectra (400MHz) of **6** over 240 hours dissolved in a 3M NaOD/D_2O/CD_3OD solution with internal standard (NaDSS) at 60 °C.

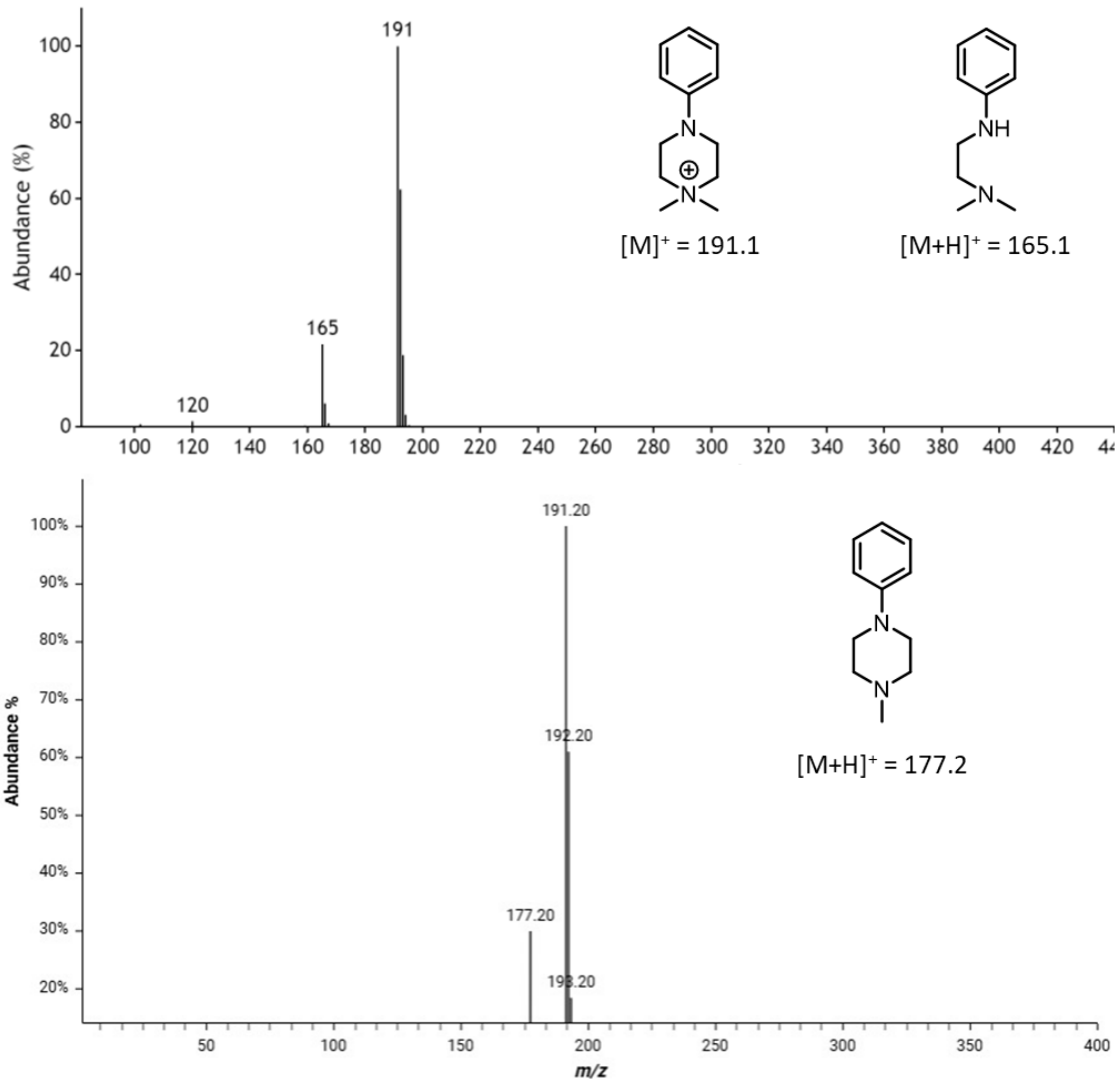

Figure S48: MS of **6** after 240 hours dissolved in a 3M NaOD/D_2O/CD_3OD solution.

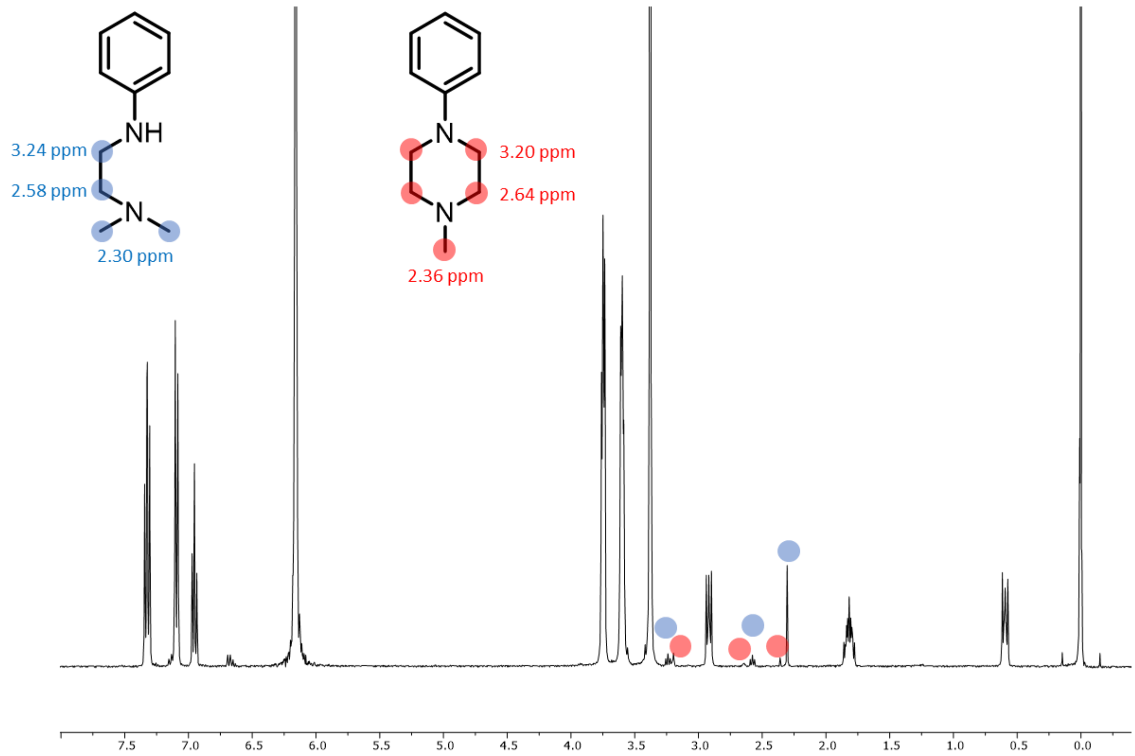

Figure S49: ^{1}H NMR spectra (400 MHz) of **6** after 240 hours dissolved in a 3M NaOD/D_2O/CD_3OD solution with internal standard (NaDSS) at 60 °C, degradation products assigned.

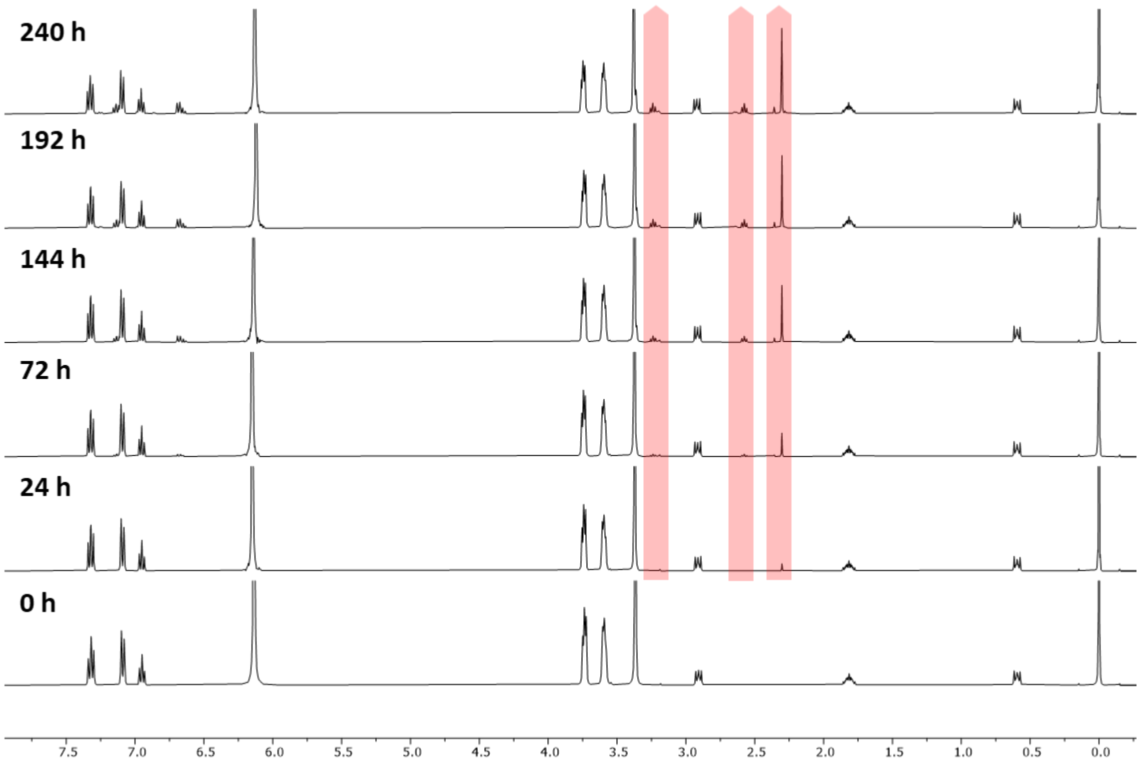

Figure S50: ^{1}H NMR spectra (400 MHz) of **6** over 240 hours dissolved in a 3M NaOD/D_2O/CD_3OD solution with internal standard (NaDSS) at 80 °C.

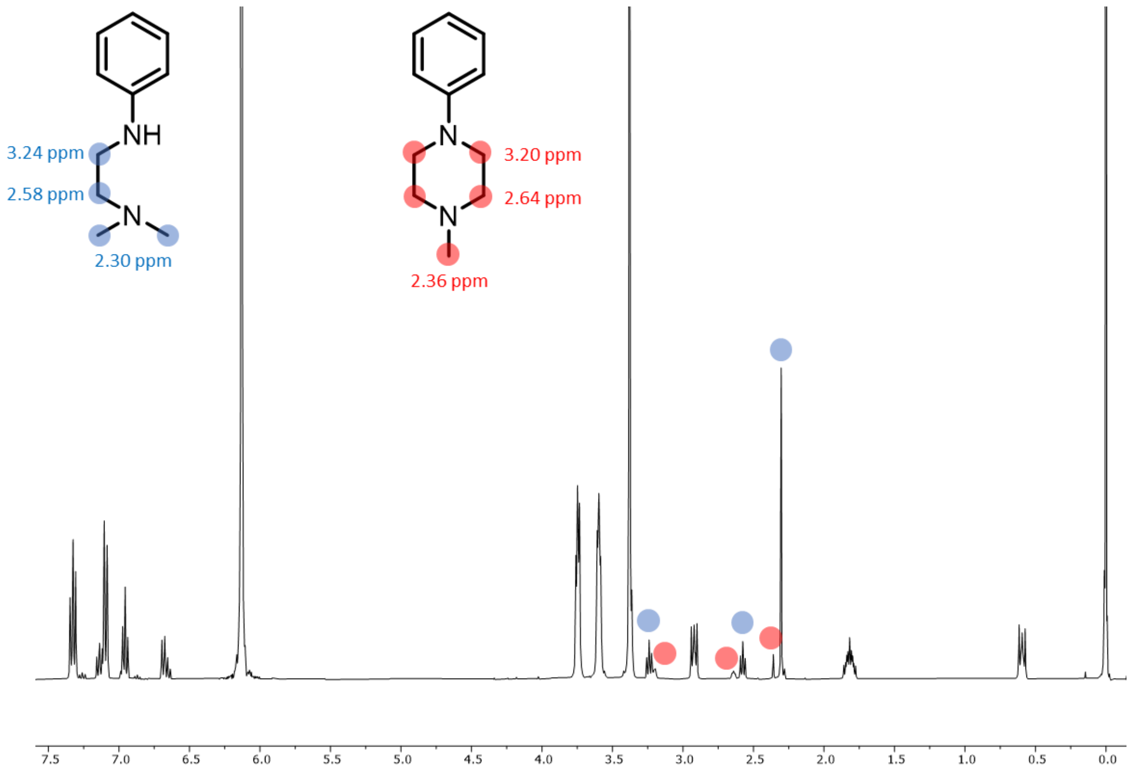

Figure S51: ^{1}H NMR spectra (400 MHz) of **6** after 240 hours dissolved in a 3M NaOD/D_2O/CD_3OD solution with an internal standard (NaDSS) at 80 °C, degradation product assigned.

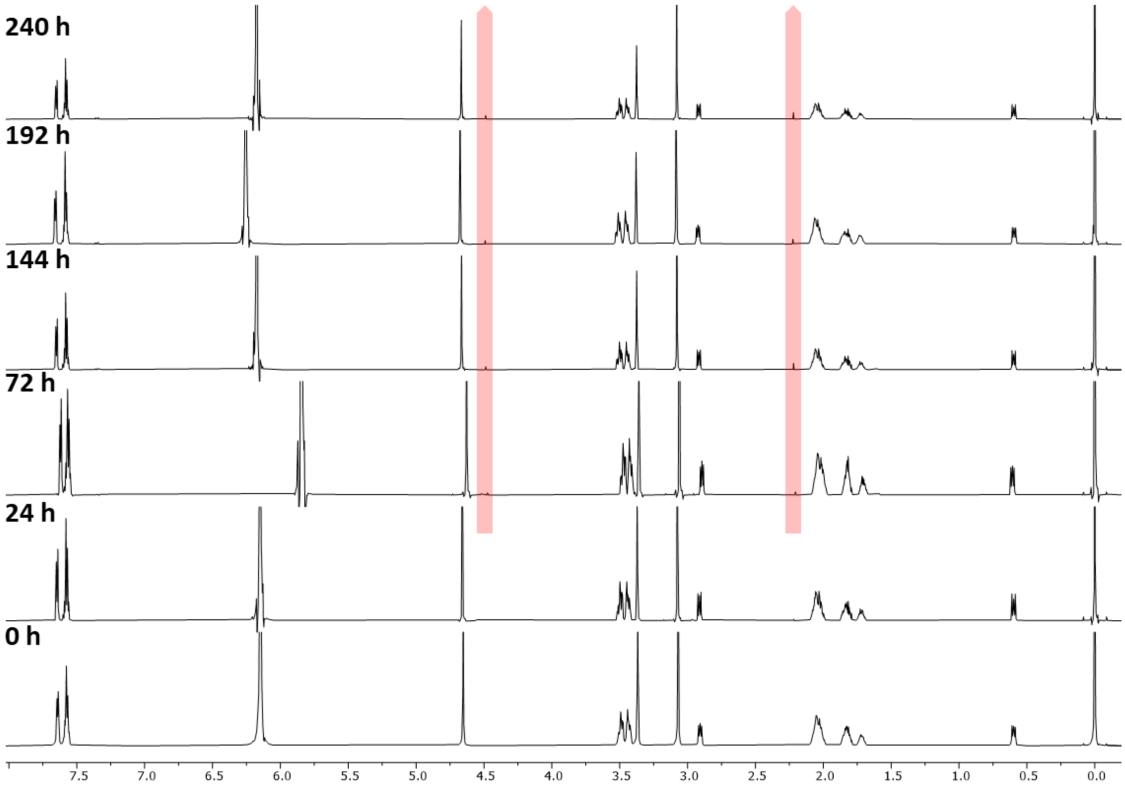

Figure S52: ^{1}H NMR spectra (700 MHz) of **7** over 240 hours dissolved in a 3M NaOD/D_2O/CD_3OD solution with an internal standard (NaDSS) at 60 °C.

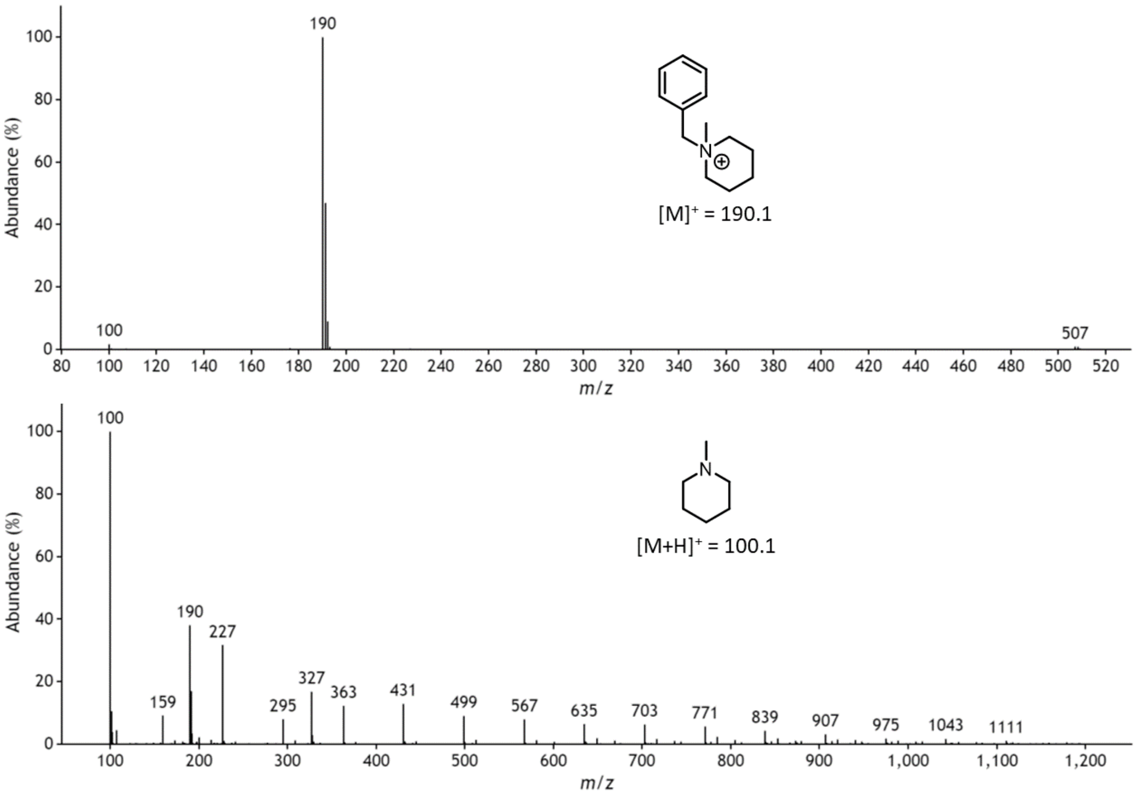

Figure S53: MS of **7** after 240 hours dissolved in a 3M NaOD/D_2O/CD_3OD solution with an internal standard (NaDSS).

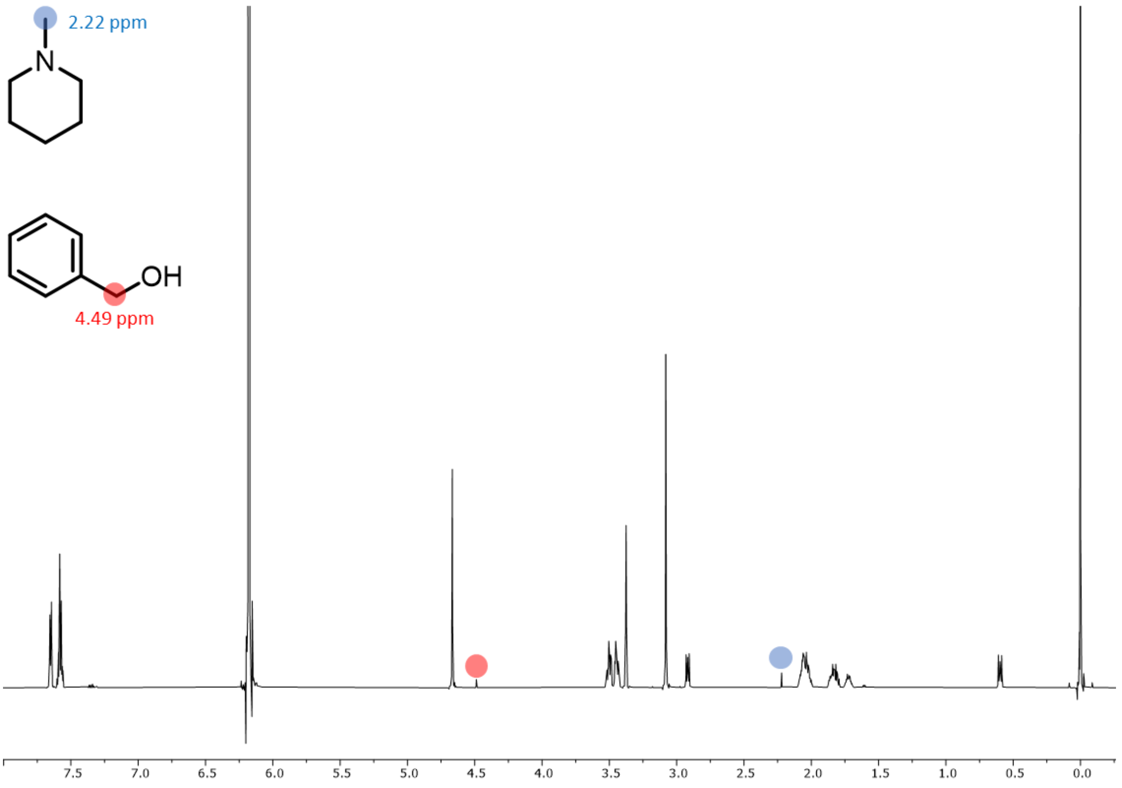

Figure S54: ^1H NMR spectra (700 MHz) of **7** after 240 hours dissolved in a 3M NaOD/D_2O/CD_3OD solution with an internal standard (NaDSS) at 60 °C, degradation product assigned.

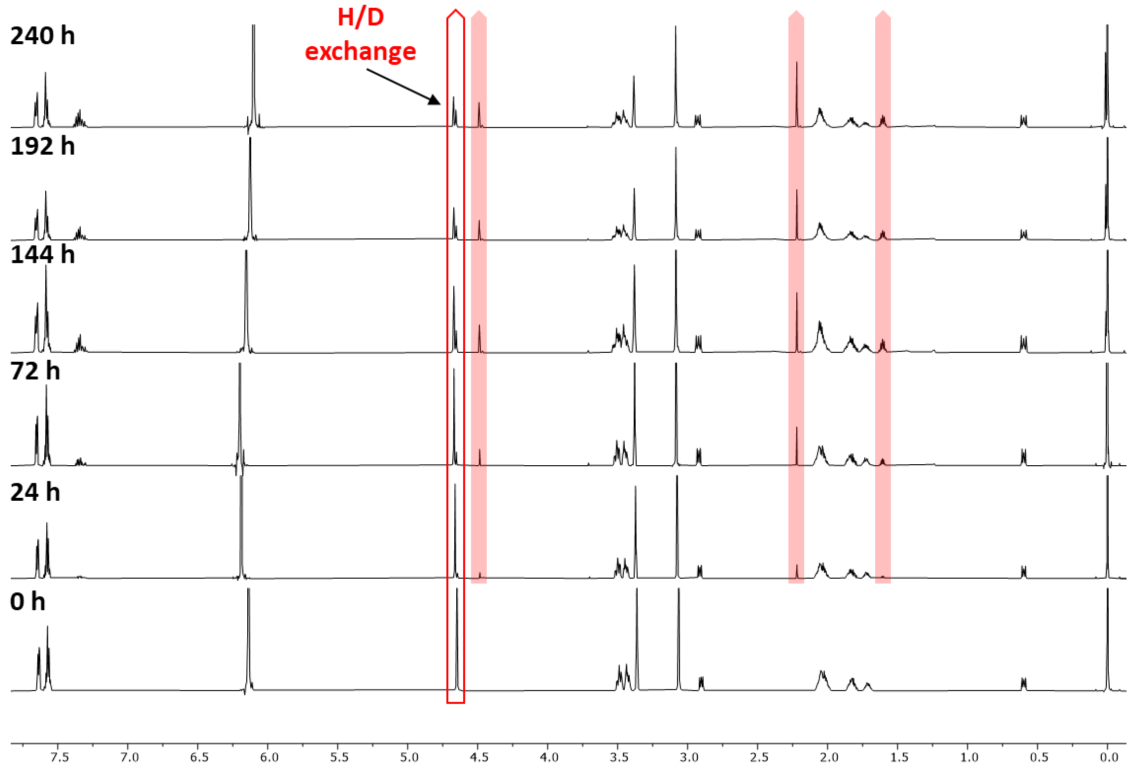

Figure S55: ^{1}H NMR spectra (700 MHz) of **7** over 240 hours dissolved in a 3M NaOD/D_2O/CD_3OD solution with internal standard (NaDSS) at 80 °C. The benzylic -CH_2 (s, 4.66 ppm) which undergoes H/D exchange is highlighted.

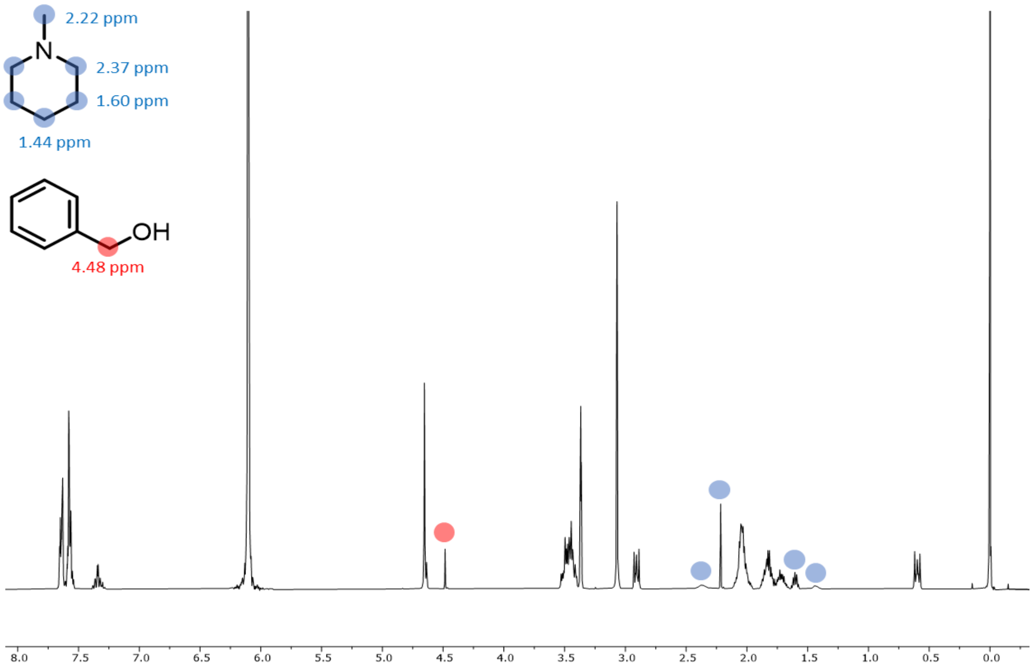

Figure S56: ^{1}H NMR spectra (700 MHz) of **7** after 240 hours dissolved in a 3M NaOD/D_2O/CD_3OD solution with internal standard (NaDSS) at 80 °C, degradation products assigned.

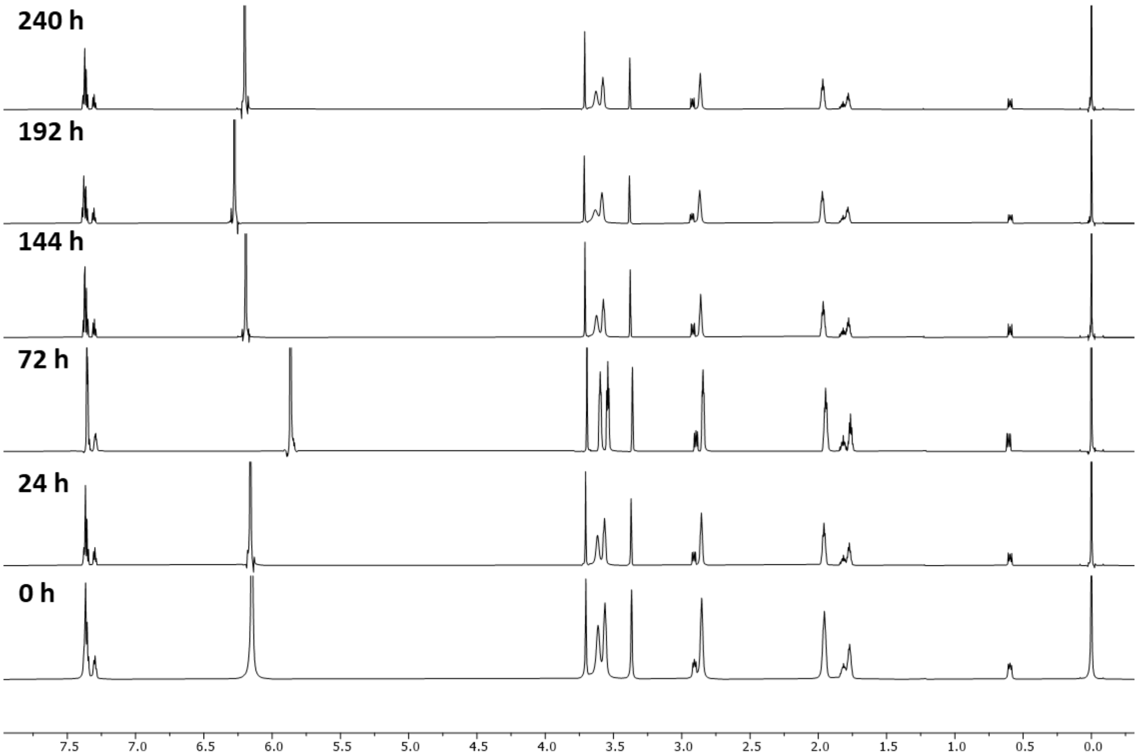

Figure S57: ^{1}H NMR spectra (700 MHz) of **8** over 240 hours dissolved in a 3M NaOD/D_2O/CD_3OD solution with internal standard (NaDSS) at 60 °C. No observable degradation.

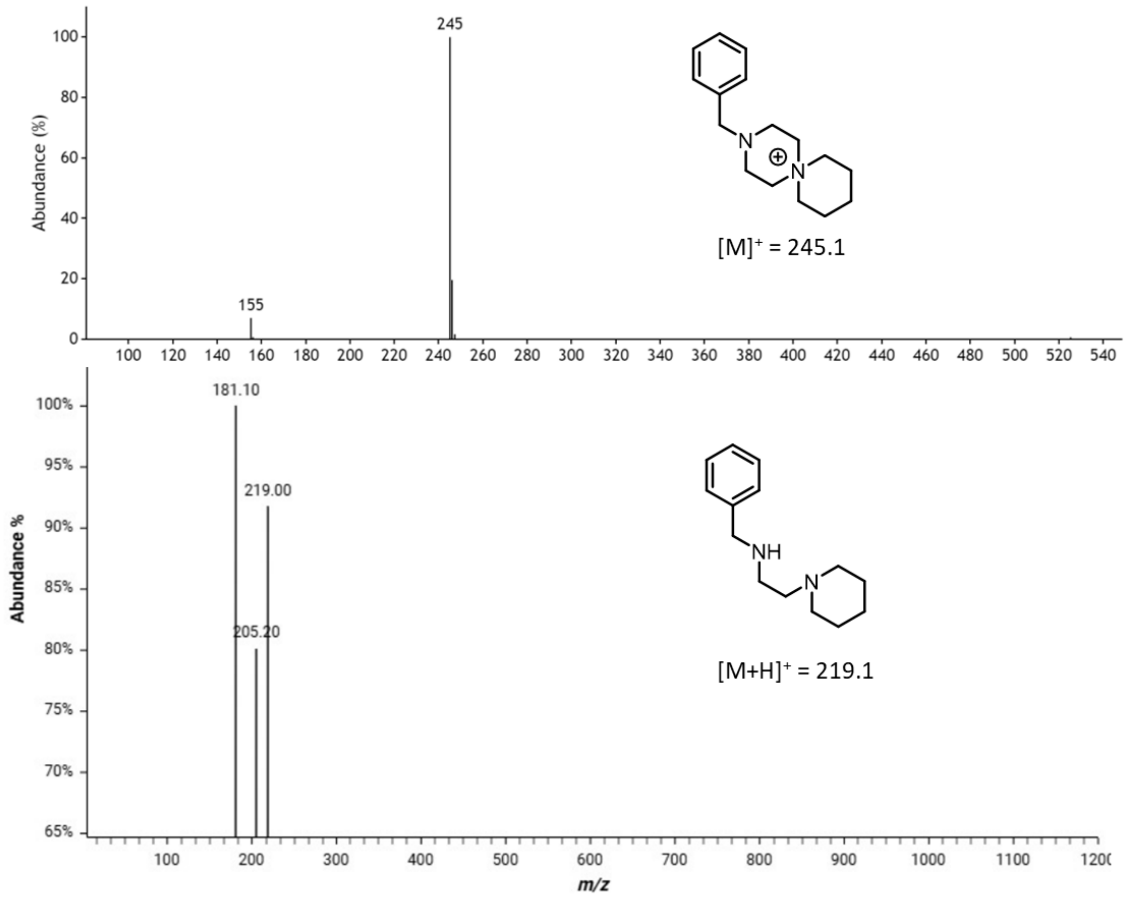

Figure S58: MS of **8** after 240 hours dissolved in a 3M NaOD/D_2O/CD_3OD solution.

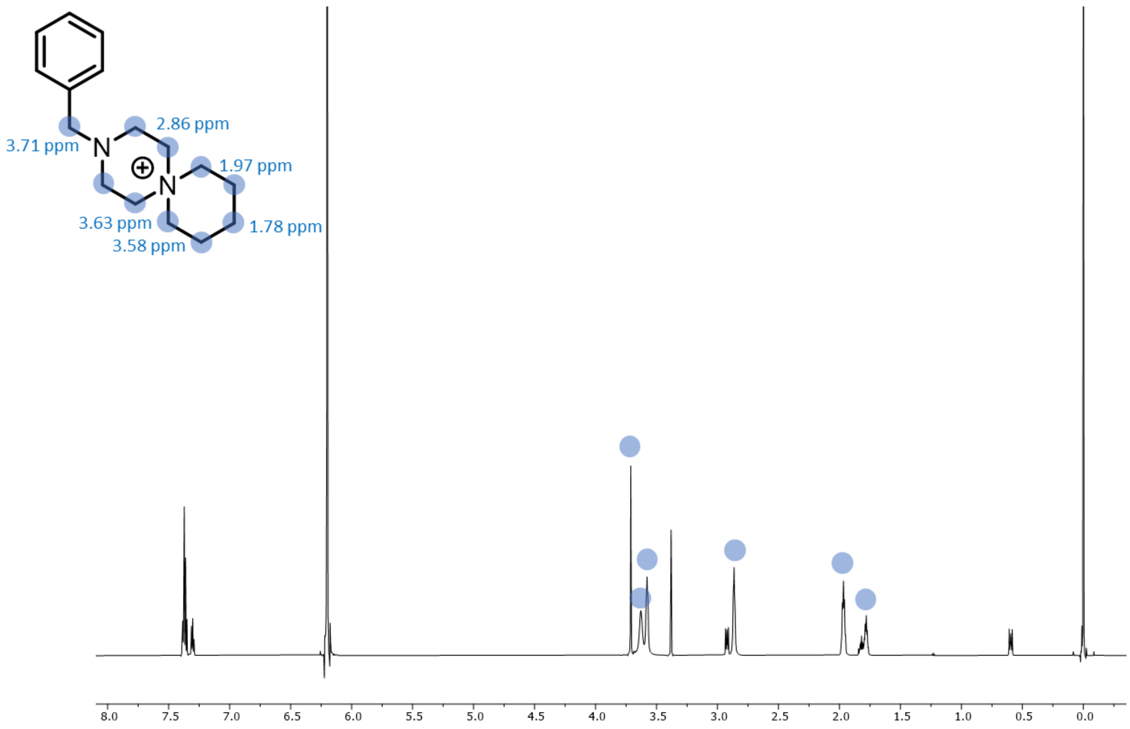

Figure S59: ^{1}H NMR spectra (700 MHz) of **8** after 240 hours dissolved in a 3M NaOD/D_2O/CD_3OD solution with internal standard (NaDSS) at 60 °C, starting cation assigned.

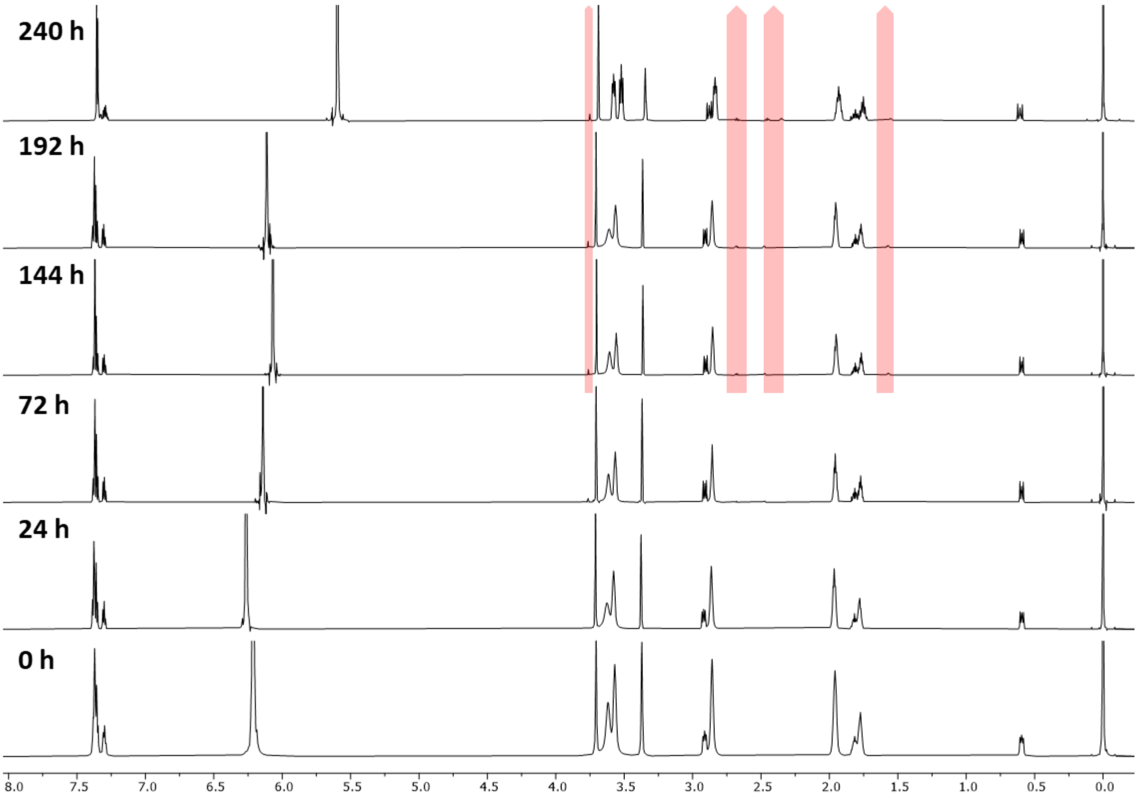

Figure S60: ^{1}H NMR spectra (500 MHz) of **8** over 240 hours dissolved in a 3M NaOD/D_2O/CD_3OD solution with internal standard (NaDSS) at 80 °C.

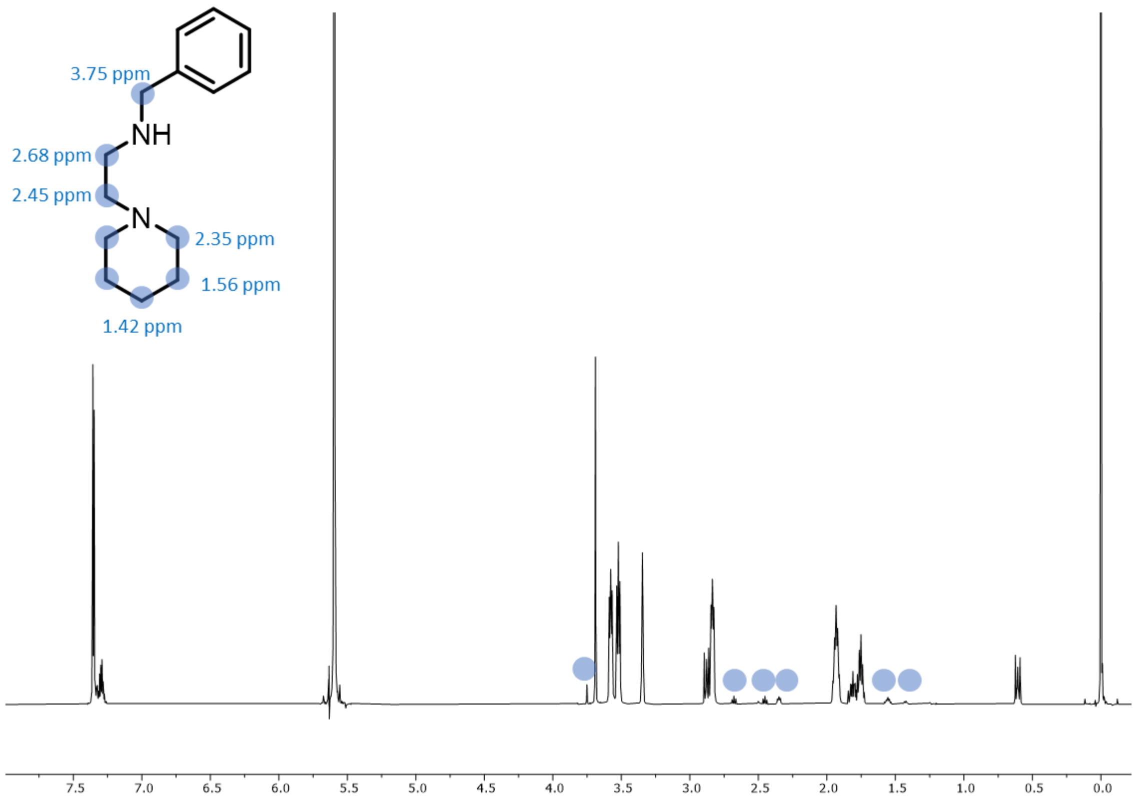

Figure S61: ^{1}H NMR spectra (500 MHz) of **8** after 240 hours dissolved in a 3M NaOD/D_2O/CD_3OD solution with internal standard (NaDSS) at 80 °C, degradation product assigned.

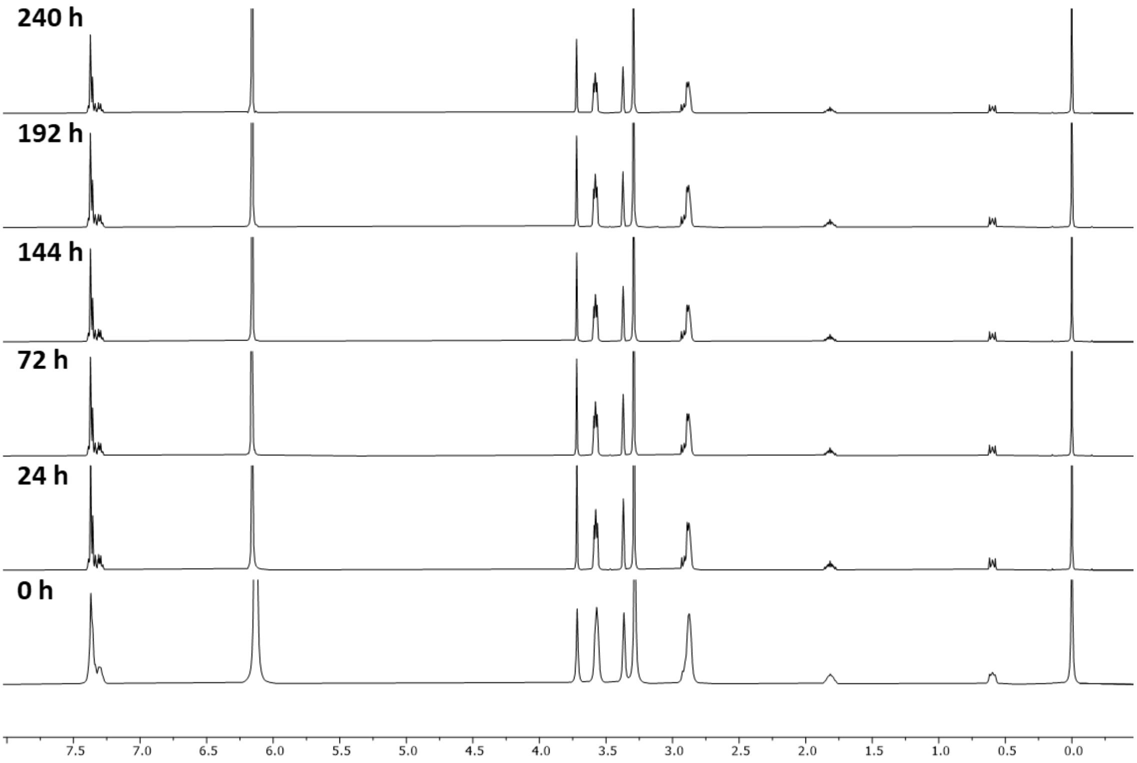

Figure S62: ^{1}H NMR spectra (400 MHz) of **9** over 240 hours dissolved in a 3M NaOD/D_2O/CD_3OD solution with internal standard (NaDSS) at 60 °C. No observable degradation.

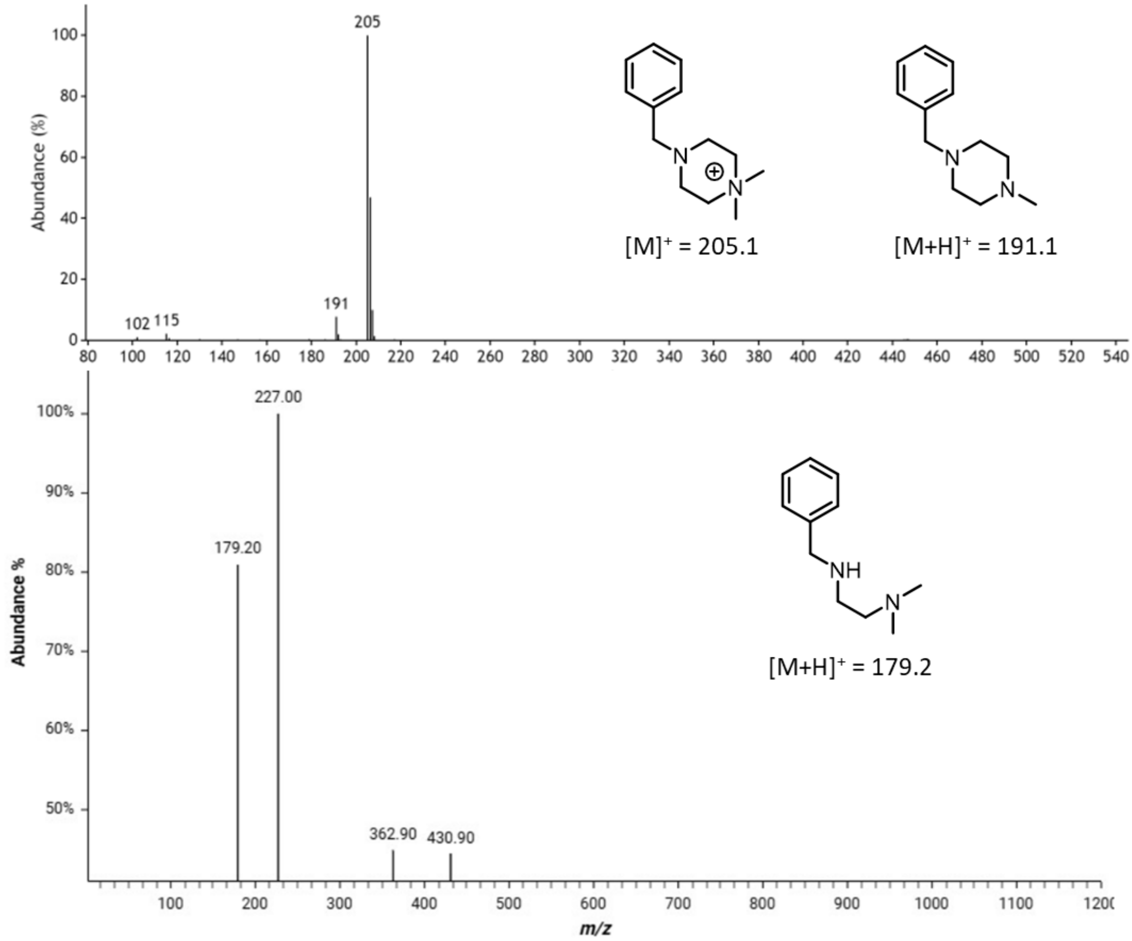

Figure S63: MS of **9** after 240 hours dissolved in a 3M NaOD/D_2O/CD_3OD solution.

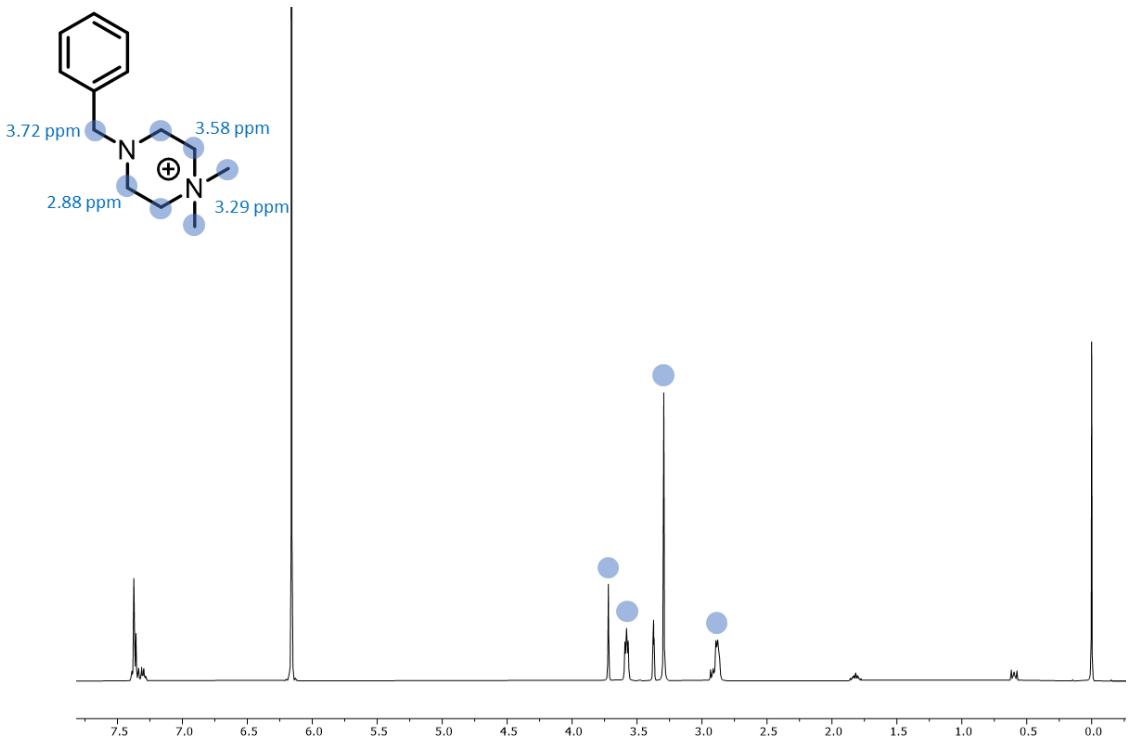

Figure S64: ^{1}H NMR spectra (400 MHz) of **9** after 240 hours dissolved in a 3M NaOD/D_2O/CD_3OD solution with internal standard (NaDSS) at 60 °C, starting cation assigned.

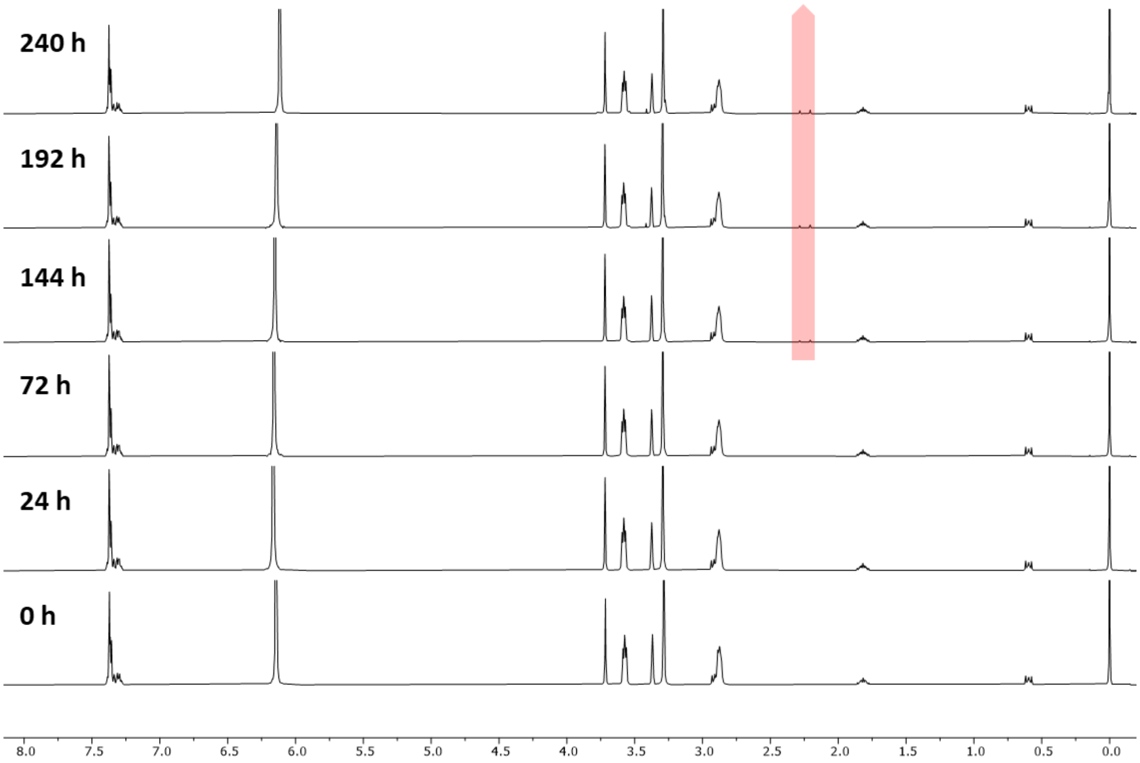

Figure S65: ^{1}H NMR spectra (400 MHz) of **9** over 240 hours dissolved in a 3M NaOD/D_2O/CD_3OD solution with internal standard (NaDSS) at 80 °C.

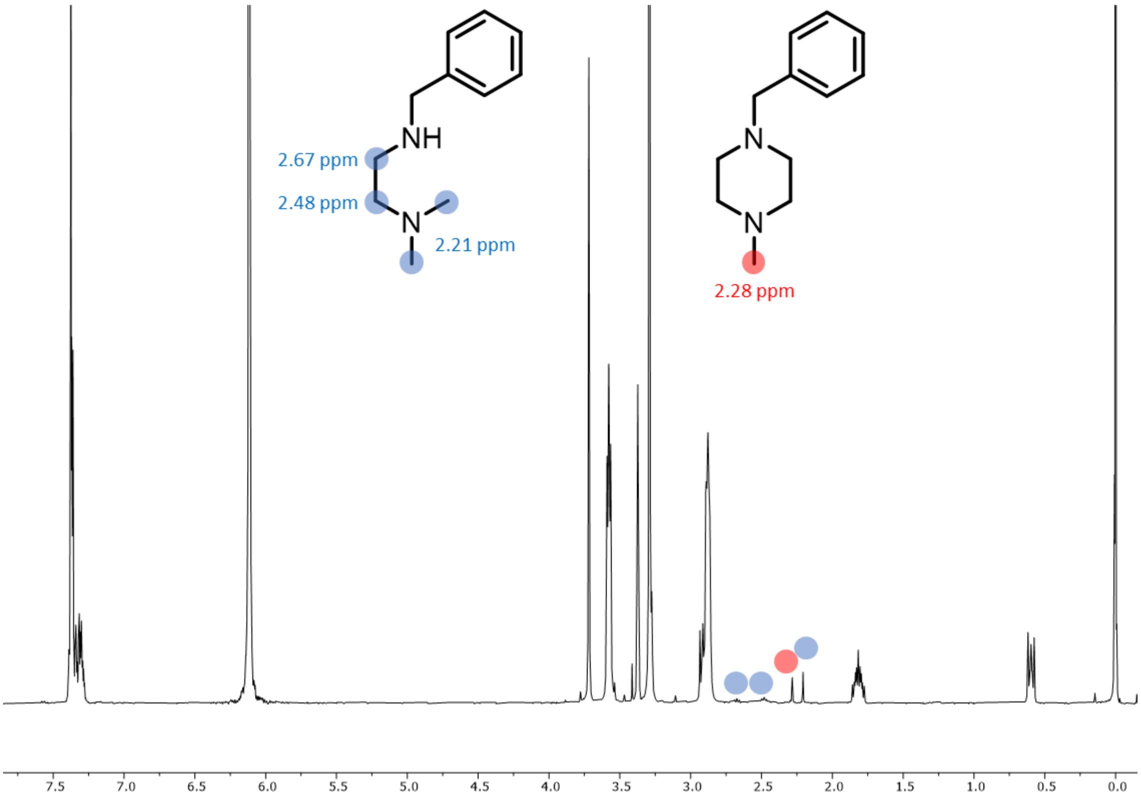

Figure S66: ^{1}H NMR spectra (400 MHz) of **9** after 240 hours dissolved in a 3M NaOD/D_2O/CD_3OD solution with internal standard (NaDSS) at 80 °C, degradation products assigned.

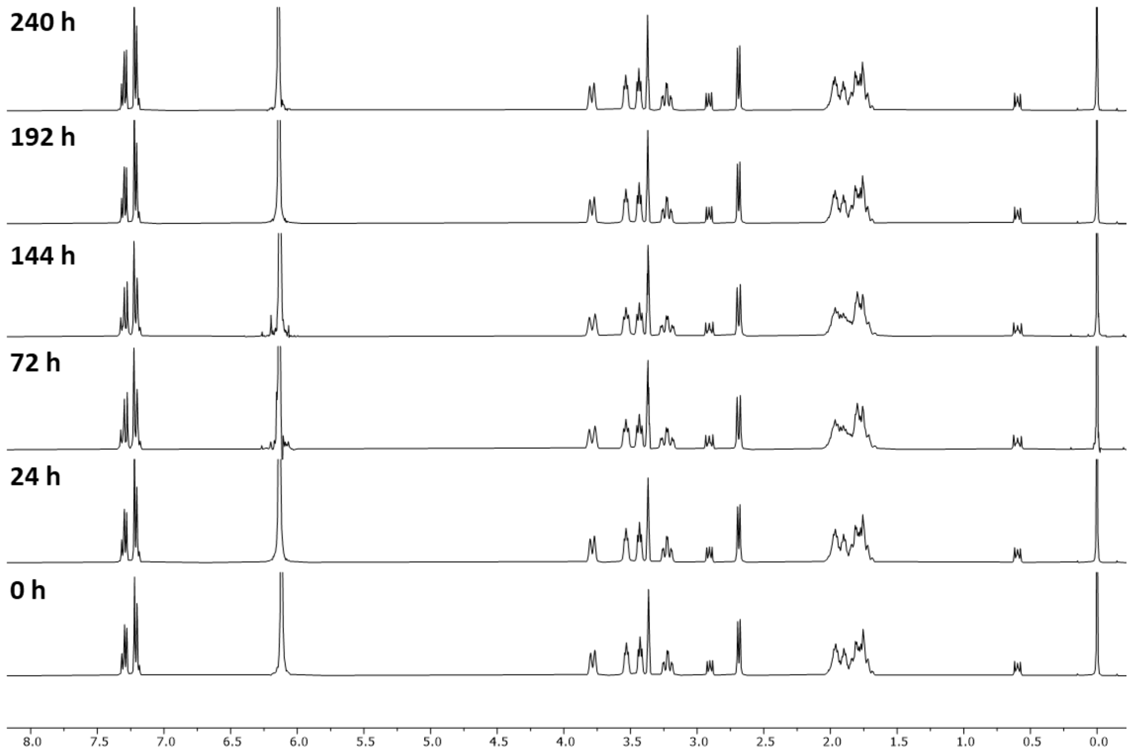

Figure S67: ^{1}H NMR spectra (400 MHz) of **10** over 240 hours dissolved in a 3M NaOD/D_2O/CD_3OD solution with internal standard (NaDSS) at 60 °C. No observable degradation.

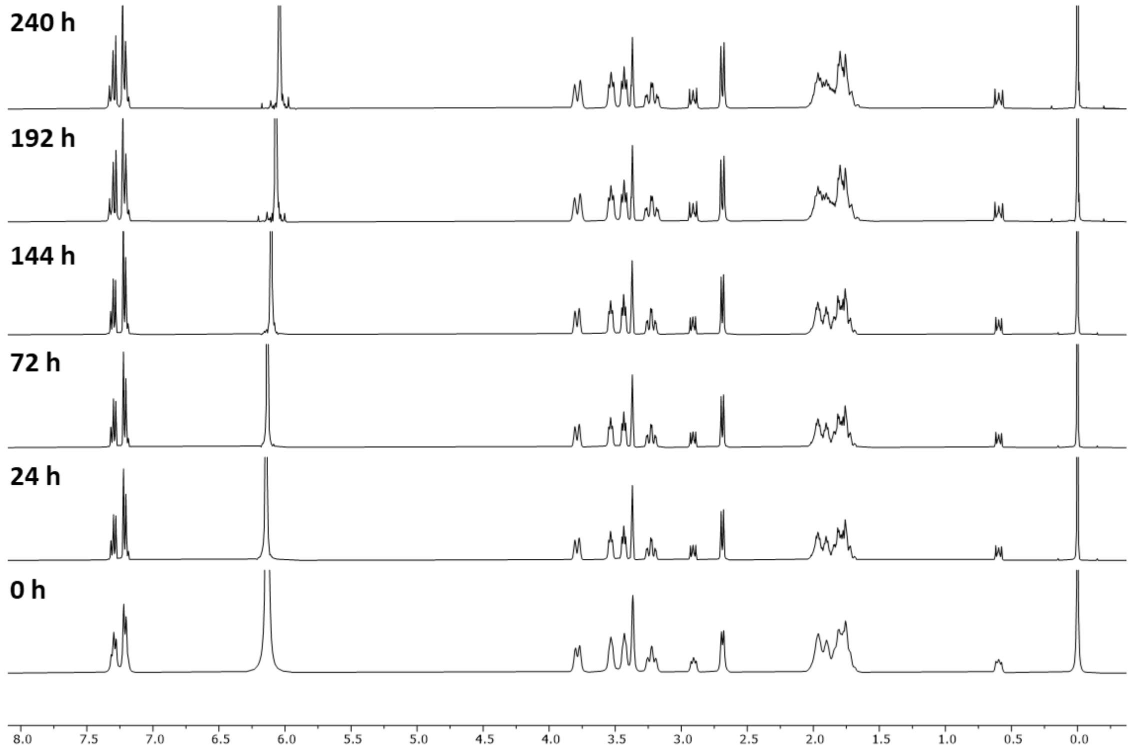

Figure S68: ^{1}H NMR spectra (400 MHz) of **10** over 240 hours dissolved in a 3M NaOD/D_2O/CD_3OD solution with internal standardC. No observable degradation.

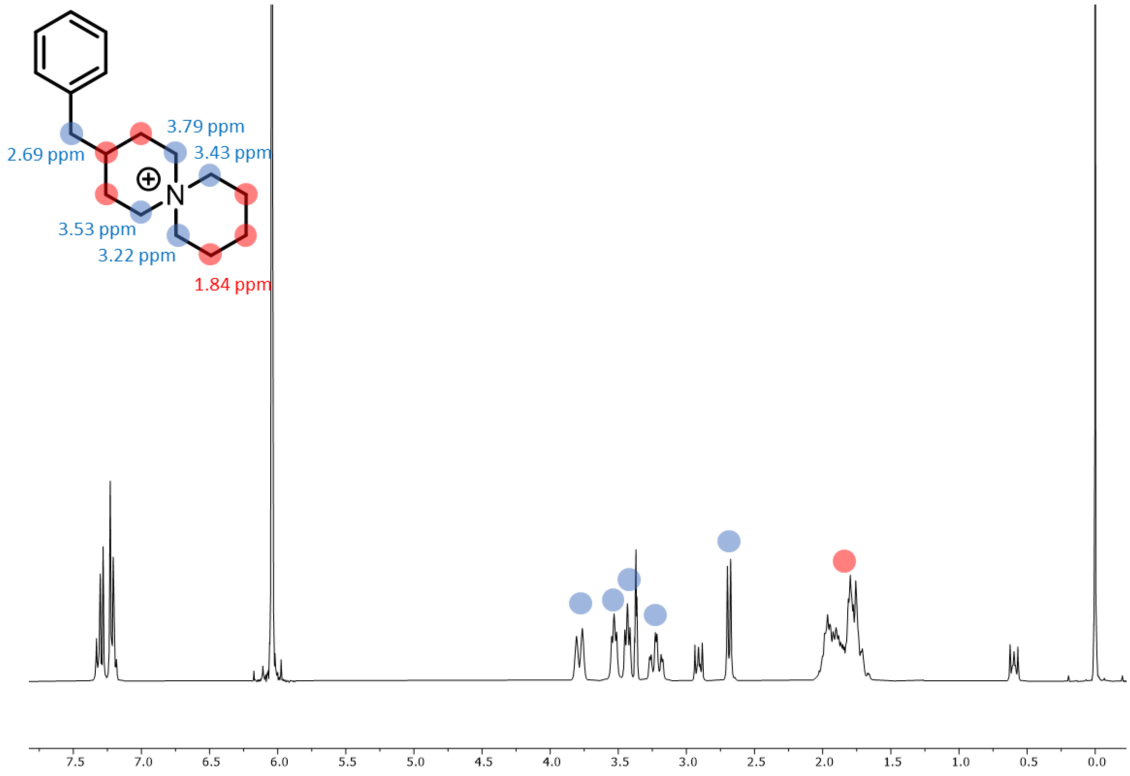

Figure S69: ^{1}H NMR spectra (400 MHz) of **10** after 240 hours dissolved in a 3M $NaOD/D_2O/CD_3OD$ solution with internal standard (NaDSS) at 80 °C, starting cation assigned.

Temp (°C)	Head group	Chemical shift δ of peak integrated (ppm)		Relative integral Area				Ratio of integrals		% change between 0 h and 240 h
		Peak X	Peak Y	X_0	Y_0	X_1	Y_1	X_0/Y_0	X_1/Y_1	
60	1	2.06	7.64	2.20	1.08	1.75	0.88	2.03	1.99	2.15
60	2	4.33	7.62	2.54	1.29	0.43	0.22	1.97	1.93	2.08
60	3	4.52	7.68	2.26	1.14	0.99	0.51	1.99	1.96	1.55
60	4	3.75	7.64	9.78	1.14	7.81	0.92	8.58	8.54	0.51
60	5	3.79	6.94	3.97	1.02	3.62	0.93	3.91	3.89	0.62
80	5	3.79	6.94	3.76	1.04	1.92	0.56	3.61	3.46	4.19
60	6	3.74	6.95	4.39	1.15	4.18	1.11	3.83	3.78	1.22
80	6	3.59	6.95	4.21	1.09	3.31	0.88	3.88	3.78	2.51
60	7	3.70	7.64	2.75	1.84	2.96	1.98	1.49	1.49	0.14
80	7	2.03	7.64	4.73	2.26	3.75	1.78	2.10	2.11	-0.44
80	7	2.03	7.64	2.14	2.26	0.65	1.78	0.95	0.37	61.52
60	8	3.59	7.34	5.32	3.47	5.23	3.43	1.53	1.53	0.55
80	8	3.59	7.34	7.51	5.17	7.21	4.82	1.45	1.50	-2.88
60	9	3.57	7.33	4.43	5.89	4.32	5.58	0.75	0.77	-2.93
80	9	3.72	7.34	1.81	5.29	1.77	5.35	0.34	0.33	3.49
60	10	3.23	7.25	2.39	5.92	2.36	5.90	0.40	0.40	0.63
80	10	3.22	7.26	2.22	5.53	2.15	5.40	0.40	0.40	0.82

Table S8: Tabulated data of head group relative integral areas (RIA) at time 0 h and 240 h to demonstrate the extent of H/D exchange in each headgroup; where X_0 and Y_0 denote the RIA of a chosen cation peak and aromatic peak at time 0 h respectively, and X_1 and Y_1 denote the RIA of a chosen cation peak and aromatic peak at time 240 h respectively. The percentage change between 0 h and 240 h is calculated using Equation 1 below and has an associated error of ± 2-3%:

$$(1)\ \%\ \text{change} = 100 - ((X_1/Y_1)/(X_0/Y_0) \times 100)$$

The benzylic -CH_2 group of **7** is added and highlighted (grey) to demonstrate the rapid H/D exchange at this position. Values are rounded to two decimal places.

Head Group	Time (h)	Half-life (h, ±8–10%)		% cation remaining	
		60 °C	80 °C	60 °C	80 °C
**1**	20	54	-	77	-
**2**	20	9	-	22	-
**3**	20	20	-	50	-
**4**	20	57	-	76	-
**5**	240	2593	289	93	56
**6**	240	2512	355	93	62
**7**	240	2702	236	94	49
**8**	240	14363	2595	98	93
**9**	240	15927	2309	99	92
**10**	240	18785	7960	99	96

Table S9. Headgroup Alkaline Stability Study: Summary of half-lives and % cation remaining as a function of time**.** Each calculated half-life is based on data from three separate measurements and has an associated error of ±8–10%.

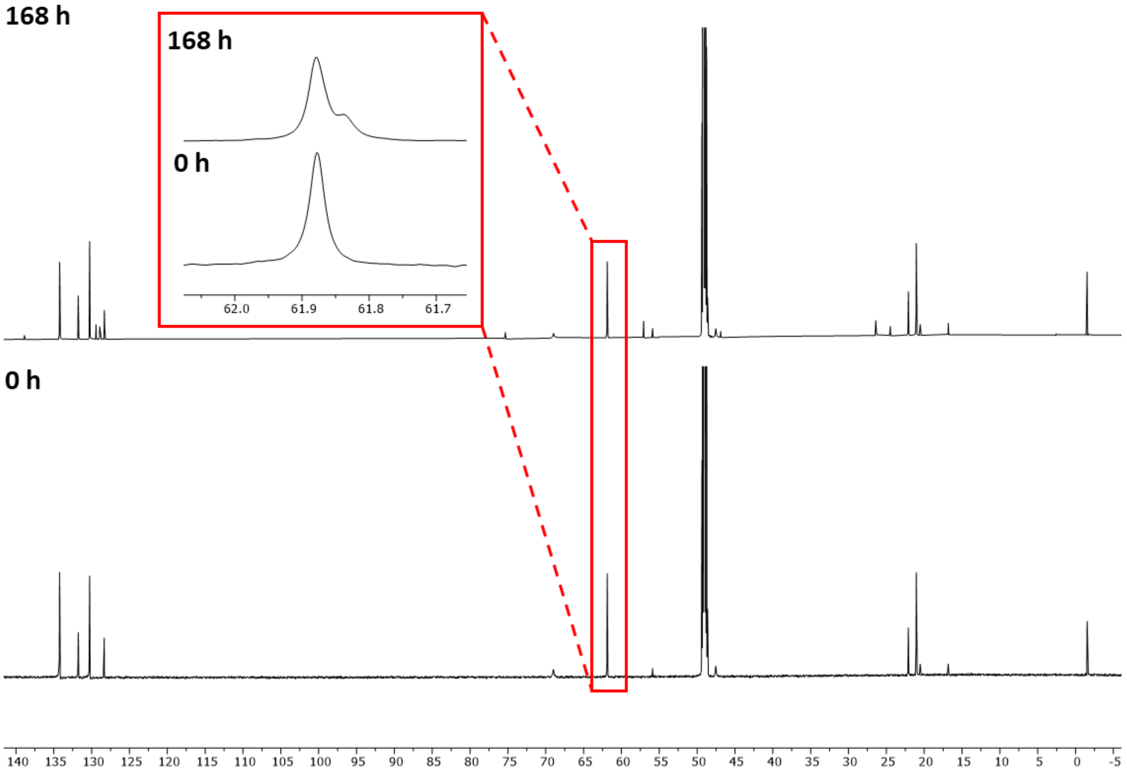

Figure S70: $^{13}C\{^{1}H\}$ NMR of **7** at 0 h and 168 h, dissolved in 3M NaOD/D_2O/CD_3OD solution with internal standard (NaDSS) and heated at 60 °C. The red box highlights the benzylic -CH_2 group which undergoes rapid H/D exchange. After 7 days, there is an observable second peak which corresponds to the -CHD isotopomer.

Computational study of head groups 1 and 7

Hydration Number	Degradation Mechanism	Initial State Energy ($kcalmol^{-1}$)	Transition State Energy ($kcalmol^{-1}$)	Final State Energy ($kcalmol^{-1}$)
0	S_N2 (MeOH)	0.00	20.08	-42.51
	S_N2 (alcohol)	0.00	22.81	-26.67
	E2 (alkene)	0.00	16.62	-36.01
1	S_N2 (MeOH)	0.00	22.04	-30.81
	S_N2 (alcohol)	0.00	24.65	-17.61
	E2 (alkene)	0.00	20.81	-26.95
2	S_N2 (MeOH)	0.00	26.83	-25.85
	S_N2 (alcohol)	0.00	28.94	-9.23
	E2 (alkene)	0.00	26.99	-19.05
3	S_N2 (MeOH)	0.00	28.31	-26.85
	S_N2 (alcohol)	0.00	29.44	-12.72
	E2 (alkene)	0.00	29.48	-21.24
4	S_N2 (MeOH)	0.00	32.28	-22.04
	S_N2 (alcohol)	0.00	34.89	-6.94
	E2 (alkene)	0.00	35.33	-17.47

Table S10: Table of calculated Gibbs free energies for headgroups **1** at 60 °C with increasing hydration number (λ = 0, 1, 2, 3, 4).

Hydration Number	Degradation Mechanism	Initial State Energy (kcalmol^{-1})	Transition State Energy (kcalmol^{-1})	Final State Energy (kcalmol^{-1})
0	S_N2 (MeOH)	0.00	24.97	-34.42
	S_N2 (alcohol)	0.00	27.94	-16.44
	E2 (alkene)	0.00	18.90	-26.80
	S_N2 (BnOH)	0.00	22.15	-25.24
1	S_N2 (MeOH)	0.00	30.58	-22.91
	S_N2 (alcohol)	0.00	33.27	-6.35
	E2 (alkene)	0.00	26.96	-18.02
	S_N2 (BnOH)	0.00	26.63	-17.32
2	S_N2 (MeOH)	0.00	33.02	-17.76
	S_N2 (alcohol)	0.00	36.50	1.33
	E2 (alkene)	0.00	31.61	-9.85
	S_N2 (BnOH)	0.00	29.84	-9.21
3	S_N2 (MeOH)	0.00	33.80	-18.76
	S_N2 (alcohol)	0.00	36.66	1.00
	E2 (alkene)	0.00	32.01	-12.03
	S_N2 (BnOH)	0.00	30.62	-10.52
4	S_N2 (MeOH)	0.00	39.97	-13.95
	S_N2 (alcohol)	0.00	44.01	3.72
	E2 (alkene)	0.00	38.35	-8.26
	S_N2 (BnOH)	0.00	36.60	-4.66

Table S11: Table of calculated Gibbs free energies for headgroups **7** at 60 °C with increasing hydration number (λ = 0, 1, 2, 3, 4).

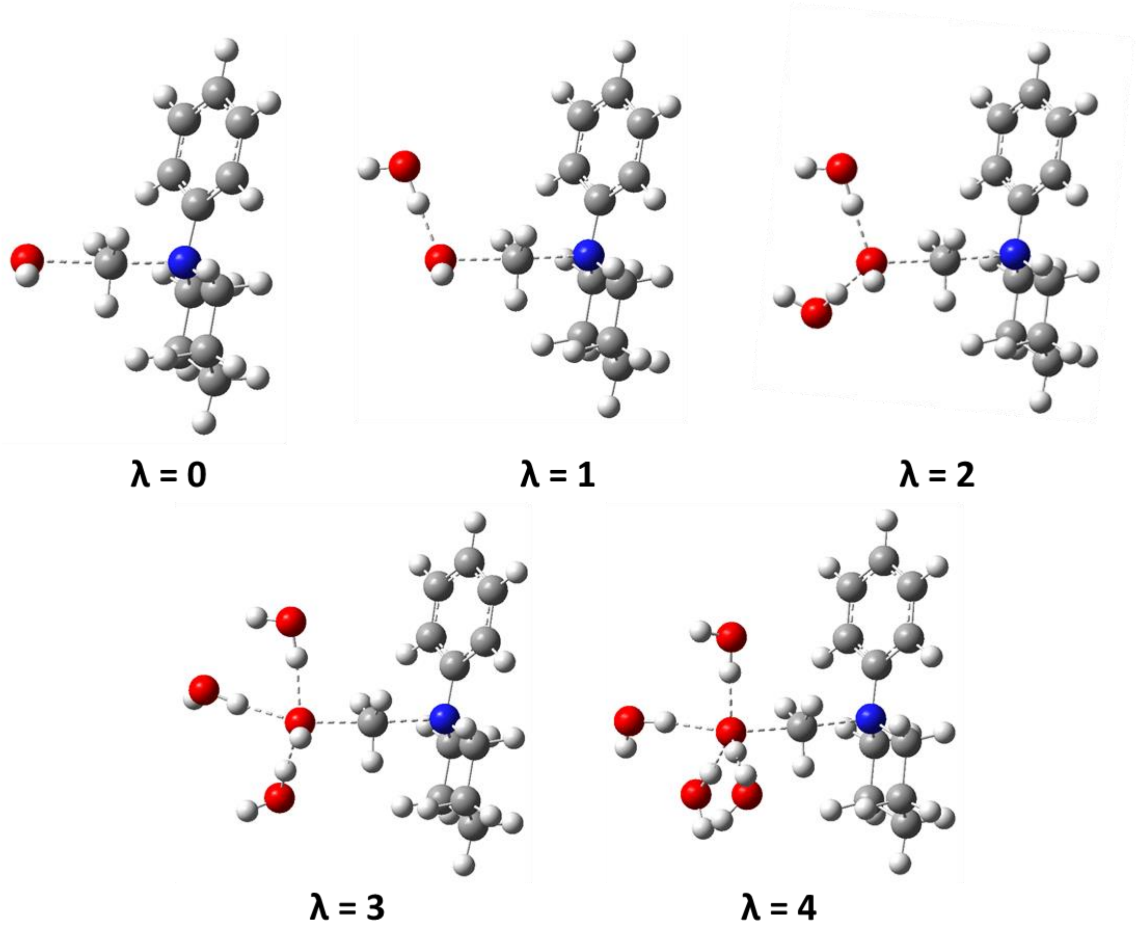

Figure S71: Calculated S_N2 demethylation transition states for **1** at hydration number 0, 1, 2, 3, 4.

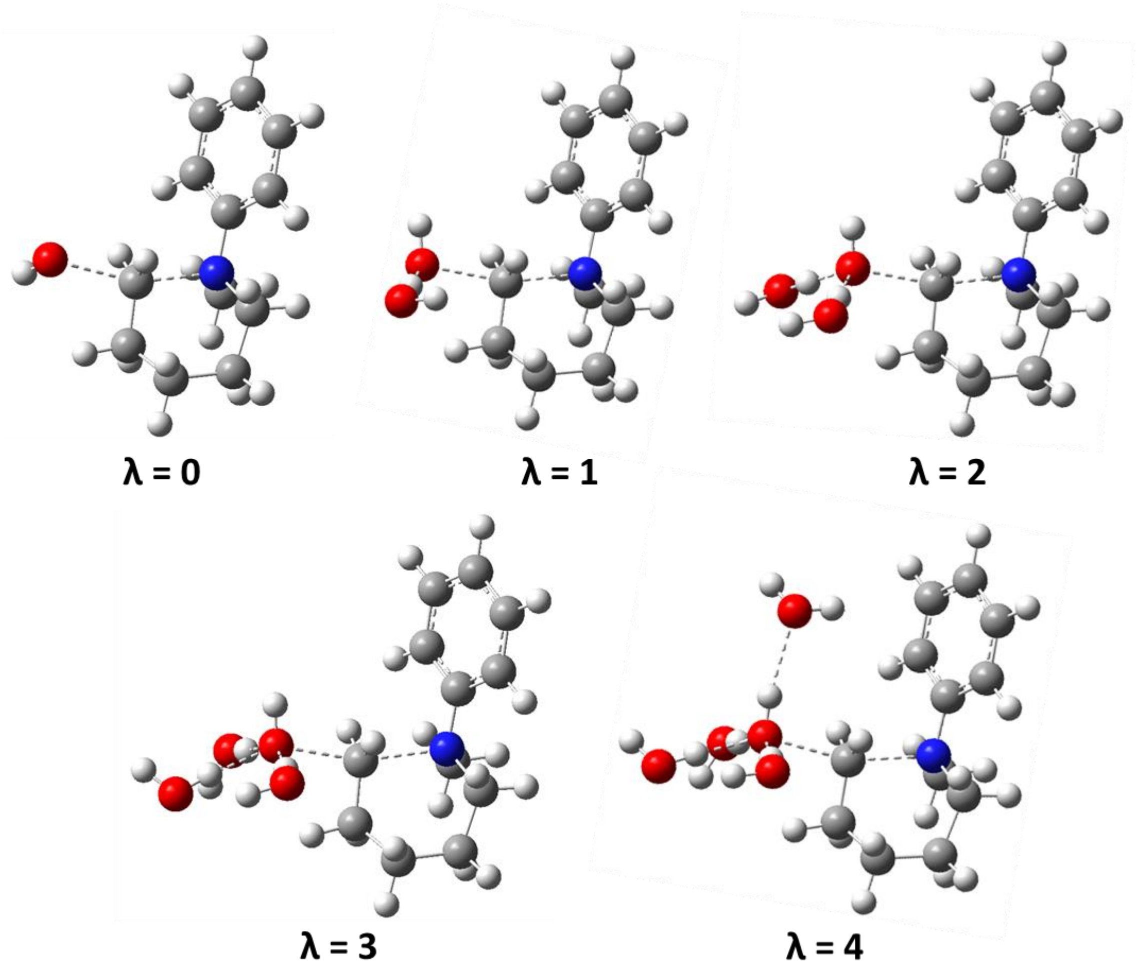

Figure S72: Calculated S_N2 ring opening transition states for **1** at hydration number 0, 1, 2, 3, 4.

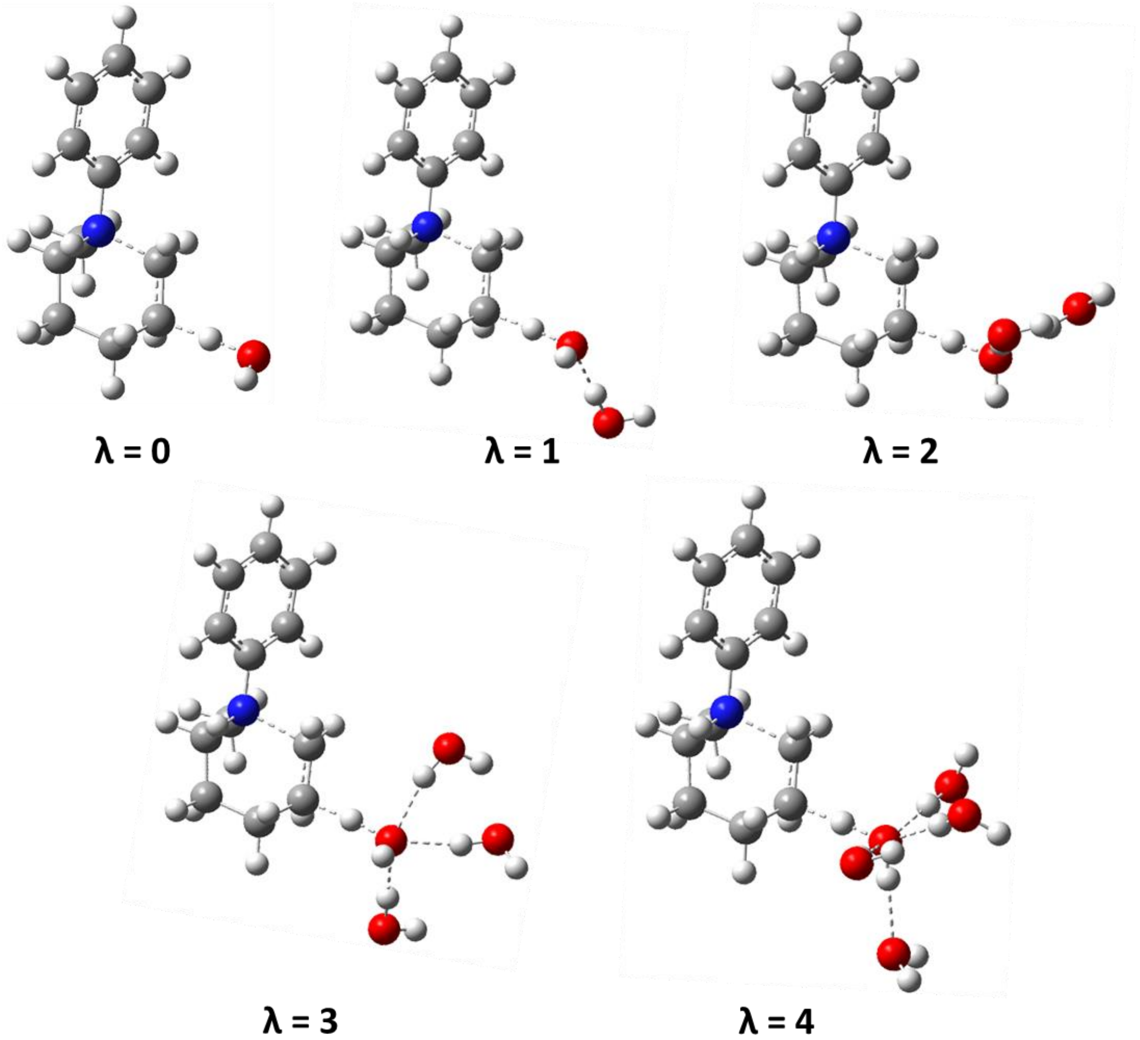

λ = 0 **λ = 1** **λ = 2**

λ = 3 **λ = 4**

Figure S73: Calculated E2 ring opening transition states for **1** at hydration number 0, 1, 2, 3, 4.

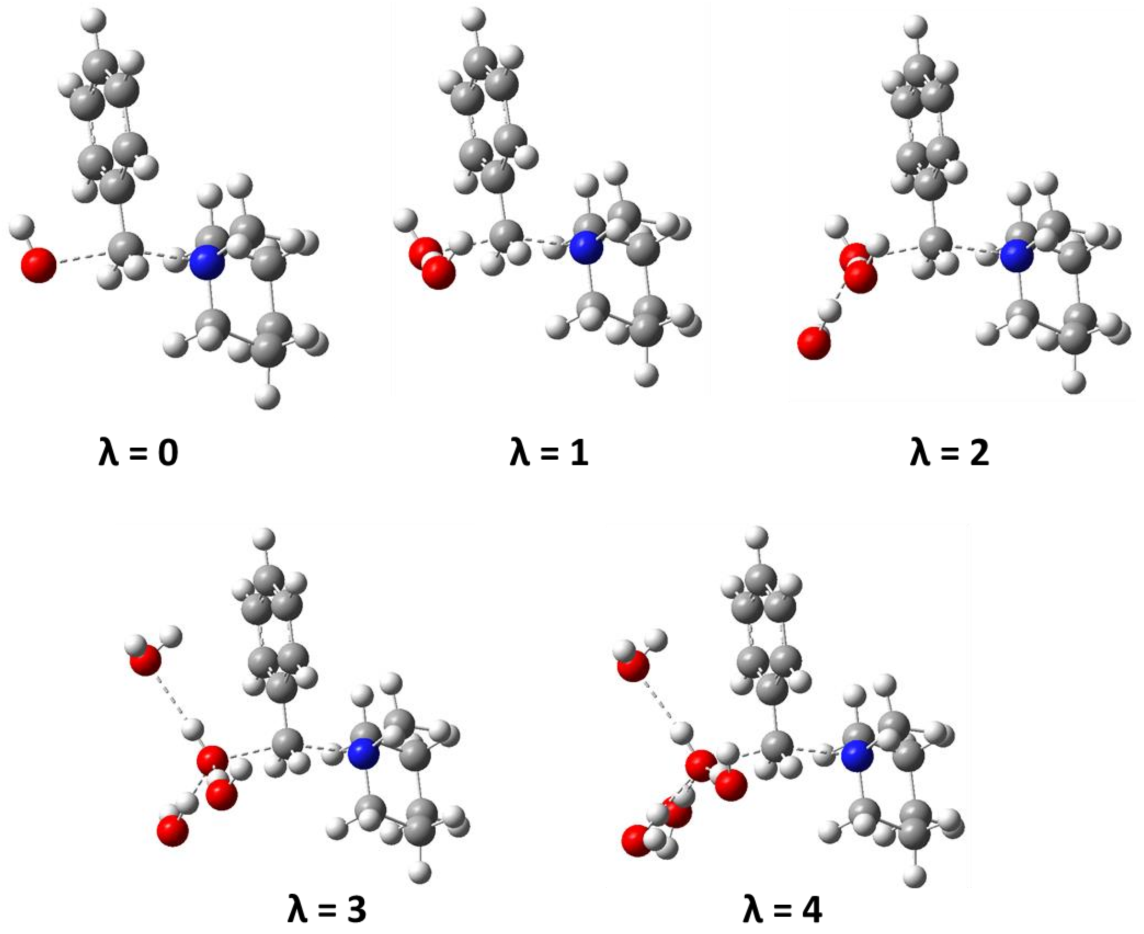

Figure S74: Calculated S_N2 debenzylation transition states for **7** at hydration number 0, 1, 2, 3, 4.

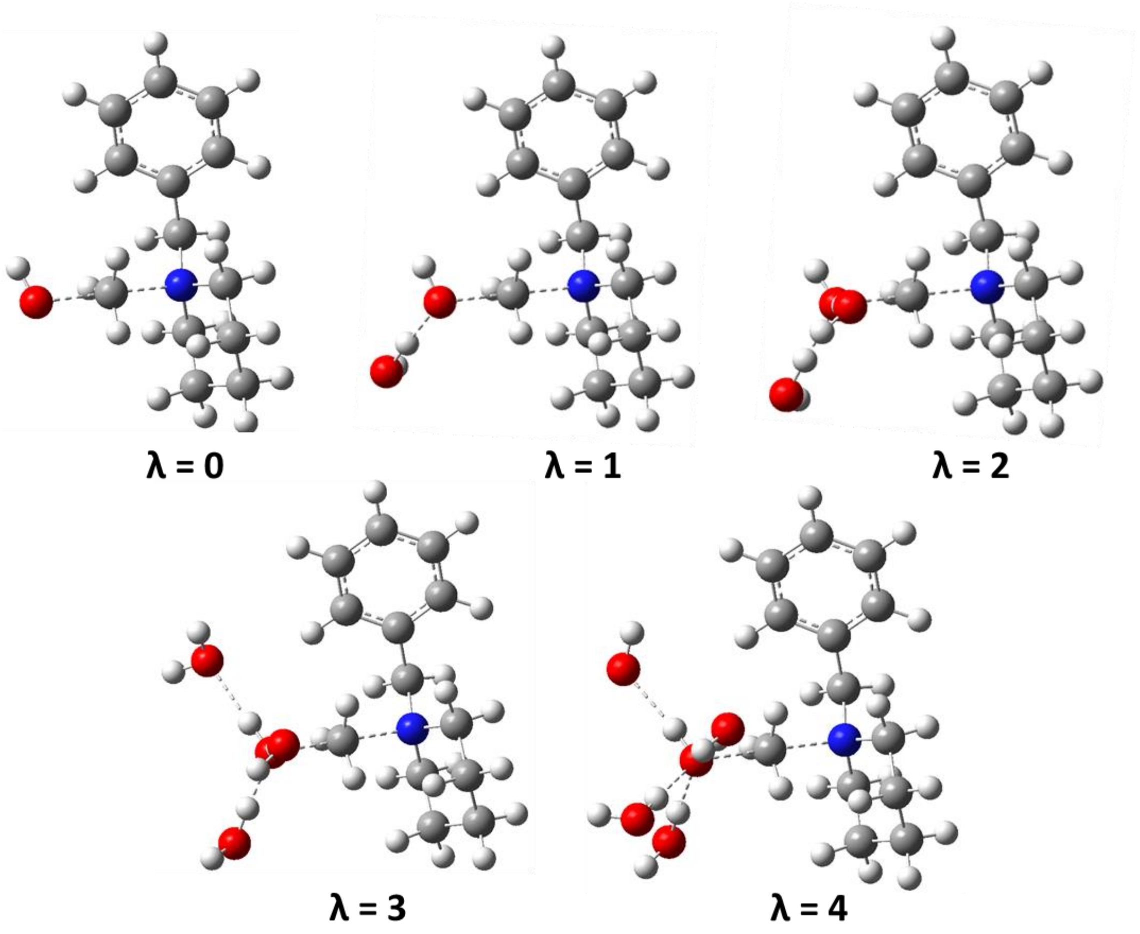

Figure S75: Calculated S_N2 demethylation transition states for **7** at hydration number 0, 1, 2, 3, 4.

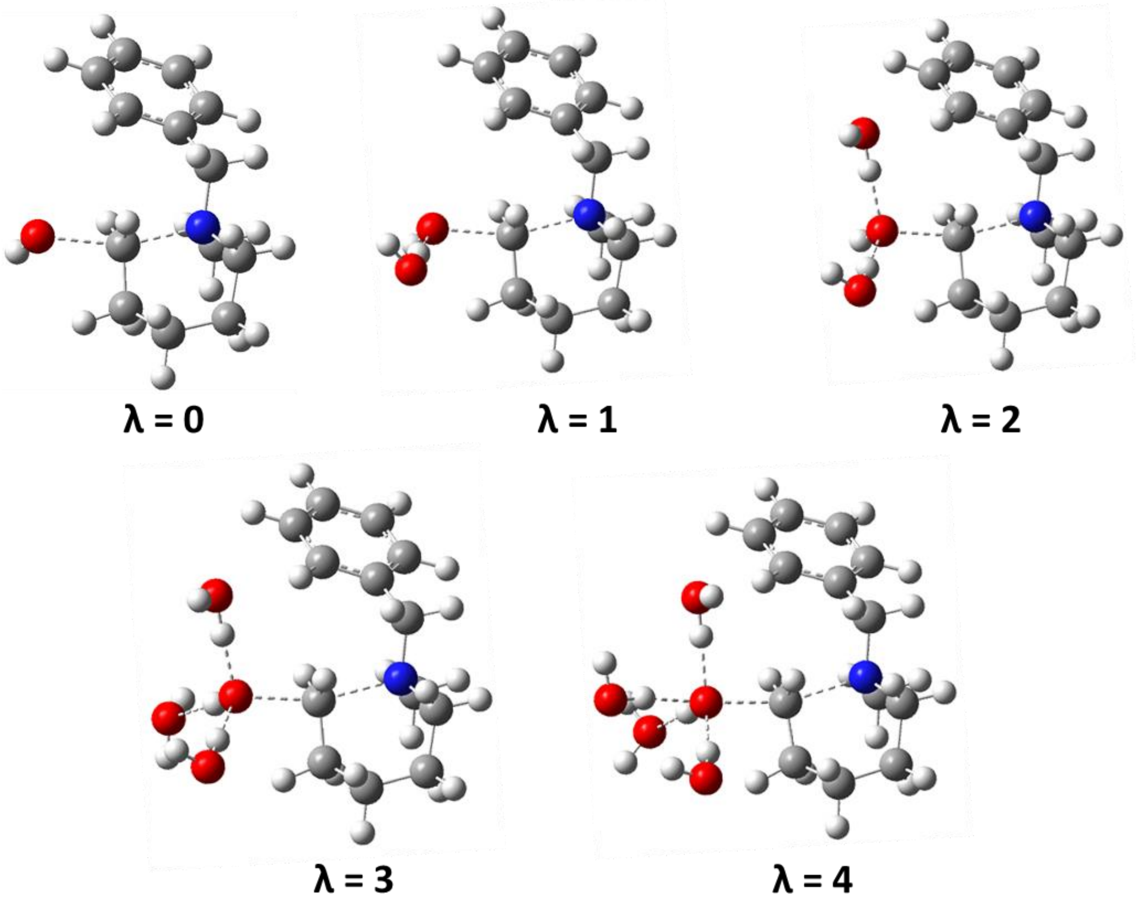

Figure S76: Calculated S_N2 ring opening transition states for **7** at hydration number 0, 1, 2, 3, 4.

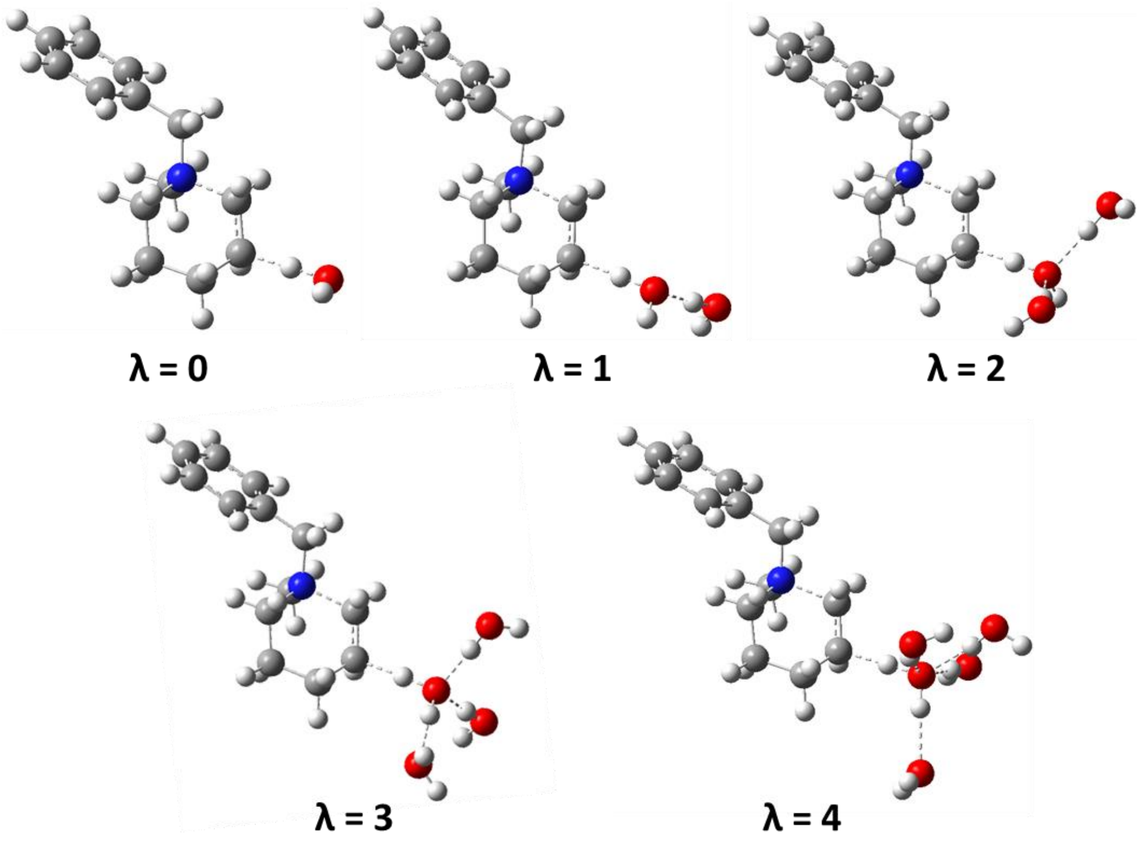

Figure S77: Calculated E2 ring opening transition states for **7** at hydration number 0, 1, 2, 3, 4.

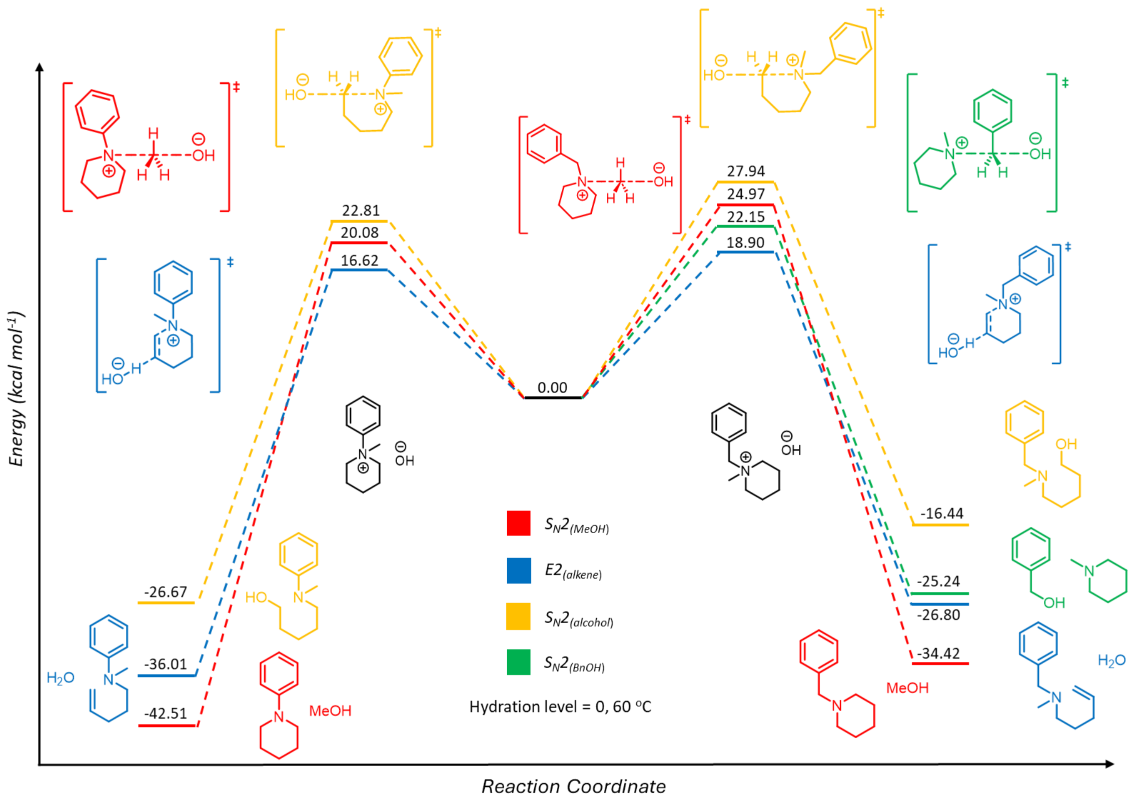

Figure S78: Gibbs free energy level diagram for **1** and **7** at 60 °C at hydration number = 0.

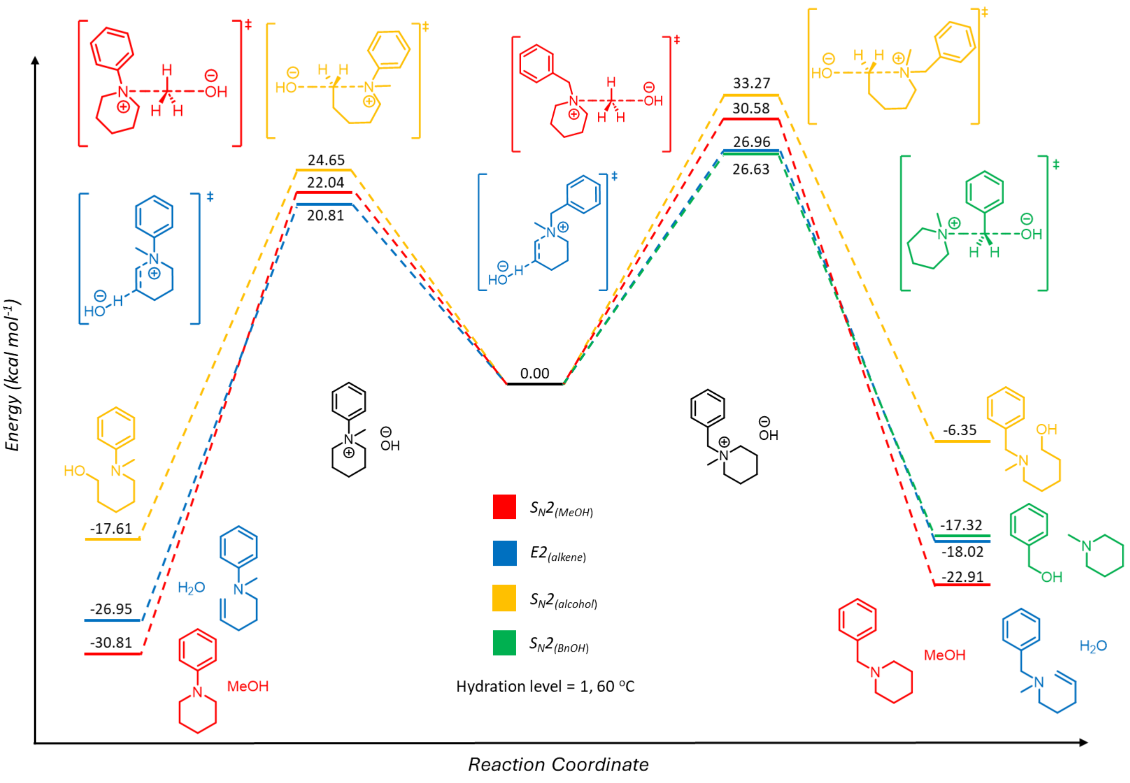

Figure S79: Gibbs free energy level diagram for **1** and **7** at 60 °C at hydration number = 1.

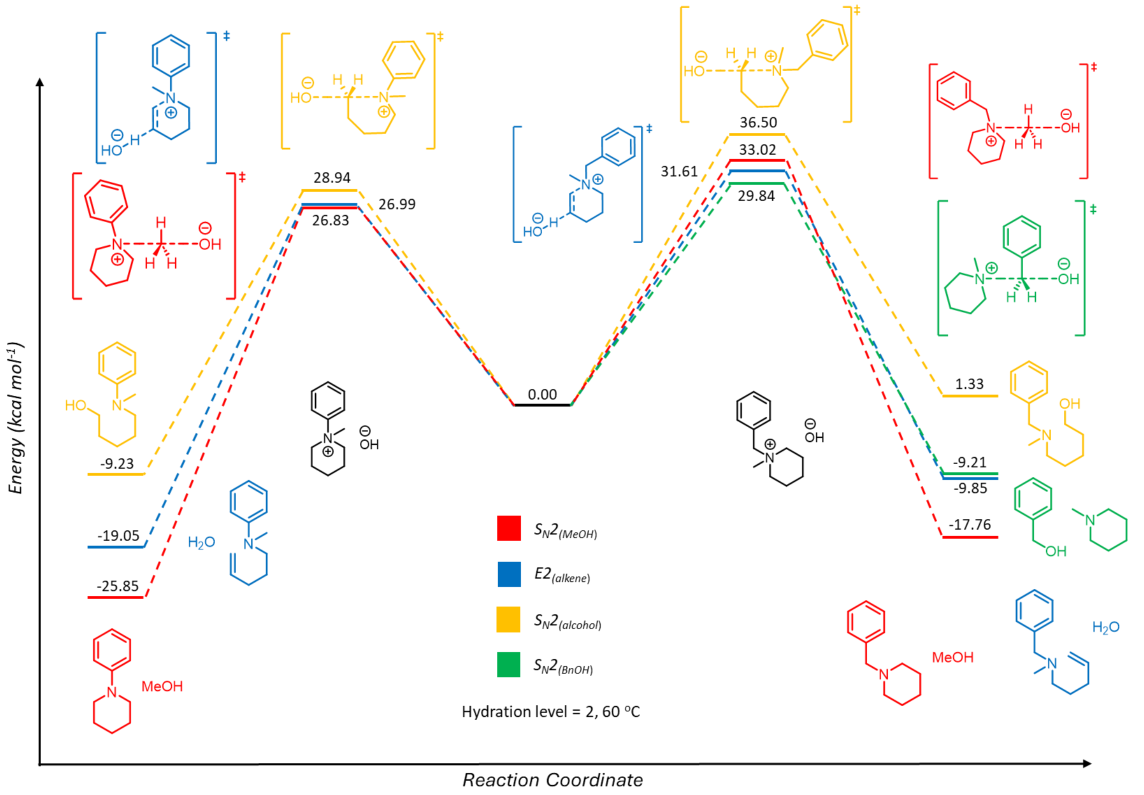

Figure S80: Gibbs free energy level diagram for **1** and **7** at 60 °C at hydration number = 2.

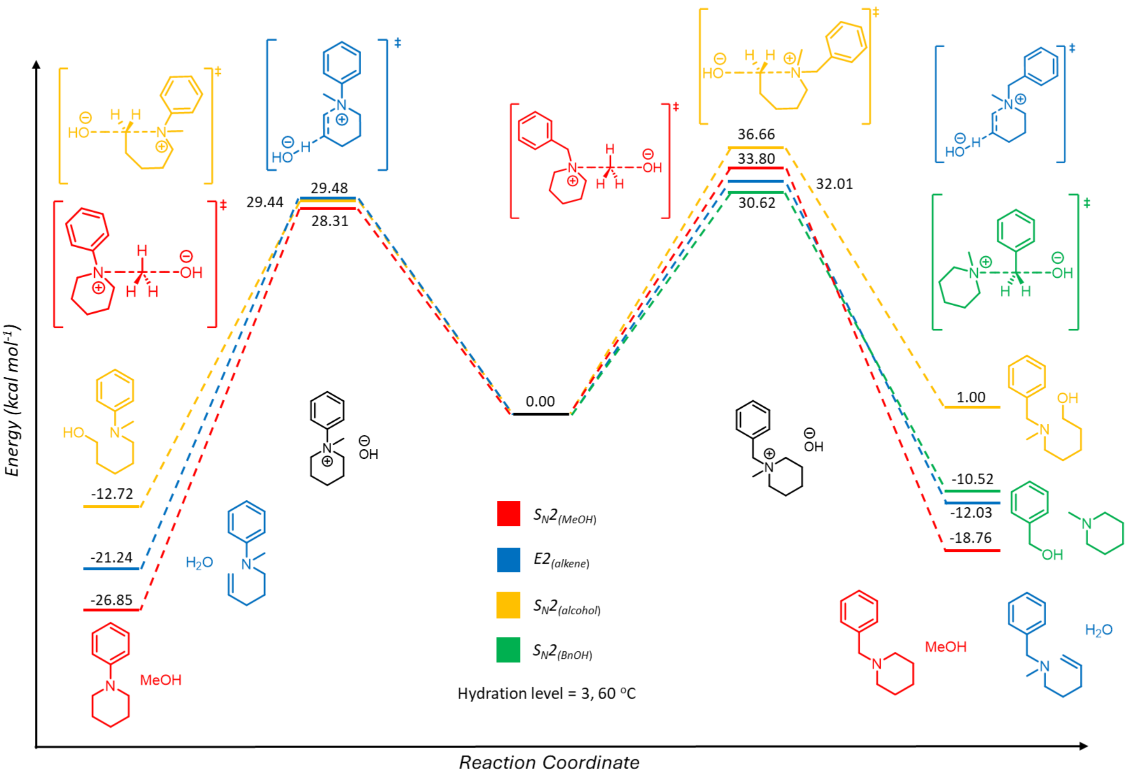

Figure S81: Gibbs free energy level diagram for **1** and **7** at 60 °C at hydration number = 3.

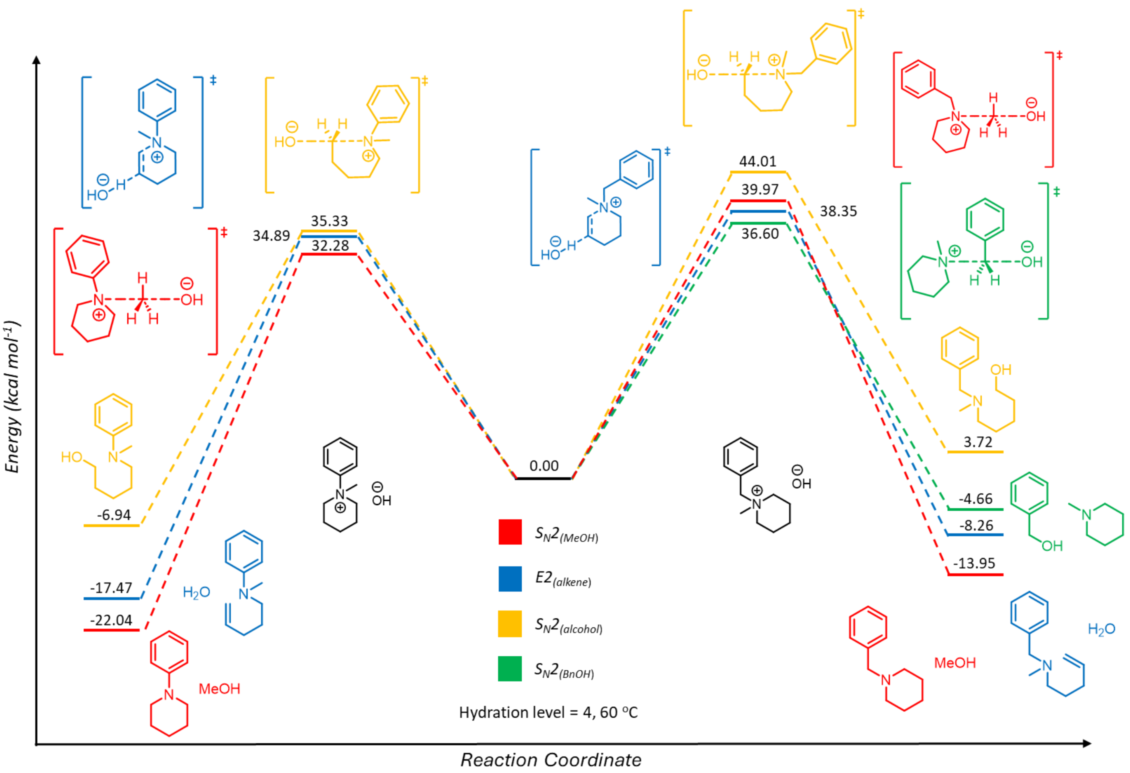

Figure S82: Gibbs free energy level diagram for **1** and **7** at 60 °C at hydration number = 4/

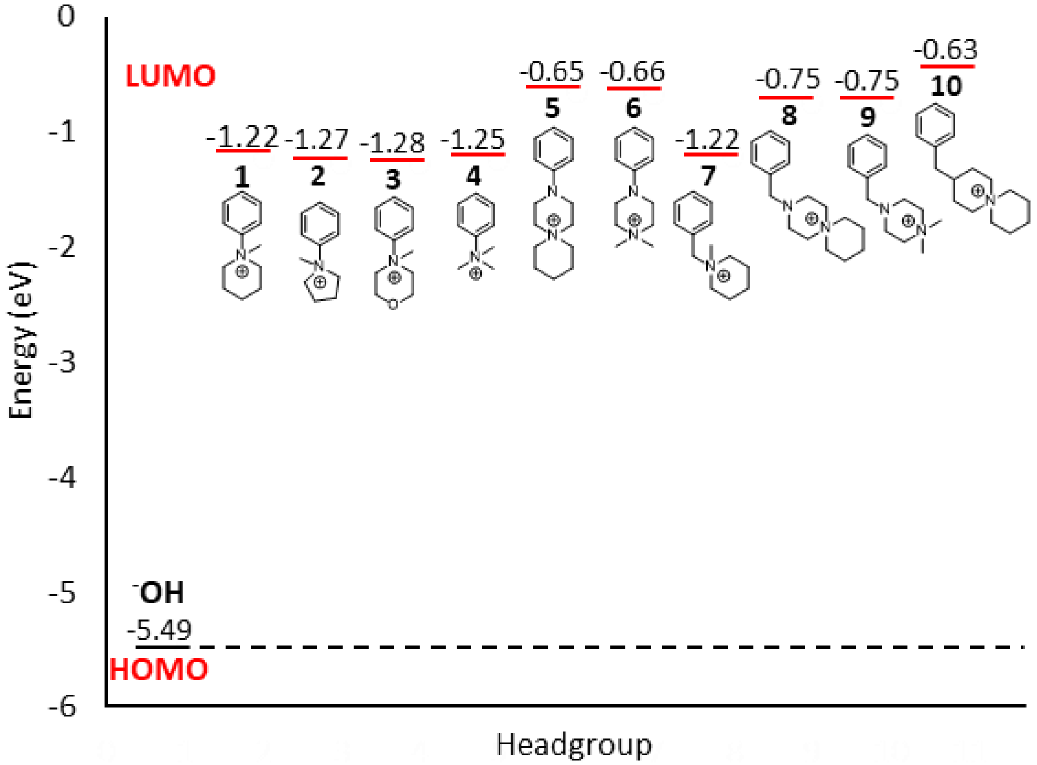

Figure S83: Calculated Lowest Unoccupied Molecular Orbital (LUMO) energies of **1 – 10** and calculated Highest Occupied Molecular Orbital (HOMO) energy of hydroxide anion.
